# Proceedings of the African Field Epidemiology Network (AFENET) Scientific Conference 17-22 November 2013 Addis Ababa, Ethiopia: plenaries and oral presentations

**DOI:** 10.11604/pamj.2015.21.208.7258

**Published:** 2015-07-22

**Authors:** Sheba Nakacubo Gitta, Allan Mwesiga, Raoul Kamadjeu

**Affiliations:** 1 African Field Epidemiology Network, Pan African Medical Journal, Kampala, Uganda; 2 The Pan African Medical Journal, Nairobi, Kenya

**Keywords:** Conference, Africa, epidemiology, field epidemiology, FELTP

## Abstract

Biennially, trainees and graduates of Field Epidemiology and Laboratory Training Programs (FELTPs) are presented with a platform to share investigations and projects undertaken during their two-year training in Applied Epidemiology. The African Field Epidemiology Network (AFENET) Scientific Conference, is a perfect opportunity for public health professionals from various sectors and organizations to come together to discuss issues that impact on public health in Africa. This year's conference was organized by the Ethiopian Health and Nutrition Research Institute in collaboration with the Ethiopia Ministry of Health, Ethiopian Public Health Association (EPHA), Ethiopia Field Epidemiology Training Program (EFETP), Addis Ababa University (AAU), Training Programs in Epidemiology and Public Health Interventions Network (TEPHINET) and AFENET. Participants at this year's conference numbered 400 from over 20 countries including; Angola, Burkina Faso, Cameroon, Central African Republic, Democratic Republic of the Congo, Ethiopia, Ghana, Indonesia, Kenya, Mozambique, Namibia, Nigeria, Rwanda, South Africa, Sudan, Tanzania, Uganda, Yemen and Zimbabwe. The topics covered in the 144 oral presentations included: global health security, emergency response, public health informatics, vaccine preventable diseases, immunization, outbreak investigation, Millennium Development Goals, Non-Communicable Diseases, and public health surveillance. The theme for the 5th AFENET Scientific Conference was; “Addressing Public Health Priorities in Africa through FELTPs.” Previous AFENET Scientific conferences have been held in: Accra, Ghana (2005), Kampala, Uganda (2007), Mombasa, Kenya (2009) and Dar es Salaam, Tanzania (2011).

Dr Claire Stanley, gave an overview of the history of global health security. She talked about promoting health, peace, stability and security. She said that Global Health Security often falls into the umbrella of what we call bio-risk management and is a combination of bio safety and bio security. According to Dr Stanley, bio safety is protecting people and the environment from dangerous pathogens whereas bio-security is about ensuring the agents are secured against misuse. She talked about Bio Security Engagement Program which works on health and security. Overall, the program tries to decrease the likelihood that dangerous pathogens intentionally or naturally cause a dangerous outbreak. She gave examples of outbreaks like “Black Death” or plague in Europe (caused by *Yersinia pestis*) which caused major socio-economic chaos, smallpox introduced in the early times and more recently of anthrax attacks, the HIV/AIDS, pandemic, Severe Acute Respiratory Syndrome (SARS) and H1N1.

