# Supplementary material for: Proceedings of the African Field Epidemiology Network (AFENET) Scientific Conference 17-22 November 2013 Addis Ababa, Ethiopia: plenaries and oral presentations
Source: Pan Afr Med J. 2015 Jul 22;21:208. doi: 10.11604/pamj.2015.21.208.7258 (PMC4595673; doi:10.11604/pamj.2015.21.208.7258)
Supplement: Related texts

# Sub article: Global Health Security and Emergency Response

**Published:** 2015-07-22

**Authors:** Jeff Borchert

Dr. Jeff Borchert spoke about biological threats that occur naturally, are intentionally produced, or are a result of laboratory accidents which constitute a growing international threat to humans in the global economy. He shared a case which happened in north-western Uganda a year ago. The case was a story of a mother who died sometime after she lost her son to “pneumonic plague”, a dangerous contagious disease. Besides, her friend died 24 hrs after the boy's burial. After the local team from Uganda's Virus Research Institute performed a blood test and found out that the result was positive for plague, they treated the villagers who were assumed to have been exposed to the disease with antibiotics. He also said global health security involves preventing an outbreak from getting out of control and that disease outbreaks indicate that we are all connected and in some ways we are all at risk. He also stated that outbreaks that happen anywhere in the world can be a risk for people everywhere in the world. He mentioned that globalization of travel food supply, rise in drug resistance including drug resistant TB were some of the sources of global security threats. He also talked about CDC's approach and implementation of global health security, and its demonstration projects in Uganda. He added that the good global health securities must make the world safe and secure from global health threats caused by infectious diseases by preventing naturally occurring outbreaks, detecting and reporting outbreaks and creating global networks that can respond effectively and mediating human suffering and loss of life that can negatively impact the economy.

# Sub article: Life and Death in Sub Saharan Africa

**Published:** 2015-07-22

**Authors:** George Mensah

Dr Mensah made a video presentation of his key note speech. He began by talking about research for health in Africa. He highlighted the scarcity of ideal local data on life and death in Africa and the crucial role of field epidemiology. His presentation then focused on the key findings of the global burden of disease in Africa and its implications. He highlighted the 2008 Bamako Call to action for research for health and mentioned the two of the principles in the document. These were: Principle 7: To strengthen health data, strengthen skill set for data analysis, planning and monitoring and Principle 8: Strengthening health research capacity and build critical mass of young researchers

Every nation should have a comprehensive research agenda at all levels. Most Sub Saharan African Countries lack national vital registration systems or local level data to inform policy decisions. Most births and deaths are unrecorded. As a result, the regional health policies are not really informed by population based data. For example, only about 1% of deaths in low-income countries are reported. In 2012, only 42/46 countries in WHO Africa had no vital registration systems at all compared to over 95% in developed countries. Due to these poor records, many of the projections in Sub Saharan Africa (SSA) are not very reliable and predictions of Cardiovascular Diseases (CVDs) and other Non-Communicable Diseases (NCDs) are perhaps exaggerated.

Many lessons can be drawn from the 2010 Global Disease Burden (GDB) study in SSA. The study showed a massive increase in many chronic NCDs between 2004 and 2030. However, these findings mask what is in SSA. The reality is 80% chronic diseases happen in SSA. The major divers of chronic disease are lifestyle and behaviour changes, nutritional transition, physical inactivity and demographic and epidemiologic transition. For example, in many parts of SSA, there is increase in adverts of fast foods and smoking of tobacco including among women.

The GDB data shows a systematic effort to quantify the comparative magnitude of health loss for 187 countries from 1990-2010 covering 291 diseases. The study identified key drivers of rapid changes in global health patterns. These were: demographic transition, cause of death transition shift from communicable to NCD, disability transition to disease that cause disability but not mortality and risk transition- shift from risks related to poverty to those of lifestyle.

On average, there was a substantial increase in the mean age of death in all SSA countries, but the causes of death had not changed substantially. They are still communicable diseases; malaria, HIV/ AIDS and lower respiratory diseases. The major causes of Disability Adjusted Life Years (DALYs) in males were underweight, indoor pollution, poor feeding, alcohol and diet. In most countries outside SSA, the DALYS from NCDs exceed 60%. Malaria, HIV/AIDS, and underweight are still responsible for most deaths and disabilities. However, there is no evidence of pandemic of NCDs. The major changes from 1990 to 2010 data include decline in major nutritional deficiencies and sanitation, while there have been increases in alcohol use, hypertension.

In conclusion, Dr Mensah stated that the scarcity of ideal data on births and deaths calls for renewed investments in epidemiology and vital registrations. The leading causes of deaths in the last 20 years have remained unchanged over the last 20 years. The overall mortality is decreasing and life expectancy has increased over last 40 years while deaths from measles and tetanus have plummeted. Although there have been dramatic declines in deaths from diarrheal disease and lower respiratory infections, some age groups are facing an increase in mortality. The use of alcohol, tobacco and hypertension are on the rise. However, health promotion and primary prevention remain preeminent.

# Sub article: Global Outbreak Alert and Response Network (GOARN)

**Published:** 2015-07-22

**Authors:** Armand Bejtullahu

The Global Outbreak Alert and Response Network (GOARN) is a global technical partnership, coordinated by WHO whose focus is outbreak response. It is made up of Technical institutions capable of providing international assistance. GOARN aims to provide rapid multi-disciplinary technical support for outbreak response and a coordinated international team providing best available expertise to Member States.

GOARN was eestablished in April 2000, with a founding group of 60 institutions. Now it comprises of over 500 partners, network hubs and network members. The Network is coordinated by a 21 member Steering Committee with the Secretariat based in WHO headquarters in Geneva. As of 20 October 2013, GOARN conducted over 130 operations and 1475 deployments.

GOARN Guiding principles include a rapid response coordinated by the Operational Support Team that requests for assistance from affected state(s). The most appropriate experts reach the field in the least possible time to carry out coordinated and effective outbreak control activities. Responses are used as a mechanism to build global capacity by the involvement of participants from field-based training programmes in applied epidemiology and public health practice such as Field Epidemiology Training Programmes (FETPs).

# Sub article: An overview of the GOARN process: Detect, assess, assist and inform

**Published:** 2015-07-22

During an International Alert, Field Response is triggered by a formal request for support from a WHO Member State. GOARN Secretariat issues an Alert, for early warning / preparedness and coordinating response planning. The mission agreed with country, and specific terms of reference for each expert. GOARN Secretariat issues a Request for Assistance to all GOARN institutions, specifying expertise, skills required for the outbreak response team.

The International response requires Institutions to submit CVs of the staff who fulfill requirements (qualifications, experience, skills). Country negotiations on support are done and a suitable candidate(s) is selected and informed. The deployment process initiated and experts deployed and mission undertaken. The final mission report submitted / formal debriefs.

Deployment administration requires getting specific documentation in order such as Current Curriculum Vitae, Passport Information, Complete online training for Basic and Advanced Security in the field, Security Clearance (arranged by GOARN, a Medical Form / Clearance and administration forms (Declaration of Interests, Biodata, Bank details, declaration of beneficiary). During the deployment process, the GOARN code of conduct respected. A WHO contract issued and GOARN covers operational costs (e.g. flight ticket, per diem, logistics). (Figure 1) An overview of the GOARN process: Detect, assess, assist and inform

**Figure 1:** An overview of the GOARN process: Detect, assess, assist and inform

# Sub article: Public Health Informatics

**Published:** 2015-07-22

**Authors:** Raoul Kamadjeu

Dr Raoul began his power point presentation by explaining the role of Information Technology (IT). He mentioned that IT changes the way in which we work, interact, communicate, generate information and use information. There are many factors that interact in various ways to affect the health outcomes of individuals within this group. These factors can be distal factors, for example, poverty and education, to more proximal factors like diet, activity, smoking, to physiological factors. You can also think of these factors as layers.

If we are to develop an understanding of how we get to these outcomes as a result of interactions between these factors, we need to be able to quantify these factors and their interactions so we can design interventions that can effectively and efficiently impact key leverage points. In the end, we cannot improve what we do not measure. For us to be able to do these measurements, we need to be able to systematically collect, manage, analyze and use data about these various factors.

We cannot improve what we do not measure. Public health is data and information driven enterprise. Data are transformed into information which is turned into knowledge for informed decision making. Public health informatics addresses public health information challenges. There is a basic knowledge-to-action assumption in public health. Measuring population parameters is a prerequisite for knowledge generation. We measure and we know, and act on what we know. Sometimes we know and we cannot act because of several factors: competing priorities, apathy, governance, limited capacities among others.

Health Information Systems (HIS) have always occupied a central role into public health practice. Because of the assumption that more information and better information is needed for good public health practice, there has been a renewed emphasis for HIS development in developing countries.

Dr Kamadjeu highlighted the challenges of the aging information systems in public health that include: difficulty pulling data out of information systems, repetitive data collection, with little reuse, investing in information systems and not meet information needs resulting in wastage of resources and data silos which may offer many opportunities for reuse but fail to do so.

Public Health Informatics (PHI) is the systematic application of knowledge about systems that capture, manage, analyze and use information to improve population health. Dr Kamadjeu stated the principles of PHI which include engage all stakeholders, put the logical before the physical, plan for interoperability and manage for accountability (borrowed from Dave Ross of the Public Health Informatics Institute)

# Sub article: Vaccine Preventable Diseases Surveillance and Response: The Role of a Public Health Institute

**Published:** 2015-07-22

**Authors:** Amha Kebede

Dr. Kebede gave a presentation on the role of a public health institute. He mentioned that the Ethiopian Health and Nutrition Research Institute (EHNRI) works closely with FMoH. The institute gathers evidence based on its research findings; it manages public health emergency; conducts health and health related surveys & surveillances; it supports the national laboratory system; it vaccinates and diagnoses productions and generates key health related performance indicators (KPI). Dr. Amha also pointed out WHO's estimation regarding annual death occurrence among infants and children who are below the age of 5, which is 8.8 million. From this figure 20% of death were due to vaccine-preventable diseases.

Pneumococcal diseases, Hib, pertussis, measles and tetanus are among the high-burden VPDs which cause high mortality in the Africa Region. However, because of vaccination, the number of cases has decreased by up to 100 percent for diseases like smallpox, diphtheria, polio (paralytic) and CRS. Dr. Amha mentioned that in 1980, Ethiopia launched EPI against measles, polio, diphtheria, pertussis, tetanus, hepatitis, *Hemophilus influenza*, rota virus, tuberculosis, and pneumonia. In the 2012/13, immunization coverage of Ethiopia for Pentavalent 3 was 87.6%, PCV3-80.4%, Measles-83.2% and full immunization-77.7%. Dr. Amha noted that there are 20 priority diseases and conditions under surveillance in Ethiopia of which 50% are vaccine preventable. These diseases are categorized in to two; immediately reportable and weekly reportable. AFP, anthrax, avian human Influenza, cholera, dracunculiasis/guinea worm, measles, neonatal tetanus, H1N1, rabies, small pox, SARS, viral hemorrhagic fever (VHF) and yellow fever which are categorized under immediately reportable. Diseases such as dysentery, malaria, meningitis, relapsing fever, typhoid fever, typhus and severe acute malnutrition are categorized under weekly reportable. This category is made according to high epidemic potential, international concern, targeted for eradication or elimination, public health importance and effective prevention and control measures.

Guidelines and plans to address these diseases are in place. Two case detection tools which are also called case definitions were presented as samples. There are also different reporting formats such as weekly reporting, case based reporting, case based laboratory reporting, line list reporting, daily epidemic reporting and GW Investigation reporting format as well as AFP case investigation form, rumor log book and E-PHEM. Immediately reportable diseases should be reported to the health center then to the woreda (district), then to the region and finally to EHNRI within two hours which means the reporting should only take 30 minutes from one place to the other. There is treatment at the health facility all week and the time line for weekly reportable diseases should be; Monday the report should go from the health facility to the wereda (district), on Tuesday from the woreda to the zone, on Wednesday from the zone to the region and on Thursday from the region to EHNRI. Dr, Amha said that he obtained the above information about the timeline from Public Health Emergency Management Guideline for Ethiopia 2012.

Telephone, fax, e-mail and rumors are the means of communication that Dr. Amha mentioned during his presentation. These channels can be used to pass the report from the health facilities to the woreda and then to the zone and finally to EHNRI.

Data management takes place through data entry and compilation, analysis and interpretation and dissemination of bulletin and collection of feedback. When there is an alert or warning that needs to be communicated to the community, then public media (TV, radio and magazine), brochures and letters can be used. Laboratory diagnosis takes place to detect diseases, to confirm outbreaks and identify genetic and characterization of strains. Polio, measles, rubella, hepatitis, influenza, yellow fever, rotavirus and meningitis can be diagnosed in the laboratory. Rapid assessment, establishment and training of rapid response teams and outbreak investigation conducted on epidemiological and laboratory diseases. Besides, coordination between Ministry of Health, EHNRI, some agencies and PHEMC took place to mobilize resource, share information and activity responses among one another. Dr. Amha also noted that, vaccination is provided for diseases such as Meningitis, Measles, Yellow Fever and Polio. Social Mobilization and Health Education uses to mobilize community and to create awareness among health workers.

Dr. Amha noted that for the case management to take place, treatment control should be provided. Besides, health workers should be trained and emergency drugs should be supplied. In order to prevent epidemic contamination, there is need to isolate and quarantine cases and suspects, conduct vector control, hygiene and sanitation. The post outbreak assessment identifies gaps and documents lessons learnt like preparedness, surveillance and response. The assessment also helps to implement corrective measures to prevent problems of similar outbreaks in the future. Dr. Amha concluded his presentation by noting that the public health research should contain vaccination coverage survey, sentinel surveillance and serological and entomological survey.

# Sub article: Immunization in Africa: Progress and Challenges

**Published:** 2015-07-22

**Authors:** Yemane Berhane

Expanded Program for Immunization (EPI) in Africa now focuses on building sustainable immunization systems to protect children against common VPDs, and serve as vehicle for other child survival interventions. The intervention initially distributed six vaccines: diphtheria, tetanus, pertussis, poliomyelitis, TB, and measles. Currently it integrates other vaccines: Yellow fever, hepatitis B, Hib, PCV, rotavirus vaccines, HPV, MenA, MCV2, etc. Prof. Yemane in his study showed that in 2011, 81% of non-vaccinated infants of the region are located in ten countries: Nigeria, Ethiopia, DRC, Chad, South Africa, Cameron, Uganda, Cote d'Ivoire, Mozambique and Niger. He also discussed WHO’s “Global Vaccine Action Plan 2011-2020”, which he stated as achieving a world free of Poliomyelitis, meeting the vaccination coverage in every region, country and community, exceeding the millennium development goal target for reduction of child mortality, meeting global and regional elimination targets and develop and introduce new and improved vaccines and technologies.

Prof. Yemane also indicated that the four main immunization program priorities i.e. Polio Eradication, Accelerate Disease Control, Reach the Unreached and Improve Data Quality. He also stated the five operational components of an EPI programme; Vaccine Supply & Quality, Logistics, Service Delivery, Surveillance, Advocacy & Communication. Additionally, the study shows that accurate and reliable data leads to improved decisions which again lead to efficient use of resources. Regarding issues related to immunization in Africa, Prof. Yemane pointed out sstrategies to increase routine immunisation coverage, monitoring epidemiology of VPD and tailoring strategies accordingly, advocacy and creation of demand for immunization, capacity for outbreak investigation and response, surveillance quality and documenting best practices, vaccination coverage data quality, introduction of new vaccines: when and how, strengthen advanced data analysis of routinely collected information and operational research are core methods to tackle the problems.

He also explained the major challenges of immunization in Africa and what the role of professional networks in reaching immunization targets could be. According to Prof. Yemane the challenges are stalling or receding routine immunization coverage, resurgence of measles outbreaks, resurgence of epidemic polio in the Horn-of-Africa, insufficient demand creation, weak links with communities and their leaders, insufficient human resource capacity at all levels with rapid turnover of staff, and gaps in supportive supervision, poor data quality/use for decision-making, lack of clear leadership and commitment, inadequate funding and inadequate supply and logistics systems. Training, research, advocacy and collaboration at program and health facility level through internship or other mechanisms are the roles of networks in reaching immunization targets suggested at the end of the study.

# Sub article: Community Resistance to Oral Polio Vaccine in Nagero County, South Sudan

**Published:** 2015-07-22

**Authors:** Mathew Lado Jocker

**Affiliations:** 1 Sudan Immunization and Field Epidemiology Training Project

## Abstract

**Background:**

The Republic of South Sudan (RSS) is made up of 10 states and 80 counties. Western Equatoria State (WES) experienced polio outbreak in 2009, of which 11 children were paralyzed. Nagero County is one of the 10 counties of WES. The county is sparsely populated and shares a long border with the Central African Republic, which puts RSS at risk for importation of polio. Based on national data from previous house-to-house vaccination campaigns, Nagero is has a high parental refusal rate against polio vaccine. During the March 2013 house-to-house campaign (NIDs), we conducted a study of households that refused to have their <5 children vaccinated.

**Methods:**

NID teams provided a list of households in 2 Payams that refused vaccination. We followed-up with those household to conduct face-to-face interviews with parents or adult caregivers. We developed a structured questionnaire to collect information on demographic and household characteristics and to document why they refused to have their child or children vaccinated.

**Results:**

Nineteen percent of households (n = 226) in the 2 Payams refused vaccination. Twenty percent of the children in those households were unvaccinated infants (<1 year old). All households (n = 43) consented to interview. Analyses of the interview data showed that parents refused due to 1) seeing or hearing about adverse events post-vaccination (53%), 2) thought all vaccines were dangerous (33%), or 3) held traditional beliefs that other medicines were more effective in protecting their children (27%). Additional analyses showed that some (20%) would refuse oral polio vaccine only, whereas another 20% would refuse all vaccines for their children. Follow-up interventions by NID teams did not convince any of the households to allow vaccination of their children.

**Conclusion:**

This snapshot of polio vaccine refusals is instructive. We see that the reasons for refusal are various, but are not so entrenched that tailored and specific community education and outreach efforts cannot overcome. Social mobilization and education should be strengthened to educate the community leaders, women's groups, and others to understand the role that vaccines play in keeping communities healthy. Involvement of high profile community leaders to address the issues of refusals within communities is also important.

# Sub article: Investigation of a Measles Outbreak in the Nkolndongo health District, Yaoundé Cameroon: A case-control study

**Published:** 2015-07-22

**Authors:** Ndode Corlins Ebontane

**Affiliations:** 1 Central African Field Epidemiology and Laboratory Training Program

## Abstract

**Background:**

Measles is a highly infectious viral disease with children below 15 years being mostly at risk. It remains a public health priority in Cameroon. In 2012, the Nkolndongo health District (NHD) notified 214 of the 2422 Measles cases reported in the Centre region. Since the beginning of 2013, sporadic Measles cases have been reported in the NHD and in March, three suspected cases were confirmed positive. With district Measles vaccination coverage of 76% by the end of the first trimester of 2013, we conducted the investigation to determine factors associated with contracting Measles.

**Methods:**

A 1:1 unmatched case control study was conducted. A case was a person residing in NHD who met with the standard WHO Measles case definition from 01st January to 10th April 2013. A control was any person residing in the same community not having a history of Measles during the same period. A structured interviewer administered questionnaire was used to solicit information from parents of cases and controls. Written Authorization and verbal consent were sought. Epi-Info was used to calculate frequencies, odds ratios and perform logistic regression to control for confounding variables.

**Results:**

A total of 22 subjects made of 11 cases and 11 controls were recruited. Males accounted for 14 (63.6%) subjects. The median age of subjects was 3 years. There was no statistical significant difference in the socio-demographic characteristics of both cases and controls. All cases presented with fever and cutaneous eruptions, the mean duration for seeking medical attention was 2 ± 1.2 days (Q1 = 1; Q3 = 3). The case fatality was 9.09%. No subject received a booster Measles dose. Vaccination status (OR: 0.074 p = 0.03) and contact with a suspected Measles case (OR: 7.88 p = 0.04) were associated with contracting Measles. Travelling, vitamin A supplementation and health education were found not associated with contracting Measles.

**Conclusion:**

Measles in the Nkolndongo health District was favoured by the absence of Measles vaccination and contact with a suspected Measles case. We suggested health education in favour of vaccination and routine vaccination be re-enforced. Following this study, a vaccination campaign was conducted and active surveillance re-enforced.

# Sub article: Measles Outbreak Investigation and Response in Arsi Zone, Oromia Region

**Published:** 2015-07-22

**Authors:** Daba Feyissa

**Affiliations:** 1 Ethiopia Field Epidemiology and Training Program

## Abstract

**Background:**

Measles is a highly infectious vaccine preventable disease. In 2012, Honkolo-Wabe, Lemu-Bilbilo and Shirka District s were among the Measles affected districts in Arsi zone of Oromia region of Ethiopia. In November 2012, an outbreak was reported and we investigated the outbreak to characterize it, identify the risk factors, and implement appropriate public health control measures. We also determined Measles vaccination coverage in the districts.

**Methods:**

A suspected case of Measles was any person presenting with fever and maculopapular rash and a cough, coryza or conjunctivitis while a confirmed case was IgM positive for Measles. We conducted a case-control study of 53 cases with 106 unmatched community controls. Line lists, observation of cold chain, key informant interviews at different levels were used to collect data. Data entry and analysis was performed using Epi-Info version 7 and Microsoft Excel.

**Results:**

A total of 170 Measles cases and one death were reported. The outbreak was confirmed by IgM antibody. The index case was seen in Lemu-Kara kebele on Oct.18, 2012. Nine kebeles were affected in three Districts. The overall attack rate was 38 per 100,000 populations and age specific attack rate for age group 5-9 was 104 per 100,000 populations. Measles vaccine coverage ranges from 83.3% - 99.6% in affected Districts. Sixty six percent of cases and 18.9% controls were unvaccinated. Un-vaccination status was significant associated with Measles cases the [OR= 17.5; 95% CI 6.23-51.24]. Mother‘s educational and number of under five children in the family were significantly associated with the Measles cases the [OR= 8.21; 95% CI 3.66- 18.66], and [OR = 13.6; 95% CI 5.76-32.83] respectively.

**Conclusion:**

Un-vaccination, mothers’ education and the number of fewer than five children in the family, may have increased susceptibility. We recommend improved routine and campaign Measles immunization; strong ongoing active case surveillance of febrile rash illness; health education on treatment and prevention of Measles to be enhanced and continued in the community by health workers.

# Sub article: Meningococcal Meningitis Outbreak in Hawassa City, Southern Nations Nationalities and Peoples Region Ethiopia, 2013

**Published:** 2015-07-22

**Authors:** Kidanie Yeshitila

**Affiliations:** 1 Ethiopia Field Epidemiology and Training Program

## Abstract

**Background:**

In Ethiopia meningococcal meningitis epidemics occur cyclically every three to five years in several regions that are located within the African meningitis belt. In January 2013, Hawassa City Administration reported an increase in persons with fever, headache, and neck stiffness. We investigated to confirm the outbreak, identify risk factors and implement control measures.

**Methods:**

We defined a suspect case of meningococcal meningitis as any person with sudden onset of fever and neck stiffness in Hawassa City from 22 January to 17 February 2013. We did active case surveillance for suspected cases from Addare and Referral Hospitals and collected cerebrospinal fluid (CSF) specimens from cases. We used Gram stain, latex agglutination and culture for confirming tests. We performed a case control study and compared confirmed and probable cases with hospital - unmatched controls. We included all admitted cases that a physician diagnosed as meningitis.

**Results:**

We identified 87 suspected cases and 4 deaths (attack rate: 26.1/ 100,000, case fatality rate: 4.6). The age adjusted attack rate was highest among persons aged 15-29 years (AAR 37.5/100,000). Of eight confirmed cases we isolated serogroup A (4), C (1) and W135 (1) and Gram-positive diplococci (2) from CSF specimens. Out of 27 cases and 54 controls having a history of recent acute respiratory infection within ten days was significantly associated with contracting meningococcal meningitis (odds ratio [OR]: 3.7, 95% confidence interval [CI]: 1.4-9.8). Living in single rooms with four or more family members was significantly associated with disease (OR: 7.08, 95% CI: 1.6-31.3).

**Conclusion:**

This investigation confirmed an outbreak of meningococcal meningitis and identified recent acute respiratory infection and overcrowded housing as significant risk factors for contracting meningococcal meningitis. We can reduce the risk though community education on personal hygiene for prevention of infections. We recommended community education and a mass vaccination campaign.

# Sub article: Measles Outbreak investigation in Tselemti Woreda, North Ethiopia, 2013

**Published:** 2015-07-22

**Authors:** Goitom Mehari

**Affiliations:** 1 Ethiopia Field Epidemiology and Training Program

## Abstract

**Background:**

Measles has been targeted for elimination in many regions of the world. It is a highly contagious viral infection and most vaccine strategies are targeted at children. We received reports of a suspected Measles outbreak from Tselemti Woreda. Investigation was conducted to confirm the presence of outbreak, identify risk factors, and institute control measures.

**Methods:**

We used the World Health Organization clinical case definition for a suspected Measles patient as any person with fever and maculopapular rash and cough, coryza, or conjunctivitis or any person in whom a clinician had suspected Measles. From March 28 up to April 7/2013 we investigated all suspected Measles patients in the affected Woreda, and we conducted unmatched case-control study (1:3). Five blood specimens were collected for laboratory confirmation.

**Results:**

We identified 34 Measles patients; 5 were laboratory confirmed and 29 were epidemiologically linked. All five blood samples tested positive for Measles specific IgM. The median age for Measles patients was 21 years (ranges 2 months - 50 years) and 70.6% of the patients were more than fifteen years old. The vaccination rate among cases was 5.9%. We compared 30 case patients to 90 control subjects and found that recent travel to areas with active Measles cases (AOR = 9.4, 95% CI: 2.9-29.9) and Living in a room with more than five people (AOR = 4.3, 95%: 1.5- 12.8) were risk factors for developing Measles but vaccination status (AOR= 0.15, 95% CI: 0.03-0.78) was protective factor.

**Conclusion:**

There was outbreak of Measles in Tselemti Woreda. Seventy percent of the Measles infections in this outbreak occurred in adults. The significant risk factors identified were crowdedness and recent travel to areas with active Measles cases. Vaccination and awareness on Measles transmission were recommended. Further studies are needed to assess the epidemiology of Measles in Tigray to guide vaccination policy in adults.

# Sub article: Sporadic cases of Meningococcal Meningitis Serogroup W-135, Ethiopia 2013

**Published:** 2015-07-22

**Authors:** Gemechu Beffa

**Affiliations:** 1 Ethiopia Field Epidemiology and Training Program

## Abstract

**Background:**

Meningococcal meningitis is a serious bacterial infection of the meninges often caused by *Neisseria meningitidis*. Epidemics occur in 8-12 year cycles, usually in the dry season, across the African meningitis belt. In February 2013, West Arsi zone reported a suspected meningitis outbreak to Regional Public Health Emergency Center. We investigated to confirm the etiology, identify risk factors, and establish control measures.

**Methods:**

We defined a suspected case as any person with sudden onset of fever (>38.5°C rectal or 38.0°C axillary) and one of the following signs: neck stiffness, flaccid neck, bulging fontanel, convulsion or other meningeal sign and confirmed case if N. meningitidis was identified from the cerebrospinal fluid (CSF) of a suspected case by culture, PCR or agglutination test. From January 23 -April 17, 2013 we conducted a case investigation to identify suspected and conformed cases and a case-control study.

**Results:**

A total of 99 cases and 3 deaths were occurred from January 23- April 27, 2013. The median age was 12 years (range 3 months to 68 years). Majority (89.9%) of the cases were below 30 years of age and children under five years of age were the most affected (28%) and Attack Rate (AR = 4.2/100000). By agglutination test and PCR, ten (59%) patients with *N. meningitidis* were confirmed as serotype A, 6(35%) patients were confirmed as serotype W135 and 1(6%) patient was confirmed as mixed serotype. Twenty-four confirmed and suspected meningococcal meningitis cases and 96 community-matched by sex, age and place of residence controls were included in the case control study. Recent travel to an area where patients with meningitis were reported (Odds Ratio (OR): 10.0, 95% Confidence Interval (CI): 3.7-27.3), attending in the occasion of gathering of population (OR: 7.7, 95% CI: 2.9-20.6) and a history of upper respiratory tract infection (OR: 7.2, 95% CI: 2.6-19.9) were significant risk factors.

**Conclusion:**

We verified sporadic cases of meningococcal meningitis in the areas. Risk factors Incidence of disease was highest in children under five years of age. This was the first season that W135 was identified in Ethiopia. Further surveillance for W135 should be conducted in Ethiopia to guide vaccination policy.

# Sub article: Epidemiology of Meningococcal Meningitis - Ethiopia, 2005-2012

**Published:** 2015-07-22

**Authors:** Elsabetee Sorassa

**Affiliations:** 1 Ethiopia Field Epidemiology and Training Program

## Abstract

**Background:**

Globally an estimated 500,000 cases and 50,000 deaths of meningococcal disease occur each year. The highest rates of meningococcal disease are found in the “meningitis belt,” which extends from Senegal to western Ethiopia. A new meningococcal vaccine program for serogroup A was introduced in 2010 in three countries within the meningitis belt and produced a dramatic decline in meningitis cases. We analyzed surveillance data to describe the epidemiology of meningococcal meningitis in Ethiopia in anticipation of the implementation of a national meningococcal vaccination program.

**Methods:**

We analyzed routine weekly surveillance data reported to the national level from 2005-2012. We defined a suspected case of meningitis as a patient with sudden onset of headache, fever (>38.50C rectal or 380C axillary), and one of the following signs: neck stiffness or other meningeal signs or altered consciousness.

**Results:**

We identified 8,866 suspected meningitis cases between 2005 and2012. The annual incidence of *Meningococcal meningitidis* was 1.4/100,000 and there were 174 deaths (case fatality rate of 2%). The highest incidence (13.3/100,000) was from Harari Region, which is located in eastern Ethiopia outside the meningitis belt. Thirty-five percent (3146) of suspected cases and 72% (126) of deaths were reported during epidemics. During epidemics 1444 suspected cases (46%) were aged 15-44 years old (age adjusted attack rate 2.9/100,000).

**Conclusion:**

We determined that the highest rates of meningococcal meningitis in Ethiopia occurred outside the traditional borders of the meningitis belt. In anticipation of widespread meningococcal vaccination campaigns, epidemiological and laboratory surveillance should be strengthened throughout Ethiopia to more clearly delineate high-risk areas for meningococcal meningitis in Ethiopia.

# Sub article: Trend of Rubella Cases in Namibia, 2006 - 2012

**Published:** 2015-07-22

**Authors:** Emmy Ndevaetela

**Affiliations:** 1 Namibia Field Epidemiology and Laboratory and Training Program

## Abstract

**Background:**

Rubella is an acute contagious infection caused by the Rubella virus. It presents with rashes that resemble those of Measles. Though Rubella is usually mild in children, infection in the first trimester of pregnancy is associated with adverse pregnancy outcomes including abortion, stillbirth and congenital Rubella syndrome (CRS). Globally, 110,000 babies are estimated to be born with CRS. The World Health Organization (WHO) has recommended that countries introduce Rubella vaccination. In order to generate baseline information to determine the need to introduce the vaccine in the country, all suspected Measles cases are tested for both Measles and Rubella IgM. We conducted this study to determine the proportion of samples submitted for Measles that tested positive for Rubella, to determine the trend of the Rubella and to describe demographic characteristics of people who tested positive for Rubella IgM.

**Methods:**

We conducted a descriptive study in which we reviewed the Namibia National Institute of Pathology (NIP) laboratory Measles database from 2006 to 2012. Rubella positive cases were identified and the data entered into Epi-Info 7software. We analyzed the data by person, place.

**Results:**

Of 3,171 samples tested, 645(20%) tested positive for Rubella. Of the 645, 351(54.4%) were females. The number of Rubella cases for 2006, 2007, 2008, 2009, 2010, 2011 and 2012; were 8 (1.2%), 21 (3.3%), 3 (0.5%) 13 (2.0%) 293 (45.4%), 263 (40.7%) and 44 (6.8%) respectively. One hundred and nineteen cases (18.4%) of the cases were less than five years old a total of 603 cases (93.5%) were less than 15years. Out of the 13 regions in the country, Hardap, Otjozondjupa and Ohangwena regions had a total of 333 (51.6%).

**Conclusion:**

A significant proportion of suspected Measles cases tested positive for Rubella IgM. Majority of the cases are females and children. There was a drastic increase in the number of Rubella cases in 2010 and 2011. We recommended inclusion of Rubella in the priority disease list for surveillance and that Namibia should consider introducing Rubella vaccination as part of the expanded program on immunization.

# Sub article: Measles Outbreak investigation in Sokoto State - Nigeria, February, 2013

**Published:** 2015-07-22

**Authors:** Nuruddeen Aliyu

**Affiliations:** 1 Nigeria Field Epidemiology and Laboratory and Training Program

## Abstract

**Background:**

Measles remains one of the leading causes of death among young children, despite the availability of a safe and cost-effective vaccine. In 2011, there were 158,000 Measles deaths globally-about 430 deaths every day or 18 deaths every hour. According to World Health Organization, more than 95% of Measles deaths occur in low-income countries with weak health infrastructure. On 7th February, 2013 a health worker reported a suspected outbreak of Measles in eastern and western part of Sokoto. We investigated to verify the existence of the outbreak, define magnitude and scope, identify possible risk factors and recommend control measures.

**Methods:**

We defined a case as any person with fever and rash (maculo-papular) with any of cough, coryza or conjunctivitis living in the affected communities from 4th January to 17th February, 2013. We collected 5 blood specimens for testing of IgM antibody. We reviewed medical records of patients in health facilities to identify cases and conducted active case search in the affected communities. We describe the outbreak by time, place and person and conducted a case-control study to determine possible risk factors for Measles.

**Results:**

A total of 298 cases with 15 deaths (case fatality rate = 5.0%) were recorded of which 76% (226 cases) were from one village. Majority of cases (72.7%) were under five years of age of which 66.6% constitute 1-4 years of age and males constitute 58% of cases. 78% of Measles cases and deaths were never immunized, 19% had one dose while 3% received a second dose. First case started on 4th January, 2013 who was a 6 year old girl from Kebbe. 60% (3) of serum specimens tested positive for Measles IgM. We identified 40 cases from door- door case search. Individuals that had contact with Measles case are 12.9 times more likely to get Measles than those without contact (odds ratio = 12.9, 95% C.I = 4.40-37.9).

**Conclusion:**

An outbreak of Measles occurred in eastern and western part of Sokoto State. Very low immunization coverage may be associated with Measles. As public health action a mass vaccination for all children 9 months following our recommendations, was conducted.

# Sub article: An outbreak of Measles in Kubau Local Government Area, Kaduna State, Northern Nigeria, February, 2013

**Published:** 2015-07-22

**Authors:** Fatima Cheshi

**Affiliations:** 1 Nigeria Field Epidemiology and Laboratory and Training Program

## Abstract

**Background:**

Measles is a highly infectious vaccine preventable disease. On 26th January, 2013, 14 suspected cases of Measles were reported in Kubau Local Government Area (LGA), Kaduna State, Nigeria. We conducted an investigation to confirm the diagnosis, establish the occurrence and cause of the outbreak, and control the outbreak.

**Methods:**

We conducted an unmatched case-control study. We defined a case as any resident of Kubau LGA with fever, rash, and either cough, conjunctivitis or coryza, between 1st January, 2013 and 14th February, 2013. We collected 7 blood specimens from suspected cases to test for Measles IgM, reviewed patient records in Primary Health Care Centre Kubau, and searched for additional cases in the community. We administered a structured questionnaire to caregivers of 83 cases and 83 controls to obtain information on health and immunization status of cases and controls. We assessed routine immunization activities in the LGA. We described the outbreak by time, place and person and identified risk factors associated with the outbreak.

**Results:**

A total of 226 cases and 3 deaths were identified (attack rate: 1.68%, case fatality: 1.31%). Median age for cases was 24 months (range: 3-108 months). 115 (51%) cases were females and 102(45%) were aged 21-40 months. Of the 7 specimens collected, 2(28.6%) were positive. The outbreak started on 25th January, 2013 and peaked on 3rd February, 2013 with 46 cases. The incidence was highest (54.4%) in Kargi settlement. Measles immunization coverage in Kubau LGA was 42-58%. Cases were more likely than controls to be unvaccinated against Measles (odds ratio: 2.79, 95% confidence interval: 1.35 -5.78). Only 39.8% of respondents knew how Measles is contracted.

**Conclusion:**

Low immunization coverage in Kubau LGA may have been the reason for the Measles outbreak. We recommended improvement in routine and supplementary immunization, regular and sufficient supply of vaccines to the PHCs by Kaduna State Ministry of Health and massive community awareness campaign. The health department began to implement these recommendations, by vaccinating all under-five children, resulting in the control of the outbreak.

# Sub article: Measles Outbreak in Birnin Gwari Local Government Area, Kaduna, Nigeria 2013

**Published:** 2015-07-22

**Authors:** Abbott Samuel

**Affiliations:** 1 Nigeria Field Epidemiology and Laboratory and Training Program

## Abstract

**Background:**

Measles is a highly infectious viral disease that causes high morbidity and mortality mostly among children. A suspected Measles outbreak was reported in Birnin Gwari, Kaduna state in February 2013. We conducted an investigation to confirm, describe, assess factors associated with the outbreak and determine immunization coverage.

**Methods:**

We conducted 1:1 unmatched case-control study. Active case search was conducted in hospital and communities; a control was selected in the same neighborhood. Questionnaires were administered to caregivers of cases and controls to obtain information on socio-demography and risk factors. Immunization status was assessed using immunization cards and/or caregiver recall. Blood samples were collected from 13 cases for laboratory confirmation.

**Results:**

A total of 83 cases and 83 controls were recruited. Males constitute 53% and 47% of cases and controls respectively. The mean age of the cases and controls were 22.7 + /- 12.4 months and 27.9 +/- 13.9 months respectively. Eight of the 13 samples were Measles IgM positive. Being unvaccinated against Measles (OR= 9.4, 95% CI 3.32, 33.0) was the major risk factor for the outbreak. Immunization coverage among cases and controls were BCG 42%, DPT1 27%, DPT3 22% and Measles (18%). Reasons for lack of immunization were rejection (38%) and ignorance (33%).

**Conclusion:**

Low Measles immunity was responsible for this epidemic. We recommend supplemental Measles vaccination, strengthening of routine immunization and public enlightenment on the usefulness of immunization against vaccine preventable diseases in Birnin Gwari.

# Sub article: Mumps Outbreak in Dagahaley Refugee Camp-Dadaab, Kenya July 2012

**Published:** 2015-07-22

**Authors:** Adam Hajia

**Affiliations:** 1 Kenya Field Epidemiology and Laboratory and Training Program

## Abstract

**Background:**

Mumps is a viral disease that results in 2000 cases per 100,000 populations globally each year. In May 2012, the Kenyan Ministry of Public Health and Sanitation received reports of three deaths among children in a refugee camp suspected to have mumps. We conducted an investigation to confirm the etiology and characterize the outbreak.

**Methods:**

We conducted a retrospective review of hospital records in Dagahaley refugee camp and generated a line list. using a World Health Organization (WHO) suspected case definition of Mumps;acute onset of unilateral or bilateral swelling of the parotid or salivary glands lasting more than 2 days without other apparent cause in persons of any age residing in Dagahaley refugee camp from 1st April to 26th July 2012. Suspect cases were contacted and interviewed using a standardized questionnaire to obtain socio-demographic, clinical presentation and treatment information. Nasopharyngeal/Oropharyngeal swabs and blood samples were taken from consenting patients and tested for mumps by serology and cultured for diphtheria. Data was analyzed using MS excel and Epi-Info 7.

**Results:**

We line listed 32 suspect cases and administered questionnaire to 19 patients; 17 (53%) were male and median age was 7 years (range: 2-30 years). Nine (47%) were residing in Block A11, eleven (58%) had not completed primary immunization and fifteen (79%) reported to have come in to contact with a person with similar symptoms. Ten (67%) cases reported contacts occurred in religious schools. Naso-oropharyngeal swabs and blood samples were taken from five (21%) suspected patients. Three were both IgM and IgG positive for mumps while, one case was positive for IgG and indeterminate for IgM. None of the cultures isolated *Corynbacterium diphtheria*.

**Conclusion:**

We confirmed the etiology of the neck swelling to be mumps. Majority of children affected were not fully immunized, religious learning institutions being the commonest sources of contact. Isolation of cases during outbreaks, strengthening of routine vaccination and a prevalence study to establish the magnitude of mumps in Kenya is recommended.

# Sub article: Laboratory Confirmed Meningitis cases in Ohangwena Region of Namibia, 2007-2011

**Published:** 2015-07-22

**Authors:** Festus Kuushomwa

**Affiliations:** 1 Namibia Field Epidemiology and Laboratory and Training Program

## Abstract

**Background:**

Meningitis is a highly fatal disease that may result from bacterial, viral or fungal infection of the meninges. The case fatality rate of bacterial meningitis may be as high as 34%. About 50% of those who recover from the disease suffer from various neurological deficits. Laboratory data on meningitis in the Ohangwena region has never been analyzed. We conducted this study to describe the trends, identify the common causes of meningitis and the case fatality rates associated with the various causative agents in the region.

**Methods:**

We conducted a descriptive study in which we reviewed meningitis registers, meningitis case investigating forms and laboratory records on meningitis from 2007 to 2011 in all the 32 health facilities in the region. We identified all suspected cases of meningitis, all cases for which samples were sent to the laboratory for testing and all positive cases. We entered the data in Epi-Info7 and analyzed trends, calculated the proportions of the various etiological agents and case fatality rates associated with the various etiologic agents.

**Results:**

Of the 488 suspected cases of meningitis for the period under consideration, 379 (78%) were tested and 97 (26%) were positive for various types of meningitis. The number of laboratory confirmed meningitis cases in 2007, 2008, 2009, 2010 and 2011 were 16(16%), 13 (13%), 27 (28%), 20 (21%) and 21 (22%) respectively. The identified etiological agents of meningitis were *Neisseria meningitidis* 47 (48%), *Streptococcus pneumonia* 27 (28%), *Cryptococcus neoformans* 11 (11%), *Haemophilus Influenza* Type B 8 (8%), *Staphylococcus aereus* 3 (3%), *Borrelia vincenti* 2 (2%) and *Mycobacterium Tuberculosis* 1 (1%). In all there were 24 deaths hence the overall case fatality rate was 25%. Case fatality rates for Meningococcal, Streptococcal, Cryptococcal and *Haemophilus influenza* meningitis were 32%, 16%, 36% and 13% respectively. There were no mortalities associated with the other etiologic agents.

**Conclusion:**

The most common causes of meningitis in the region were *Neisseria meningitidis*, Streptococcus pneumoniae and *Cryptococcus neoformans*. Cryptococcal and meningococcal meningitis had the highest case fatality rates. We recommended vaccination against meningococcal meningitis and sensitization of clinicians on the common causes of meningitis in the region with the view to ensuring early diagnosis and improving case management.

# Sub article: Low Birth Weight as Predictor of Infant mortality in rural Ghana; Results from a Community Level Population Based Prospective Cohort Study

**Published:** 2015-07-22

**Authors:** Maureen O'Leary

**Affiliations:** 1 Ghana Field Epidemiology and Laboratory and Training Program

## Abstract

**Background:**

Low birth weight; (LBW) infants (weighing <2500g at birth) have a mortality rate twenty times that of non-LBW infants. In Sub-Saharan Africa, most studies on mortality among LBW infants have been conducted in the neonatal period, consequently accurate estimates on the risk of mortality in this group are lacking. We aimed to estimate the prevalence of LBW and its associated risk of mortality, to inform strategies to minimize mortality associated with LBW.

**Methods:**

This is a prospective cohort study using data collected for Neovita, a double-blind randomised control trial of neonatal vitamin A supplementation in the Brong-Ahafo region of Ghana. Infants whose mothers resided in the study area, who were born between August 2010 and November 2011, who were weighed within 3 days of delivery and whose vital status was known at 365 days of age were eligible for inclusion in this analysis. Mortality rates in the neonatal, early, late and overall infant periods for LBW and non-LBW infants were calculated and the risk of mortality associated with LBW in each period was determined using multivariable logistic regression analysis.

**Results:**

Of 27111 infants screened, 23190 (85.5%) were eligible for inclusion in the analysis. Of these, 3645 (15.7%) were classified as LBW. Comparing LBW to non-LBW infants, the mortality rate was 1) 34.6/1000 live births and 7.3/1000 live births in the neonatal period; 2) 25.5/1000 live births and 7.9/1000 live births in the early infant period; 3) 11.0/1000 live births and 6.6/1000 live births in the late infant period; and 4) 71.1/1000 live births and 21.8/1000 live births throughout infancy. When adjusted for socio-demographic, maternal, infant and environmental factors, the odds ratio for mortality among LBW infants compared to non-LBW infants was A) 4.06 (95%CI: 3.11-5.31) in the neonatal period; B) 3.25 (95%CI: 2.46-4.30) in the early infant period; C) 1.72 (95%CI: 1.18-2.50) in the late infant period and D) 3.17 (95%CI: 2.67-3.77) throughout infancy.

**Conclusion:**

LBW infants continue to be at increased risk of mortality throughout the first year of life. Efforts to reduce mortality in this high-risk group should focus on the entire infant period.

# Sub article: Characterization of Newborn Mortality in Muranga District, Central Kenya, 2010

**Published:** 2015-07-22

**Authors:** Jane Githuku

**Affiliations:** 1 Kenya Field Epidemiology and Laboratory and Training Program

## Abstract

**Background:**

In Kenya, neonatal mortality accounts for 60% of the infant deaths with >42,000 neonatal deaths reported annually. Data collected routinely on newborn deaths, through the health information system is aggregated and does not characterize the causes of deaths. We therefore aimed to describe these deaths in Muranga District in Central Kenya.

**Methods:**

We reviewed surveillance data from the District heath information system on neonatal and perinatal deaths from January 2010 to December 2010. A newborn death was defined as death occurring after 28 weeks of gestation and within the first 28 days of life. We obtained information on demographics, birth weight, mode of delivery, causes of death and parity. Data was analyzed using- Epi-Info version 3.5.3.

**Results:**

We obtained 272 recorded newborn deaths of which 149 (54.8%) were live births and 123(45.2%) were still births (macerated = 66; 24.3%; fresh =57; 21%.). Causes of death among the live births were prematurity 57(38.2%); birth asphyxia 44(29.5%); neonatal sepsis 25 (16.8%) and congenital anomalies 21(14.1%). Eighty six (57.7%) of the live babies died within the first 24 hours of birth and 140 (94%) within seven days of life. Mean birth weight was 2118 grams (SD; 905.3g) and 152 (57.4% n = 264) had a birth weight of <2500g. Most (218; (80%) deliveries were vaginal with 32 (14%) having breech presentation. There were 15 (5%) home deliveries with 159 (61.6%) of the mothers being in their first or second pregnancy.

**Conclusion:**

Prematurity is the leading cause of death among newborns in Muranga District. Almost half of the deaths were stillbirths. Health facilities should be well equipped to provide specialized care for the newborns. The high proportion of still births indicates a need to improve prenatal diagnosis of pregnancy complications and intra-partum management of labor.

# Sub article: Review of Maternal Mortality in Central Province, Kenya, 2009-2010

**Published:** 2015-07-22

**Authors:** Onesmus Muchemi

**Affiliations:** 1 Kenya Field Epidemiology and Laboratory and Training Program

## Abstract

**Background:**

Maternal mortality ratio for Kenya was estimated at 488/100,000 live births in 2009. The recent maternal mortality ratios for provinces are unknown. We therefore retrospectively reviewed data to estimate the hospital based maternal mortality ratio and describe the possible causes of the deaths in Central province of Kenya in 2009-2010.

**Methods:**

We retrospectively reviewed maternal deaths between1st July2009 and 30th June 2010. Data was abstracted with a standard form, entered and analyzed using Ms. Excel. The variables extracted included;age, parity, level of facility, timing of death, mother's status on admission, antenatal attendance, mode of delivery, cause of death and newborn's outcome.

**Results:**

Of 111 deaths reported only the audited 73 were analyzed. The maternal mortality ratio was 124/100,000 live births. The mean age was 31years ±6. Thirty-four (47%) deaths occurred in age group 30-39 years; 31(42%) among 20-29 year olds. Forty-six (63%) women died of hemorrhage and 10 (14%) died of eclampsia. Forty-five (61%) women were admitted in stable condition while 51(71%) died within 24 hours after admission. Sixty-six (90%) women died in government facilities; 53(73%) in level four, 18(25%) in level five and 2(3%) at level three. Sixteen (22%) were primigravida and 16 (22%) had previous abortion. Forty two (58%) women had attended two or more antenatal clinic visits, 28 (39%) delivered through caesarian section, 24 (33%) skilled vaginal delivery, 23 (31%) unskilled delivery and12 (17%) died ante-partum. Live births were 48 (66%), 19 (26%) were still births and seven (9%) were undelivered fetus.

**Conclusion:**

Maternal mortality was lower than the national average. Bleeding and eclampsia were the main causes of death. Majority of women were admitted stable. Management of complication of pregnancy should be reinforced among clinicians and emergency obstetrics care should be available in peripheral facilities to reduce the referral and labour duration. Evaluation of risks to mothers within health facilities during labor is required.

# Sub article: Assessing the Leading Causes of Mortality in less than Five-year old Children in Migori District, Kenya, 2011

**Published:** 2015-07-22

**Authors:** Elizabeth Mgamb

**Affiliations:** 1 Kenya Field Epidemiology and Laboratory and Training Program

## Abstract

**Background:**

Infant and child mortality remain high in developing countries where 12.9 million children die yearly. Around 70% of these deaths are due to conditions that could be prevented or treated with access to simple, affordable interventions. In Kenya, 33% of children who die before their fifth birthday come from Nyanza Province. We therefore sought to determine the leading cause of mortality in children less than five years in Migori District of Nyanza province.

**Methods:**

We retrospectively reviewed data of under five years old children who died in the year 2011and 2012 in the facilities offering in patient services in Migori District. Information collected included demographic Characteristic, date of death, duration of hospitalization, presenting complaints and diagnosis. Data was analyzed using MS excel.

**Results:**

A total of 364 health records were reviewed from nine facilities; 35(10%) were neonates, 151 (51%) were infants and 178 (49%) were above one year. Females were 51%. Two hundred and sixty three (72%) deaths occurred within 24 hours of admission. Private facilities and faith based health centres contributed to highest number (295; 81%) of deaths. In 187(51%) of the deaths, blood smear for Malaria was done and 109 (58%) were positive. Malaria was the commonest diagnosis (234; 65%) before death followed by anaemia (183; 50%), pneumonia (71; 20%), gastro enteritis (41; 11%), malnutrition (36; 10%), HIV (19; 5.2%). Fourteen (40%) neonates had prematurity, 8(23%) had neonatal sepsis, and 7 (20%) had respiratory distress syndrome.

**Conclusion:**

Malaria and prematurity are the leading cause of mortality in under five children and neonates respectively. Most deaths occurred in the private and faith based health facilities and within 24 hours of admission. Mothers need to be educated on importance of bringing the children early to health facilities. Strategies to strengthen private and faith based health facilities to provide quality health services and adequate care for premature neonates will help in reducing mortality in fewer than five year olds.

# Sub article: Analysis of stillbirths in Jibril Mai-Gwari I Memorial Hospital Birnin Gwari, Kaduna State

**Published:** 2015-07-22

**Authors:** Abbott Sam

**Affiliations:** 1 Nigeria Field Epidemiology and Laboratory and Training Program

## Abstract

**Background:**

In Nigeria, perinatal mortality remains a major health problem with the balance heavily tilted towards the stillbirth component. Knowledge of the relative importance of the different causes of stillbirth is lacking, even in hospital settings. We conducted the study to determine the stillbirth rate and the associated risk factors.

**Methods:**

We review records of delivery at Jibril Mai-Gwari Memorial Hospital Birnin Gwari in 2009. Information on mothers was collected from delivery register. We develop a checklist to assess staff strength and equipment for delivering emergency obstetric care services. Data was analyzed at univariate and bivariate levels and was presented as means, proportion and odds ratio with corresponding 95% confidence interval.

**Results:**

Of the 964 women who delivered 243 (28%) were teenagers, 539 (57%) were booked for antenatal care (ANC), 22 (4.1%) had complications during ANC period, 77 (8.1%) had labour and delivery related complications. Stillbirth rate was 117 per 1000 births with 73.9% and 26.1% being fresh and macerated stillbirths respectively. Antepartum haemorrhage, prolonged/obstructed labour and ruptured uterus were the three leading cause of stillbirth. The main risk factors for Stillbirths are grandmultiparity (OR = 3.6, 95% CI = 1.9, 7.0), teenage mothers (OR 2.79, 95% C I 1.72, 4.50) and no antenatal care (OR = 3.8, 95% CI = 2.4, 6.0).

**Conclusion:**

The stillbirth rate is very high and strategies to reduce it should focus on quality antenatal and intra-partum emergency obstetric care and early referrals.

# Sub article: Pneumonia in children under five in Uganda: symptom recognition and actions taken by caretakers

**Published:** 2015-07-22

**Authors:** Doreen Tuhebwe

**Affiliations:** 1 Uganda Field Epidemiology and Training Program

## Abstract

**Background:**

Pneumonia is a leading cause of death in children under five years of age and this constrains the attainment of the millennium development Goal four. Such deaths could be averted if caretakers recognized danger symptoms and signs of pneumonia, sought appropriate treatment promptly. We assessed caretaker's knowledge and recognition of pneumonia danger signs in children under five and the actions taken in the management of pneumonia in Mukono District, Uganda.

**Methods:**

We conducted a cross sectional study in Mukono north health sub District between July and August 2012. A total of 278 caretakers, whose children had suffered from probable pneumonia defined as cough with fast breathing two weeks prior to the study were interviewed using a structured questionnaire. We assessed knowledge, symptom recognition and identification of pneumonia danger signs and the actions taken in the management of pneumonia using descriptive and inferential statistics. Seven in-depth interviews with caretakers and three focus group discussion with community health workers (CHWs) were conducted.

**Results:**

Majority 96.4% (267/I278) of the caretakers were female, and 85% (228/268) were biological mothers. Only 41 (14.7%) of the caretakers mentioned three or more danger signs, nearly a half (45.3%) mentioned only two while 40.0% mentioned one or none of the danger signs of pneumonia. The most commonly stated pneumonia specific danger sign was chest wall in drawing. Other general signs were: severe weakness (65.11%) and inability to feed (37.8%). In terms of action taken, most caretakers offered drinks (49.6%) and traditional herbs (45.3%), followed by seeking care at a health facility. Although a third (31.65%) gave antibiotics, a significant number of caretakers still believe in the use of traditional medicine. Caretakers who could only mention one or no danger sign were less likely to have visited a health facility (OR = 0.23, 95% CI; 0.11-0.48).

**Conclusion:**

Knowledge about pneumonia danger signs among the caretakers was low. Caretakers used both conventional and traditional forms of treatment to manage pneumonia. More emphasis should put on the use of trained heath workers for the management of childhood pneumonia. Community health workers should be supported to manage pneumonia in communities.

# Sub article: Determinants of Immunization coverage in children 12-23 months: A further analysis using the 2011 Ethiopian Demographic and Health Survey

**Published:** 2015-07-22

**Authors:** Yihunie Lakew

**Affiliations:** 1 Ethiopia Field Epidemiology and Training Program

## Abstract

**Background:**

Immunization remains one of the most important public health interventions to reduce child morbidity and mortality. Full immunization coverage in Ethiopia is low among children aged 12-23 months, which shows many children in Ethiopia do not get the benefits of immunization. Determinant factors for low immunization services use are poorly understood. The objective was to identify factors associated with immunization coverage and visualize the spatial gaps in vaccination coverage in Ethiopia among children between 12-23 months.

**Methods:**

This study uses the most recent Ethiopian Demographic and Health Survey of 2011. The survey was cross sectional by design and used multistage cluster sampling procedure. A total of 1927 children in 12-23 months of age were extracted from kids’ dataset for this analysis. Mothers self-reported and observations of vaccination cards were used to determine vaccine coverage and to identify possible independent predictors. The Getis-Ord G-statistic was used to show the special distributions and gaps in vaccine coverage at regional, zonal and cluster level.

**Results:**

The prevalence of fully immunize children was 24.3%. Specific antigens’ coverage ranges from 36.5% (DPT), 44.3% (Polio), and 55.7% (Measles) and to 66.3% (BCG). ANC attendance [AOR 95%CI; 2.4(1.86-3.09)], institutional delivery [AOR95%CI; 1.8(1.28-2.56)], women's information on community conversation program [AOR95%CI; 1.7(1.36-2.22)] and women in rich group [AOR 95CI; 1.6 (1.23-2.18)] have positive effect for full vaccination coverage. Muslim followers [AOR95%CI; 0.6 (0.47-0.79)] and educated partners [AOR95%CI; 0.77 (0.60-0.99)] have a reduction effect for full vaccine coverage. Almost similar findings were observed for specific antigens. However, variables like heard TB [AOR 95%CI; 2.4 (1.77-3.38)] and higher birth order [AOR 95%CI; 0.55 (0.33-0.90)] have, respectively, positive and reduction effect for BCG vaccine. Child death [AOR 95%CI; 1.3 (1.00-1.67)] and paid job [AOR 95%CI; 1.3 (1.03-1.55)] appeared as a positive effect as well rural residence [AOR 95%CI; 0.61 (0.41-0.92)] emerged as reduction effect for Measles vaccination. Maternal age in 25-34 group [AOR 95%CI; 1.5 (1.17-1.95)] and paid job [AOR 95%CI; 1.3 (1.04-1.58)] found to be a positive effect for DPT vaccination. Paid job [AOR 95%CI; 1.3 (1.08-1.61)], women in 35-45 age [AOR 95%CI; 1.9 (1.26-2.78)] appeared as a positive effect for polio vaccine. Rural residence [AOR 95%CI; 0.58 (0.43-0.79)] also have a reduction effect for polio vaccination.

**Conclusion:**

Maternal health service use including ANC and institutional delivery, awareness on community conversation program, awareness on TB, young maternal age, paid job and child death experience have positive effect for vaccination. Whereas religion, educated partner, residence and higher birth order have reduction effect for vaccination coverage. Further efforts are needed on maternal health services to speed up the immunization coverage in the country. Furthermore, focus has to be given in remote rural areas, husbands and Muslim communities.

# Sub article: Baseline data for a Field evaluation of Fluorescein diacetate (FDA) vital staining for detection of viable Mycobacterium Tuberculosis and assessment of culture performance during treatment follow up

**Published:** 2015-07-22

**Authors:** Claude Muvunyi

**Affiliations:** 1 Rwanda Field Epidemiology and Laboratory and Training Program

## Abstract

**Background:**

Identification of acid-fast bacilli (AFB) in the sputum smear during Tuberculosis therapy is in some settings considered a predictor of patient infectivity and treatment failure. However, the meaning of positive smears at follow-up examination is not always clear. Smear microscopy cannot distinguish between AFB that is alive or dead. There is a strong interest for a rapid and simple method able to elucidate the real value of a positive AFB smear during treatment follow-up.

**Methods:**

The study was conducted at national reference Laboratory in Rwanda to evaluate baseline data and to prepare the launching of an operational study to evaluate the FDA vital staining. We retrospectively evaluated a cohort of 205 adult patients (142 men and 63 women) diagnosed with new case pulmonary Tuberculosis.

**Results:**

Of the 205 patients, delayed sputum smear and culture conversion occurred in 91 (44%) and 47 (23%) patient beyond 2 m0nths, respectively. Sputum smears remained positive for AFB beyond 5 months of therapy in 13 patients (6%). Of the 13 patients with positive smears after 5 months, 10 (77%) converted to negative culture results before negative smear results, and 3 (23%) had persistently positive culture results. Thus, in this study population, there were only three true treatment failures beyond 5 months.

**Conclusion:**

Persistent sputum smears positive for AFB at the end of treatment do not necessarily indicate treatment failure but is more likely to be associated with negative culture results due to the presence of nonviable mycobacteria. Based on the baseline data presented here, we proposed to investigate the feasibility of implementing the FDA vital staining in Rwanda where microscopy remains the only one tool widely accessible for TB treatment monitoring.

# Sub article: Evaluation of key indicators of Malaria using guidelines from the World Health Organization, Mozambique, 2011

**Published:** 2015-07-22

**Authors:** Cristolde Salomao

**Affiliations:** 1 Mozambique Field Epidemiology and Laboratory and Training Program

## Abstract

**Background:**

In Mozambique, Malaria is an important cause of morbidity and mortality and accounts for 45% of all outpatient visits, 56% of hospitalizations in children, and almost 26% of hospital deaths. Our goal was to evaluate key indicators of Malaria surveillance and control using guidelines from the World Health Organization (WHO).

**Methods:**

A descriptive study was performed, from July to December 2011. The convenience sample consisted of 22 health units in all provinces of the country, two health facilities in each, one with laboratory capacity (microscopy) and another without. To understand the practices and attitudes of the staff responsible for registration, diagnosis and treatment of Malaria cases at health facilities, primary data were collected using a questionnaire and secondary data were collected by reviewing the log books of outpatient and prenatal visits, and laboratory and pharmacy. The evaluation of the indicators was performed following WHO guidelines for countries with moderate or high levels of Malaria transmission.

**Results:**

A total of 66 health care workers involved in the diagnosis and treatment of Malaria were surveyed. A total of 95.5% (63/66) reported knowing the criteria for treatment with anti-malarial drugs. With respect to Malaria case reporting, 42.4% (28/66) reported all cases of febrile syndrome, 40.9% (27/66) reported rapid test confirmed Malaria, 10.6% (7/66) reported clinically diagnosed Malaria and 6.1% (4/66) only reported cases treated with anti-Malarial drugs. The information on Malaria cases was registered on 18 different instruments. Around 61.730 suspected Malaria cases were tested, 42.7% (26.369/61.730) were confirmed Malaria; 57,490 cases of suspected Malaria were registered. Among pregnant women attending antenatal clinics, 32.2% (7.581/23.524) had a second dose of Intermittent Preventive Therapy (IPT), and 69.9% (16.436/23.524) received bed nets. During mass distribution of mosquito nets, greater than 95% of households received nets.

**Conclusion:**

There is no standardization in the registration of suspected Malaria cases. The results of the tests for the diagnosis of Malaria are not used to direct treatment. The proportion of pregnant women in antenatal clinics receiving a second dose of IPT and a bed net were low. Coverage of mosquito nets for mass distribution was good.

# Sub article: Investigation of Febrile Syndrome suspected of Typhoid fever in Lichinga, Niassa Province, Mozambique, 2012

**Published:** 2015-07-22

**Authors:** Fernando Padama

**Affiliations:** 1 Mozambique Field Epidemiology and Laboratory and Training Program

## Abstract

**Background:**

In the first semester of 2012, the provincial health department in Niassa reported that approximately 50 patients, mostly children >5 years, were seen at the provincial hospital in Lichinga with febrile syndrome. Reported symptoms included fever, malaise, abdominal pain, and constipation. The presumptive diagnosis was typhoid fever, but no patients were laboratory tested. A team from FELTP was sent to investigate with objectives to describe the basic epidemiology of the disease, determine the cause, identify risk factors for illness, and provide recommendations for prevention and control.

**Methods:**

Medical records were reviewed for patients hospitalized at the provincial hospital, between 2011 and 2012, to identify individuals meeting the case definition of suspect typhoid fever. Blood and stool samples were collected from 5 symptomatic hospitalized patients and 1 symptomatic contact. Currently hospitalized patients meeting the case definition were interviewed using a standard questionnaire to identify risk factors.

**Results:**

In 2011, 37 cases were identified. In 2012, 74 cases were identified, occurring sporadically between January and May, with 2 deaths (2.7%). Among cases, 54 (74%) were male, the median age was 11 years, with age ranging between 1 and 30 years. The majority of cases resided in 3 neighborhoods of Lichinga. The presence of Salmonella spp was lab confirmed in 3 of the 6 blood samples tested. Due to lack of lab reagents, it was not possible to identify to species of Salmonella. All 6 suspect cases interviewed consumed water from open household wells, and no common source of infection was identified.

**Conclusion:**

The results indicate an increase in patients with clinical syndrome suggestive of typhoid fever. Recommendations made by the investigation team included implementation of active surveillance, guidance to health care providers regarding additional case reporting, reinforcements of local laboratory capacity to support lab diagnosis of typhoid fever, and adoption of prevention and control measures.

# Sub article: Investigation of Hysteria Outbreak among School girls in Kombolcha Town, Northern Ethiopia

**Published:** 2015-07-22

**Authors:** Mastewal Worku

**Affiliations:** 1 Ethiopia Field Epidemiology and Training Program

## Abstract

**Background:**

Hysteria had been known for more than 600 years in a variety of cultures and settings for significant adverse of public health consequences and economic implications. The objective of this study was to conduct outbreak investigation of hysteria and identify its determinants to implement public health interventions

**Methods:**

We defined case as any person within a teenager age group exhibiting fainting, hyperventilation, crying loudly, speaking gibberish, headache or a trance-like state or seizure-like movements with in a sudden onset between 26 December 2012 and 15 January 2013. We investigated all cases in the affected schools using line lists and questionnaires and we compared 50 cases to100 controls matched by sex and age. Detailed discussions were also undertaken with concerned body and we conducted environmental survey in affected schools. SPSS-Version 20 multivariate backward conditional logistic regression model was utilized for data analysis.

**Results:**

From 26 December 2012 to 15 January 2013 we identified of 50 cases and no death, mean age 13 years (ranges 9-16years) with SD 1.5. Children aged > 15 years were highly affected (8.8%). A total attack rate of 3.3%. The affected students were girls, and mostly friends. The predominant clinical symptoms were headache, shout and crying, fainting and nausea. No related factor for environmental finding was occurred. The phenomenon started in one of the classrooms of the 8th standard girls and later progressed to grade four. The perception of evil devil force with hysteria cases (Adjusted Odds Ratio (AOR) 5.3, (95% Confidence Interval (CI) 2.3-12), psycho stress (AOR 2.6, (95% CI 1.14-5.72), and seeing the affected students (AOR 2.9; 95% CI 1.1-7.78) were independent risk factors of disease contraction and knowledge of hysteria transmission (AOR 0.21, 95% CI 0.09-0.49) and separation of girls from the school environment at least for 1-2 weeks (AOR 0.22, 95% CI 0.1-0.5) were found to have protective effect.

**Conclusion:**

We identified a hysteria outbreak in primary school girls. Threatening situation associated with a socio-cultural belief with psycho stress and it facilitated by seeing friends’ problem and hearing sound. Mostly affected students were teenager age groups. Characteristics of Epi curve was propagated epidemic type. Education on psychiatry illness and different risk factors related to hysteria illness should be given at school and psychiatric guidance should be available to manage cases.

# Sub article: Evaluation of a Malaria Rapid Diagnostic Test among Febrile Children in Nasarawa, North Central, Nigeria

**Published:** 2015-07-22

**Authors:** Ishaku Aklaya

**Affiliations:** 1 Nigeria Field Epidemiology and Laboratory and Training Program

## Abstract

**Background:**

Malaria rapid diagnostic test (RDT) is an antigen capture assay that enables rapid diagnosis of Malaria without the need for electricity or highly skilled technicians. Though potentially useful, its adoption needs to be guided by local test sensitivity. We carried out the study to evaluate the diagnostic performance of a commercially available RDT (Malaria Pf rapid device, Biotech Laboratories Limited, United Kingdom) among febrile children in Nasarawa State, Nigeria.

**Methods:**

This was a prospective observational study involving 400 children (aged 6 months to 12 years) who presented to the Pediatrics Outpatient Department (POPD) of Dalhatu Araf Specialist Hospital, Lafia, with fever between March and October, 2009. Finger prick blood samples were collected from each of the patient (day 0) and immediately tested for falciparum Malaria by both Giemsa microscopy and rapid diagnostic test (RDT). Patients with positive RDT and microscopy on day 0 were simultaneously retested on day 7 (after antimalarial therapy) by both diagnostic methods.

**Results:**

The prevalence of Malaria among the study cohort was 40.8% by microscopy and 39.5% by RDT. The RDT had a sensitivity of 90.2% and specificity of 95.4%; with positive and negative predictive values of 93.0% and 93.4%, respectively. Test accuracy was 93.3%, whereas reliability was 85.3%. Test sensitivity is reduced by low parasite density (100% at > 1600/µl versus 69.2% at <800/µl). Of the 69 patients who were retested on day 7 after antimalarial treatment, 18 (26.1%) still had positive RDT test even though negative by microscopy and afebrile at the time of follow up.

**Conclusion:**

The diagnostic performance of the RDT in this study is good. Hence, it is recommended as an alternative method for diagnosis of Malaria, especially when microscopy is not feasible

# Sub article: Burden of Schistosomiasis: Findings from the Neglected Tropical Diseases Routine Surveillance, Rwanda 2012

**Published:** 2015-07-22

**Authors:** Irenee Umulisa

**Affiliations:** 1 Rwanda Field Epidemiology and Laboratory and Training Program

## Abstract

**Background:**

Neglected Tropical Diseases (NTDs) are the most common diseases affecting the poorest 500 million people living in sub-Saharan Africa. In Rwanda, NTDs Control Program specifically targeting schistosomiasis, soil transmitted helminths, lymphatic filariasis, onchocerciasis and trachoma was initiated in 2007. Surveillance for NTDs is conducted through Rwanda's Health Management Information System (Rwanda HMIS), a web-based platform built on the District Health Information System open source software. This study sought to determine the trends of reported schistosomiasis in Rwanda in 2012.

**Methods:**

The diagnosis of schistosomiasis cases is done through laboratory confirmation of stool samples through wet mount technique. Secondary data analysis of HMIS (2012) data was done focusing on schistosomiasis. The national (2007/8 and 2009/11) mapping results, the 2009/10 impact studies and the mass drug administration reports from 2009 - 2012 were also reviewed.

**Results:**

Total number of reported cases through routine disease reporting by health centers for the period of January – December 2012 was 450. Schistosomiasis cases were registered in 29 out of 30 districts (97%). The majority of cases were reported in 40% of the districts with wetlands, representing 63% of the total cases. The same districts have been targeted for mass drug administration of praziquantel which has been organized once a year since 2008, with a coverage rate between 83 and 93%. Cases were evenly distributed throughout the year with no seasonal variation observed.

**Conclusion:**

The 2012 data shows that schistosomiasis remains a public health challenge in Rwanda. Although most cases occur in the high risk districts, over a third of them are reported from the districts that are considered low risk. Additional mapping and targeted interventions are required to strengthen the prevention and control measures for schistosomiasis.

# Sub article: Factors associated with long acting Reversible Contraceptive use among women of Reproductive age in Lubaga Division, Kampala District, Uganda

**Published:** 2015-07-22

**Authors:** Ronald Anguzu

**Affiliations:** 1 Uganda Field Epidemiology and Training Program

## Abstract

**Background:**

Uganda's high total fertility rate, unwanted pregnancies, unsafe induced abortions and high maternal mortality rate is a public health concern and may be attributed to low contraceptive use. Increased use of long-acting reversible contraceptives (LARC) has been proposed as a strategy to reverse undesirable maternal health consequences. We conducted a survey to determine the factors associated with LARC use among women of reproductive age in Lubaga division, Kampala District.

**Methods:**

A health facility-based, cross-sectional study was conducted from March and April 2012 among women (15 - 49 years) attending reproductive health services at private and public health facilities in Lubaga division, Kampala District. We defined LARC as contraceptives administered less than once per cycle. Using modified Kish Leslie formula, 565 respondents sampled were interviewed using pre-tested, semi-structured questionnaires to measure factors associated with LARC use. Probability proportional-to-size sampling was used to sample 20 health facilities; 18 private clinics were randomly selected and 2 public health facilities purposively selected. Systematic sampling used to identify participants for inclusion. The outcome variable was current LARC use, statistical analysis done using SPSS- 17.0 and variables with p < 0.10 were included in the multivariable logistic regression model.

**Results:**

Among 565 participants, mean age (SD) - 26.34(5.35), current LARC use was 31.8% and those with high knowledge of LARC had 3.6 times increased odds of currently using LARC (AOR = 3.61, 95%CI 2.12-6.17). Respondents who previously used LARC had 5.75 times increased odds of currently using LARC currently than those who had never used LARC (AOR = 5.75, 95% CI 3.67-9.02). Attitude predictors of current LARC use were agreeing that; LARC should be used by married women (adj. OR 0.48 95%CI 0.27, 0.83), male partners should decide their female choice of contraceptive (adj. OR 2.65 95%CI 1.72, 4.07) and health workers should explain the contraceptive side effects (adj. OR 2.86 95%CI 1.15,7.17)

**Conclusion:**

Strategies to be included within family planning programs by Lubaga division urban council should include; dissemination contraceptive information about various contraceptive methods to reproductive aged women and through increased focus on addressing attitudinal barriers to LARC use should be conducted.

# Sub article: Determinants of Male Partner Attendance of Routine Child Immunization Care in Hoima District, Uganda, July 2012

**Published:** 2015-07-22

**Authors:** Charles Baguma

**Affiliations:** 1 Uganda Field Epidemiology and Training Program

## Abstract

**Background:**

Child survival is dependent on numerous factors including increased utilization and timely completion of routine child immunization (RCI) schedule for optimal immune response to the vaccines and early disease protection. However, utilization and timely completion of RCI is suboptimal in Uganda and sub- Saharan Africa at large. Numerous studies have shown that male partner attendance of RCI services improves women's immunization behavior, increases utilization and timely completion of an infant's routine immunization schedule in low resource settings. This study investigated the level, perceived benefits and predictors of male partner attendance of RCI care.

**Methods:**

This cross-sectional survey used multistage cluster sampling to obtain 10 parishes of Bugahya Health sub- in which 300 adult men with children aged 12-23 months prior to the survey were selected. A structured questionnaire was used to elicit responses about male attendance of routine child immunization at least once as the main outcome variable. A Generalized Linear Model (GLM) was used for analysis in STATA version 10.0 to obtain Prevalence Risk Ratios (PRR) for association between the binary outcome and independent factors.

**Results:**

Overall, 38% (95%CI: 1.57-1.68) men attended at least one of their child's RCI session. Respondent's mean age was 35 years (standard deviation 10). Perceived benefits of male attendance of RCI sessions were; child care education (56%), sharing child care responsibility (54%) and knowledge of next visit (39%). Factors independently associated with high male attendance of RCI were: knowledge of RCI schedule (adj.PRR = 1.97, 95%CI 1.51-2.66), and knowledge of 4 or more vaccine preventable diseases (adj.PRR = 1.5, 95%CI 1.15-2.04). However, factors for low attendance were: perceiving RCI as a woman's role (adj.PRR = 0.81, 95%CI 0.73- 0.95), long waiting time at immunization clinic (adj.PRR 0.52; 95%CI 0.39-0.68) and competing work demands (adj.PRR 0.06; 95%CI 0.02-0.14).

**Conclusion:**

The low level of male attendance of RCI is a signal to continued untimely routine child immunization in Uganda. Empowering men with knowledge of RCI schedule and of RCI as a joint parental preventive health care responsibility coupled with quick routine immunization sessions at clinics are necessary to improve male attendance of RCI care.

# Sub article: Prevalence of Sexual Coercion and its association with Unwanted Pregnancies among Pregnant Females aged 15-24 Years in Urban Antenatal Clinics, Kampala District, Uganda

**Published:** 2015-07-22

**Authors:** Suzan Tusiime

**Affiliations:** 1 Uganda Field Epidemiology and Training Program

## Abstract

**Background:**

Sexual coercion is associated with sexually transmitted infections and unwanted pregnancies with consensual unsafe abortions, and increased maternal morbidity and mortality. Current literature focuses mainly on its risk factors but less on its resultant deleterious health effects. We conducted a cross-sectional study to determine the prevalence of sexual coercion and its association with unwanted pregnancies among young pregnant women 15-24 years in Kampala, Uganda.

**Methods:**

A cross-sectional study with both quantitative and qualitative methods was conducted among 416 pregnant women 15-24 years, attending antenatal clinics in Lubaga division, Kampala, from 2nd April to May 22nd 2012 using systematic sampling. Data on pregnancy history, sexual coercion and other reproductive health variables were collected by female interviewers using semi structured questionnaires. Generalized Linear Models with log rank and Poisson family were used to estimate Prevalence Risk Ratios of unwanted pregnancy and associated 95% confidence intervals using robust variance estimator. Quantitative analyses were done using STATA - 10.0 while qualitative data were analyzed using thematic content analysis.

**Results:**

Among the 416 pregnant women 32.2% (134/416) reported having ever been sexually coerced of which 52.2% (70/134) had been sexually coerced around the time of conception of the current pregnancy. The prevalence of unwanted pregnancies was 32.5% (135/416) and was higher among respondents who had ever been sexually coerced relative to their counterparts 46.3% versus 25.9% p < 0.001). Both having ever been sexually coerced and sexual coercion around the time of conception of the current pregnancy were independently significantly associated with unwanted pregnancies [Adjusted- Prevalence Risk Ratios = 1.54, 95% Confidential Interval: (1.17-2.02)] and [Adjusted- Prevalence Risk Ratios= 1.37, 95% Confidence Interval: (1.03-1.82)] respectively. Qualitative results indicated that different approaches narrated by the respondents met the definition of sexual coercion, which had resulted in unwanted pregnancies.

**Conclusion:**

Sexual coercion among young women 15-24 years in Lubaga division; Uganda was significantly associated with unwanted pregnancy. Young women who had ever been sexually coerced had higher rates of unwanted pregnancies than those who had never been sexually coerced. Comprehensive sex education and youth friendly centers may be useful in addressing sexual coercion and its negative outcomes.

# Sub article: A Comparison Of Fluoride-Oxalate and Plain (Serum Gel) Tube Time on Glucose Measurement at the Komfo Anokye Teaching Hospital, Kumasi, Ghana

**Published:** 2015-07-22

**Authors:** Esimebia Amegashies

**Affiliations:** 1 Ghana Field Epidemiology and Laboratory and Training Program

## Abstract

**Background:**

A continuing problem in the accurate measurement of glucose is the loss of glucose from specimens due to erythrocytic glycolysis during transport and processing leading to false interpretation of glucose results. The aim of this study therefore was to compare the different glucose measurement times between fluoride-oxalate and plain tubes.

**Methods:**

A total of 100 subjects were recruited from an adult population attending the Komfo Anokye Teaching Hospital. 75 subjects were from the diabetic clinic of the Komfo Anokye Teaching Hospital (KATH) and 25 subjects known to be non-diabetic were also added to act as controls. Study participants were selected using a simple random sampling method. Diabetic and non-diabetic patients who are not on any drug(s) known to interfere with glucose results were recruited for the study.6ml of venous blood was collected from each patient and 1ml each was dispensed into three separate fluoride-oxalate and plain tubes respectively. They were then centrifuged at different time intervals (immediate, after one hour and after two hours) to obtain plasma and serum glucose measurement respectively using the Glucose Oxidase Method. The data was analyzed with Graph Pad Prism version 5.0 and Microsoft Excel and expressed as mean ± standard deviation. The 95% confidence interval (p-value) was calculated.

**Results:**

1, 4 and 8 of the subjects presented with hypoglycaemia at immediate, after 1 hour and 2 hours fluoride-oxalate values while that of plain tubes were 3, 6 and 14 respectively. 58, 53 and 49 of the subjects presented with hyperglycaemia at immediate, after 1 hour and 2 hours fluoride-oxalate values while that for plain tubes were 58, 49 and 47. The decrease in glucose concentration after 1 hour and 2 hours in fluoride-oxalate tubes were 6.5% and 13% respectively while those in plain tubes were 8.9% and 16.7%.

**Conclusion:**

This study shows that though fluoride-oxalate does not completely inhibit erythrocytic glycolysis within two hours, its effect when left on the bench at different time interval does not show a significant change in test results (p < 0.05).

# Sub article: A Population Based Survey of Hypertension and Associated Risk Factors among adults in a Semi Urban District -Ghana, 2012

**Published:** 2015-07-22

**Authors:** Joseph Kwadwo-Larbi Opare

**Affiliations:** 1 Ghana Field Epidemiology and Laboratory and Training Program

## Abstract

**Background:**

Hypertension is a leading cause of morbidity, premature mortality and disability in Ghana. In the Akwapim North District (AKND) of Ghana's Eastern-Region (ER), health facilities data showed increased proportional morbidity of hypertension from 1.2% in 2006 to 6.2% in 2010. Given the paucity of community based surveys on hypertension and other non-communicable diseases in the region, we conducted a population survey to determine the prevalence of hypertension and its associated determinants. This is to enhance non-communicable-disease control in the District.

**Methods:**

Based on the minimum sample size of 450, we recruited 519 adults age ≥ 25years in a multi-stage sampling of standard census-zones in the District over a one-month period. We measured body weight, height, blood pressure (BP), waist and hip circumference, and obtained demographic and risk factor information. We classified BP ≥ 140/90 mm Hg as hypertensive. Univariate-analysis determined the prevalence of hypertension and known risk factors. Independent sample t-test was used to compare mean difference in anthropometric characteristics. Linear regression models ascertained predictors of systolic and diastolic BP levels (p <0.05).

**Results:**

Among the 519 participants, 62.8% were women. The mean age and body mass index (BMI) were 48.6 ±16.8 years and 23.2 ± 5.2 kg/m2 respectively. Prevalence of hypertension was 32.2% (167/519) with a male to female distribution of 27.5% (53/193) and 35.0% (114/326) respectively. The prevalence of alcohol use was the highest 65.6% (340/519). Age, BMI and waist circumference were predictors of diastolic BP.

**Conclusion:**

There is a high prevalence of hypertension in the AKND of Ghana. Stakeholders should structure interventions on hypertension to promote healthier-lifestyles. Following our feedback to health-workers and the local Authority, fitness clubs have been formed in public health facilities across the District.

# Sub article: A cross sectional study on Hypertension and Obesity among traders at Kaneshie market, Accra-Ghana, 2013

**Published:** 2015-07-22

**Authors:** Yaoundéeph Frimpong

**Affiliations:** 1 Ghana Field Epidemiology and Laboratory and Training Program

## Abstract

**Background:**

The number of reported new cases of hypertension in outpatient public health facilities in Ghana increased more than ten-fold from 49,087 in 1988 to 505,180 in 2007. A study conducted in Ghana showed that the overall crude prevalence of overweight and obesity is 23.4 and 14.1% respectively among adults aged 25 years and above. Anecdotal data from health facilities shows prevalence of these diseases is highest among market traders. As part of the world health day weeklong celebration, we conducted a screening exercise to determine the prevalence of hypertension and obesity among traders in Kaneshie market-Accra.

**Methods:**

We conducted a cross sectional study in Kaneshie market on 12th April, 2013. We screened 295 traders. Participation in the screening exercise was voluntary. Blood pressure reading was taken with a mercury sphygmomanometer. Hypertension was defined as having blood pressure ≥ 140/90 mm Hg or currently undergoing anti-hypertensive treatment. A weighing scale and stadiomenter were used to measure weight and height of traders respectively. Body Mass Index (BMI) was calculated as the ratio of the weight (Kg) and the square of height (Metres). BMI was divided into four categories, underweight (<18.0kg/ m2), normal (18 to 24kg/m2), overweight (25 to 29kg/m2) and obese (≥30kg/m2).

**Results:**

Overall prevalence of hypertension was found to be 25% (75/295). Prevalence of hypertension was 28% (70/250) among females and 11% (5/45) among males. Among the hypertensive group 47% (35/75) knew their status. Prevalence of obesity was found to be 37% (108/295). Prevalence of obesity among females and males was 42% (105/250) and 7% (3/45) respectively. Our study showed an association between obesity and hypertension (P < 0.05).

**Conclusion:**

The screening exercise demonstrated a high prevalence of hypertension and obesity among female traders at Kaneshie market compared to men. The minister of health urged the traders and market women to engage in regular exercise, avoid eating in-between meals and go for regular checkups. We recommended a private collaboration with the health sector to champion the campaign on hypertension and obesity.

# Sub article: Prevalence, Knowledge and attitudes Towards Second Hand Tobacco Smoke and its Prevention among Secondary School Students in Kabalore District Western Uganda

**Published:** 2015-07-22

**Authors:** Kwesiga Benon

**Affiliations:** 1 Uganda Field Epidemiology and Training Program

## Abstract

**Background:**

Introduction: Exposure to second hand smoke (SHS) leads to disease disability and death and there is no safe level of exposure to SHS. It is estimated to be responsible for more than 600,000 deaths worldwide each year. The existing smoking policies in Uganda are not effective. There's need for evidence to support 100% smoke free policies (SFPs). This study will help create demand for enforcing laws to establish 100% SFPs. Objectives: This study aimed at determining the prevalence of exposure, level of knowledge and attitudes towards second hand smoking (SHS) and its prevention among secondary school students in Kabarole District, Western Uganda.

**Methods:**

This cross-sectional study was carried out among secondary school students in Kabarole District. 2-stage Cluster sampling was used to select the schools and classes. All students in the selected classes were eligible to participate. A pre-tested, semi structured questionnaire was used. The data was analyzed using STATA version 10.

**Results:**

VII Abstract Introduction: Exposure to second hand smoke (SHS) leads to disease disability and death and there is no safe level of exposure to SHS. It is estimated to be responsible for more than 600,000 deaths worldwide each year. The existing smoking policies in Uganda are not effective. There's need for evidence to support 100% smoke free policies (SFPs). This study will help create demand for enforcing laws to establish 100% SFPs. Objectives: This study aimed at determining the prevalence of exposure, level of knowledge and attitudes towards second hand smoking (SHS) and its prevention in Kabarole District, Western Uganda. Methods: This cross-sectional study was carried out among secondary school students in Kabarole District. 2-stage Cluster sampling was used to select the schools and classes. All students in the selected classes were eligible to participate. A pre-tested, semi structured questionnaire was used. The data was analyzed using stata version 10. Results: Majority (93%) had good knowledge of SHS as well as its risks. However, high proportions (39.5% and 79.6%) of the participants were exposed to SHS from home and from elsewhere respectively. Majority (94.6%) appreciated the risk posed by SHS. 68% of the students appreciated that the existing policies were not obeyed and majority (over 80%) were in support of 100% SFPs.

**Conclusion:**

Secondary school students are generally knowledgeable about SHS and appreciate its dangers but are still highly exposed to it. Equipping them with knowledge is not enough. We need more evidence to support the need for 100% SFPs in Uganda.

# Sub article: Prevalence and factors associated with current Tobacco use among youths in Uganda: Results from the Global Youth Tobacco Survey 2011

**Published:** 2015-07-22

**Authors:** Kadobera Daniel

**Affiliations:** 1 Uganda Field Epidemiology and Training Program

## Abstract

**Background:**

Tobacco use is one of the leading preventable causes of premature death, disease and disability in the world. The Global Tobacco Surveillance System utilizes a standardized set of repeated surveys, including the Global Youth Tobacco Survey (GYTS), in order to measure the prevalence and characteristics of Tobacco use, identify risk factors, and monitor the effectiveness of interventions. This paper presents an analysis of risk factors, and discusses these findings in relation to progress against implementation of the World Health Organization (WHO) Framework Convention for Tobacco Control (FCTC) to which Uganda is signatory.

**Methods:**

Uganda conducted the 2011 GYTS in both rural and urban schools among students aged 13-15 years. This survey used a two-stage cluster sample design to produce a representative sample of students from 100 schools country wide. Univariate and multivariate logistic regression models of risk factors of current Tobacco use were generated at 5% level of significance.

**Results:**

A total of 3481 3450 students participated in the 2011 GYTS. Of these, more than 1 in 10 students in Uganda had ever smoked one or more cigarettes, 72.2% of the students thought second-hand smoke (SHS) was harmful while 19.4% of the students reported having been exposed to SHS at home in the last 30 days. From the multivariate analysis, being male, having parents or close friends who smoke, use of other smoked Tobacco products and exposure to advertising on billboards were all significantly associated with Tobacco use among youth.

**Conclusion:**

Among school going adolescents in Uganda we found that Tobacco use was as high and 17% and was closely associated with having parents and friends who smoke and the Tobacco industry advertisement. These results indicate the urgent need for Uganda which is a party to the WHO FCTC. A comprehensive Tobacco control law will lead to the development of an effective Tobacco control program that can counter the industry maneuvers.

# Sub article: Glycaemic Control among Type 2 Diabetic Patients and Associated Factors

**Published:** 2015-07-22

**Authors:** Justin Maeda

**Affiliations:** 1 Tanzania Field Epidemiology and Laboratory and Training Program

## Abstract

**Background:**

Glycaemic control denotes regulating and maintaining blood sugar levels in diabetic patient within normal ranges. World Health Organization (WHO) recommends use of Glycated Haemoglobin (HbA1c) test as an objective measure of Glycaemic control aiming at maintaining HbA1c at an optimal value of < 7.5%. Long term glycaemic control reduces later incidence of diabetic related complications. Attaining the target has been a challenge especially in developing countries. There are limited studies exploring the glycaemic control in Tanzania. We aimed at determining the magnitude of glycaemic control and its associated factors among type 2 diabetic patients attending Tanga Regional hospital, Tanzania.

**Methods:**

A hospital based cross sectional design with systematic random sampling was conducted, December 2012 to March 2013. Blood samples were collected for laboratory testing of HbA1c level. HbA1c levels were set as an outcome variable. Explanatory variables included use of drugs and duration, Diet, Self-care, and Physical activity. Bivariate and multivariate analysis was performed. Chi Square was used in comparing between proportions, significance different set at P value of <0.05.

**Results:**

A total of 224 study participants were enrolled; female constituted to 137 (61.2%). Mean age was 55.4 (SD = 12.9). Proportion of persons who did not meet optimal levels of glycaemic control was 186 (83%). Factors that were significantly associated with attaining optimal glycaemic control were frequent fruit intake OR (95% CI), 0.3 (0.1, 0.8), Overweight 0.4 (0.1, 0.9), Food insecurity 7.1 (1.3, 53.9), highly active 0.3 (0.1, 0.9), diabetic duration for more than 2 years 2.5 (1.5, 5.1), Insulin treatment 5.1 (1.7, 15), Been on Oral Hypoglycemic Agents (OHA) single drugs 0.5 (0.2, 0.9), Diet only therapy 0.2 (0.1, 0.4), satisfying self-diabetic care 0.4 (0.2, 0.9). Persistent associated factors were satisfying self-diabetic care practice OR (95% CI) 0.20 (0.07, 0.56), Dietary therapy only 0. 05 (0.01, 0.22), OHA single drug 0.19 (0.07, 0.50), and overweight patients 0.18 (0.06, 0.6).

**Conclusion:**

Proportion of unsatisfactory glycaemic control was very high among the studied population as compared to other studies. More emphasis should be made on diabetic self-care by encouraging, use regular drugs, and increase levels of physical activity to lower weights.

# Sub article: Trend and Characteristics of Fatalities associated with Road Traffic Crashes - Nigeria, 2001 – 2011

**Published:** 2015-07-22

**Authors:** Felix Mbodi

**Affiliations:** 1 Nigeria Field Epidemiology and Laboratory and Training Program

## Abstract

**Background:**

Road Traffic Crashes (RTCs) are a major and growing public health problem. Globally, Africa has the highest road traffic mortality rate of 28.3% compared to the Americas’ 16.2% per 100,000 populations. Sub-Saharan Africa accounts for over 10% of the global RTCs with South Africa and Nigeria accounting for about 50% of this figure. The Federal Road Safety Corps (FRSC) was established in Nigeria, with the mandate to prevent RTCs and associated deaths. We analyzed RTC data from FRSC to describe the temporal trend and characterize the pattern/ causes of RTCs in Nigeria.

**Methods:**

We reviewed nationwide RTC data from FRSC from 2001-2011. We calculated incidences of RTCs and deaths per 100,000 populations. We stratified the RTCs by year and outcome. Linear regression method was used to obtain the nationwide temporal trend of deaths associated with RTCs.

**Results:**

A total of 158,657 RTCs and 64,083 deaths were recorded with a Case Fatality Rate of 40.4%. Between 2007-2011, 85% of (25,209) deaths were males and 0.7% of which were male children under 10 years old. The highest number of deaths 9,946 (16.7%) was recorded in 2001 while the lowest 4,065 (6.8%) in 2010. There was a death trend decline (P < 0.05). Annual incidence of RTCs and deaths are 10.3 and 4.2 per 100,000 population. Of 5,223 causes of RTCs in 2010, 26.6% (95% Confidence Interval [CI]:25.5-27.8) were attributed to over-speeding; 16.5% (95% CI: 15.5-17.5) attributed to dangerous overtaking and 9.5% (95% CI:8.8-10.4) attributed to loss of control.

**Conclusion:**

Principal cause of RTCs in Nigeria is due to over-speeding. FRSC should be equipped with radar-guns to detect over-speeding and stringently penalize defaulters; this will further reduce the already declining death trend due to RTCs.

# Sub article: Road traffic fatalities in Gauteng Province of South Africa, 2007-2011

**Published:** 2015-07-22

**Authors:** Akhona Tshangela

**Affiliations:** 1 South Africa Field Epidemiology and Laboratory and Training Program

## Abstract

**Background:**

In South Africa road traffic injuries (RTIs) are the second leading cause of injury related fatalities after homicide/ interpersonal violence. Understanding the epidemiology of RTIs and fatalities is critical to informing research and policies to reduce this burden. This study describes the distribution of road traffic fatalities (RTFs) in Gauteng province of South Africa from 2007-2011.

**Methods:**

We conducted a retrospective descriptive analysis of RTFs data collected from Gauteng Province through the National Injury Mortality Surveillance System (NIMSS). NIMSS collects information on injury related mortality from medico- legal laboratories (mortuaries). Data from 7/10 and 9/10 of the mortuaries in Gauteng was analysed for 2007 and 2008-2011, respectively. We calculated rates and proportions, and determined trends. Population estimates for Gauteng Province (2007-2011) were used for fatality rate calculations.

**Results:**

A total of 16644 RTFs were captured. Annual RTFs decreased by 35.8% from 4379 in 2007 to 2812 in 2011 (resident fatality rates decreased from 41.9 per 100,000 population in 2007 to 22.9 per 100,000 population in 2011). Median age was 33 years (inter-quartile range: 25 to 45 years); and 79.3% were males. By road user category: 7096 (42.6%) were pedestrians; 2991(18%) passengers; 2972(17.9%) drivers; 794(4.8%) cyclists and 2791(16.8%) unspecified. There was a significant increase of RTFs during extended long weekends, Easter holidays and the beginning of December holidays. In 2007, Easter period [week 12 (incidence rate-ratios (IRR) = 1.97, p < 0.001), week 14(IRR = 2.08, p < 0.001), week 17(IRR = 2.17, p < 0.001)], long weekends [week 32 (IRR = 1.96, p < 0.001, week 38 (IRR = 1.92, p < 0.001)], December period [week 48(IRR = 2.21, p < 0.001), week 49(IRR = 2.15, p < 0.001), week 50(2.29, p < 0.001)] compared to the first week of the year which had the lowest number of fatalities. This was similar with the subsequent years. Of all the RTFs, 42.5% occurred on major roads and highways of the province.

**Conclusion:**

The findings show a decreasing trend in RTFs during the study period. A large proportion of the fatalities were pedestrians. More focused interventions should be targeted on the most affected road user groups and on the high risk periods of the year.

# Sub article: Gas inhalational injury near a smelting and pot-making industry, Kano State - Nigeria, 2013

**Published:** 2015-07-22

**Authors:** Iman Bello Wada

**Affiliations:** 1 Nigeria Field Epidemiology and Laboratory and Training Program

## Abstract

**Background:**

Acute inhalation injury could result from household and industrial gases or from bioterrorism. Symptoms range from minor respiratory discomfort through respiratory failure to death. The Federal Ministry of Health in Nigeria received a report of gas leakage in a small-scale smelting and pot-making industry in Ungogo, Kano State, on the 27th February 2013. Over a hundred victims had various degrees of injury. We investigated to identify risk factors of severe gas-inhalational injury and to advice on measures to prevent future occurrence.

**Methods:**

We interviewed health and environmental officials, victims of the gas inhalational injury, and conducted hospital records review. We traced victims to obtain additional information regarding risk factors. We documented observed practices that could pose health risks to the smelters and neighboring community. We conducted bivariate and multivariate analysis to determine risk factors of severe gas inhalational injury necessitating hospitalization.

**Results:**

We documented 158 cases, 36 (22.8%) females. The median age is 23yrs (range: 1 - 80yrs). A total of 146 cases (92.4%) were living within 50 meters from the market/pot-making industry. Multivariate analysis revealed being of female sex (AOR: 9.3; 95% CI: 2.5 - 43.6), being a smelter (AOR: 381.0; 95% CI 9.6 - 15200.9), being around the market (AOR: 22.9; 95% CI: 2.3 - 233.7) and the need for first aid (AOR: 714.3; 95% CI: 38.5 - 10000.0) as independent risk factors of severe gas related inhalational injury. None of the smelters was adhering to any standard operating procedure.

**Conclusion:**

Lack/ignorance of safety standards for small scale industries with potential to discharge harmful gaseous substances near residential areas poses significant health hazards. The long-term effect of exposure to heavy metals that could result from it among the smelters should be evaluated.

# Sub article: Knowledge and Attitudes of Bar attendants towards Secondhand Smoke and Compliance with Smoking Regulations in Bars in Kampala Central Division, Uganda

**Published:** 2015-07-22

**Authors:** Innocent Amubwine

**Affiliations:** 1 Uganda Field Epidemiology and Training Program

## Abstract

**Background:**

Smoking increases the risk of non-communicable diseases, not only for smokers but also for secondhand smokers. Secondhand smoke may adversely affect health and aggravate illness. In Uganda, 16% of the households are exposed to smoking daily. Tobacco use kills six million people every year. Its consumption is rising worldwide, with 80% increase in low- and middle-income countries. The study aims to assess the knowledge and attitude of bar attendants towards secondhand smoke and compliance with the smoking regulations in bars.

**Methods:**

The study was conducted in Kampala central division. Was cross sectional. Bar attendants were respondents for knowledge and attitudes and bars were observed for compliance. Kish Leslie formula was used to calculate the sample size (n = 286 bar attendants) and all the 261 registered bars were considered for compliance. Analysis was by determining proportions stratified by demographics characteristics for knowledge and attitudes and determining proportions for compliance.

**Results:**

Almost 75% (213/286) of the bar attendants were exposed to secondhand smoke (SHS), 92% knew SHS was harmful to their health. Majority (82.2%) were in favor of banning smoking in bars and 98%wanted non-smokers to be provided with a smoke free environment to work. Females were 96% less likely to smoke than males (OR = 0.04, 95%CI = 0.01-0.22). There was a positive association between the number of years worked and respondents’ smoking status (OR =4.0, 95% CI= 1.53-10.49). A meaningful relation was fixed between respondents’ smoking status and his/her being bothered be SHS (X2 = 36.79, P =0.000), he/she in favor that smoking should be banned in bars (X2 = 22.71, P = 0.000). The study also observed 261 bars for compliance with smoking regulations of which 44% (115/261) were located in restaurants. Only 5% (13/261) had designated smoking areas and 7% (19/261) posted a “No Smoking” sign. Full compliance was seen in 4.2% (11/261) of all the bars.

**Conclusion:**

Compliance with smoking regulation is not observed in bars and despite bar attendants’ knowledge of health effects and their attitudes toward secondhand smoke, they are still exposed to it.

# Sub article: Suspected Rabies death, Kagera region 2011

**Published:** 2015-07-22

**Authors:** Remidius Kakulu

**Affiliations:** 1 Tanzania Field Epidemiology and Laboratory and Training Program

## Abstract

**Background:**

Rabies is a viral zoonosis that causes approximately 50,000 to 100,000 deaths per year worldwide. Most deaths occur in developing countries where dogs are the major vectors. Cases of Rabies have been underestimated due to the natural history of the disease and surveillance of dog bites has remained the only accessible source of epidemiological data. In Tanzania, dog bites is a notifiable event under the Tanzania Integrated Disease Surveillance and Response strategy. The purpose of this study was to characterize the pattern of dog bites injuries reported at Kagera Regional Hospital for the year 2011.

**Methods:**

A retrospective review of dog bite records at Kagera Regional Hospital was conducted. Data were abstracted from Dog bites surveillance notification forms and analyzed in excel and Epi-Info.

**Results:**

A total of 380 cases of dog bites and 15 deaths (CFR 3.95%) were reported at Kagera Regional Hospital in 2011. The mean age of cases was 19 years (SD = 16.4), males and females were equally affected About 250 (65.8%) of dog bites incidences occurred during the morning and in 170 (44.7%) cases, the owners of the dogs could not be identified. The vaccination status of any of the dogs implicated could be verified regardless whether the owner was identified. Lower limbs were affected in 296 (77.9%) of all cases and out of all injured cases only 91 (23.9%) received ARVs due to intermittent supply of ARVs at the Regional hospital and none were found at District levels. Moreover all 380 cases were not investigated as directed by the Tanzania Integrated Disease Surveillance and Response guidelines of 2011.

**Conclusion:**

Control of dog bite injuries will require joint intervention in the region. Dog owners need to be sensitized on safe keeping of their pets to prevent harm to other people as well as proper record keeping of the vaccination status of their dogs As a result of this study Regional Hospital improved surveillance and response to dog bites injuries across the regions adding parameters such as vaccination status of the dog, follow up of unknown dogs and victims to the dog bites surveillance registers.

# Sub article: Socio-demographic and Clinical Characteristics of Diabetes Mellitus Patients attending Diabetes Clinics in Major Hospitals in Central Kenya, 2013

**Published:** 2015-07-22

**Authors:** Martin Mwangi

**Affiliations:** 1 Kenya Field Epidemiology and Laboratory and Training Program

## Abstract

**Background:**

In Kenya, diabetes is the leading non communicable disease and studies have shown prevalence ranging from 2.2% in the rural areas to 12.4% in the urban areas. There is no data available on the prevalence of complications due to diabetes. We therefore conducted this study to determine the socio-demographic, clinical characteristics, complications and co-morbidities among diabetes patient attending diabetes clinics in major hospitals in Central Kenya.

**Methods:**

We retrospectively reviewed all records of diabetes patients attending diabetes clinics in 12 major hospitals in Central Kenya between January and March 2013. Data was abstracted using standardized form and the variables included socio-demographics, type of diabetes, treatment, checkup done to assess for complications and presence of any complication and co-morbidities. Data was analyzed using Epi-Info.

**Results:**

A total of 1969 patients files were reviewed; 66% were females and 1516 (77%) were aged above 50 years with a mean of 59 years (SD ±15). Ninety two percent (1753; 92%) had type two diabetes. During the last clinic visit, 1833(93%) had random blood sugar test done and 748 (38%) had blood sugar levels of < 8.3mmol/l. Heamoglobin A1C (HBA1C) was ever done for 100 (5%) of the patients and for this group the mean HbA1c was 8.3(SD ±3.7). Foot and eye exam were done for 269 (14%) and 287 (15%) respectively. Kidney function test and micro-albumin test in urine were done for 523(27%) and 431(22%) respectively. Complications included peripheral neuropathy (411; 22%), retinopathy (259; 13%) and nephropathy (90; 5%). Sixty four percent (1260) of the patients had been diagnosed with hypertension. Insulin was used for 668 (34%) patients and 1428(73%) patients were on oral glucose lowering agents.

**Conclusion:**

Most patients are female, above fifty years, with type two diabetes and poor sugar control. HBA1C, other laboratory tests and physical exams used to assess complications were not done for most of the patients. This means that complications are not detected early and patients may present with advanced complications. We recommended refresher training for the clinicians working in diabetes clinic with focus on outcomes of poor sugar control and regular assessment for complication.

# Sub article: Epidemiological Characteristics of Patients with Obstetric Fistula in Zamfara State

**Published:** 2015-07-22

**Authors:** Muhammad Abdullahi

**Affiliations:** 1 Nigeria Field Epidemiology and Laboratory and Training Program

## Abstract

**Background:**

Obstetric fistula is highly stigmatized medico- social problem affecting mainly women of reproductive age group. In women it is an abnormal connection between urinary bladder and vagina (vesico-vaginal fistula [VVF]) and / or rectum (recto-vaginal fistula [RVF]), a severe sequelae of prolonged obstructed labour. There are about two million women living with obstetrics fistula worldwide and 400,000 to 800,000 in Nigeria with 50,000 to 80,000 new cases annually. Our objectives were to describe demographic characteristics of patients, different types and causes of obstetric fistula, so as to assist policy makers towards implementation of strategic framework for prevention of the fistula.

**Methods:**

We defined a case as any woman with uncontrolled leakage of urine or faeces as a result of condition surrounding pregnancy and child birth. We administered semi-structured questionnaire to the patients and obtained data on socio-demographic characteristics and obstetric history. Clinical data was extracted from the patients’ file. We analyzed data using SPSS version 16 and Microsoft office Excel 2007.

**Results:**

A total of 210 patients with obstetric fistula were interviewed. The median age at marriage and occurrence of fistula were 14years (inter-quartile range [IQR]: 13-16) and 17years (IQR; 15.5-20.3) respectively. The mean duration of fistula before repair was 1.5years (IQR: 0.7-2.1). 29% of the patients were divorced/ separated, literacy level was 8.6% and majority (84.7%) were rural dwellers. Of the VVF cases 3.8% attended antenatal care, about 70% delivered at home and laboured for more than 24hours. Obstetric fistula accounted for 98% of all fistulae.

**Conclusion:**

Maternal education, community outreach and sensitization on the importance of antenatal care and hospital delivery should be encouraged and negative effect of harmful traditional practices such as genital mutilation are the key issues to be addressed for effective prevention of obstetric fistula.

# Sub article: Assessment of Thyroid Dysfunction from suspected patients attending Endocrinology Department in a Tertiary Referral Hospital, Rwanda, 2010

**Published:** 2015-07-22

**Authors:** Umazimpaka Jean d'Amour

**Affiliations:** 1 Rwanda Field Epidemiology and Laboratory and Training Program

## Abstract

**Background:**

The burden of thyroid disease in general population is increasing gradually. Three main types of thyroid diseases are observed: secretion malfunction, goiter and solitary masses. Thyroid disease affects growth and may be worsen up to even cancer development and some complications may lead to death. In Rwanda, many people are seen with goiter across the community. We assessed the thyroid dysfunction status among patients attending a tertiary referral hospital in Rwanda.

**Methods:**

A cross-sectional study was conducted at Rwanda King Faysal Hospital in Endocrinology department in 2010. All patients suspected of thyroid dysfunction were enrolled in our study. Blood samples were collected and analyzed using AxSYM Ultrasensitive hTSH II (Free T3, Free T4 and Thyroid stimulating hormone test (TSH)). The Microparticle Enzyme Immunoassay (MEIA) for the quantitative determination of human thyroid stimulating hormone (hTSH) in human serum or plasma on the AxSYM system was used to assess the thyroid function status. Results were analyzed according to manufacturer's instructions to find out the status of thyroid's secretion malfunction. We defined normal ranges as 1.45 - 3.48 pg/ml, 0.71 - 1.85 ng/dl, and 0.49 - 4.67 µIU/ml for FT3, FT4 and TSH respectively.

**Results:**

Out of 140 suspected patients, 19.3% were aged between 10 to 46 years while 7.9% are from age group of 47 to 67 years. 3.6% were over 70 years old. Females were more affected than men 26.4% and 4.3% respectively giving a sex ratio male to female of 1:6. The overall secretion malfunction status was 15% while 11.4% was hypothyroidism, 3.6% was hyperthyroidism and other types of thyroid dysfunction account for 15.7%. However, one limitation of the study, thyroid function tests (TFTs) were unable to identify goiter and solitary masses which are either clinically determined or require other specialized tests.

**Conclusion:**

Screening and motoring for thyroid function is highly recommended in both sexes and ages for early detection of thyroid dysfunction. We recommend carrying out further studies with big sample size for improving early detection and intervention strategies, as well as the monitoring of thyroid disease in Rwanda.

# Sub article: Childhood Cancers in South Africa, 2000-2005

**Published:** 2015-07-22

**Authors:** Moira Beery

**Affiliations:** 1 South Africa Field Epidemiology and Laboratory and Training Program

## Abstract

**Background:**

This study describes cancer rates in South African children from 2000-2005 and compares rates to those found around the world for the sake of assessing the completeness of the cancer data available in South Africa. Childhood cancers are under diagnosed in South Africa. This is especially true for children under one year who are born at home and poor children living in rural areas far away from the nearest hospital.

**Methods:**

This was a retrospective descriptive study conducted using data collected by the South African National Cancer Registry (NCR). NCR data is population-based and includes all laboratory-confirmed new cancer diagnoses. The study analyzed all new cancers in children <15years old reported to the NCR from 2000 to 2005. Data was analyzed by year, age group, population group, cancer type, and incidence rates by year and average rate. These rates have been compared to the published rates from other countries with population-based cancer registries.

**Results:**

A total of 4,082 cases of childhood cancers were analyzed. The most common reported cancer type among children was Leukaemia (comprised of Lymphoid and Myeloid Leukaemia). Leukaemia average rate over the six-year period, was 8.81 cases per 100,000 population. The rest of the top 9 cancers were: lymphoma with an average rate of 6.5, followed by kidney cancer with 5.35; brain and central nervous system with 4.53, eye cancer with 3.16, followed by connective tissue, and bone cancers. Finishing the top ten cancers were female genital organs, skin, and primary site non-specific. Trends show that in nearly all cases rates have declined during the study period. When compared to other countries, these rates are approximately half of what is found in Australia, the United States, and Europe.

**Conclusion:**

The cancer registries of comparison countries have much greater staff and financial resources than the South African NCR. Also, and the reporting of clinical cancer diagnoses increases the completeness of the data as compared to South Africa. Patterns observed in South Africa are different than those published internationally. This may be due less to disease patterns in South Africa than insufficient reporting.

# Sub article: Prevalence of Risk Factors for Non-communicable Diseases in Rwanda, Results from a STEPS Survey, 2013

**Published:** 2015-07-22

**Authors:** Rosette Nahimana

**Affiliations:** 1 Rwanda Field Epidemiology and Laboratory and Training Program

## Abstract

**Background:**

Non-communicable diseases (NCDs) are the leading cause of death globally. They are caused by four behavioral risk factors: Tobacco use, unhealthy diet, insufficient physical activity and the harmful use of alcohol. In Rwanda, specific data for the prevalence related to NCDs and risk factors do not exist. A survey was conducted to assess the magnitude of selected behavioral risk factors for NCDs using the World Health Organization Steps wise approach.

**Methods:**

A cross-sectional population-based survey of adults aged 15-64 was conducted from November 2012 to April 2013 in the entire country. Multi-stage cluster sampling design was used to enroll a representative population for that age range. Socio-demographic and behavioral information were collected in step 1, physical measurements in step 2 and biochemical measurements in step 3. Data were analyzed using Epi-Info.

**Results:**

Overall, 7241 people who were sampled participated in the study giving a response rate of 99%. Of these, 2,692(37.4%) were males and 4549 (62.6%) were female. Among the participants, 898 (12.9%) smoked. Average age of starting smoking was 19 years. The mean number of manufactured cigarettes smoked per day was 2.5. Additionally, 2,870 (41.3%) reported drinking alcohol in the previous 30 days while 1,391 (30.6%) males and 460 (17.1%) females were engaged in heavy episodic drinking. Regarding fruit and vegetable consumption, 6,902 (99.1%) ate less than five servings of fruit and/or vegetables on average per day. Engagement in high levels of activity was reported by 3,872 (57.6.0%) while 3,672 (54.6%) were not engaged in vigorous activity. Sixteen percent were overweight, 2.7% are obese, 1,114 (16%) had a SBP ≥140 and/or DBP ≥ 90 mmHg, 4.3% had a SBP ≥160 and/or DBP ≥ 100 mmHg, 3.0% had raised fasting blood glucose and 2.9% had raised total cholesterol

**Conclusion:**

This study, the first NCD behavioral risk factor profile in Rwanda, showed substantially high prevalence of smoking, alcohol consumption, overweight and high blood pressure while consumption of fruits and vegetables was very low in the Rwandan population. These findings should inform the initiation of health promotion programs to encourage adoption of healthy lifestyles in Rwanda.

# Sub article: Low Fruit and Vegetable Consumption among Kindergarteners-Accra, Ghana

**Published:** 2015-07-22

**Authors:** Perdita Lopes

**Affiliations:** 1 Ghana Field Epidemiology and Laboratory and Training Program

## Abstract

**Background:**

Fruit and vegetable consumption (F&VC) provide important nutrients to the body and greatly reduces the risk of non-communicable diseases, especially when started from childhood. About 2.7 million lives are saved annually with sufficient F&VC. F&VC among adults in Ghana is one of the lowest worldwide, and this may also pertain to children. Since school children spend considerable time in school, what they eat during school hours is important for their development. We carried out a cross-sectional study of kindergarteners in Accra to assess F&VC and the proportion of sweetened drink consumption (SDC) among two socio-economic classes, using amount of school fees paid as proxy.

**Methods:**

Fruits, vegetables and sweetened drinks were defined prior to the study. We used multi-stage sampling to select two schools; two each from three sub-metropolitan areas from the Accra-Metropolitan area. We collected data on meals eaten by kindergartners through observation and interview, using a checklist as a guide. We calculated means, percentages, and drew graphs and comparisons using Epi-Info version 3.5.3

**Results:**

Available feeding options at school were home-packed, school-provided or meals sold by vendors. The mean age of the respondents was 4.1 years, 207/422 (49%) being males. The number who consumed school-provided and home-packed meals was 296/422(70.1%) and 272/422 (64.5%) respectively, with 146/422 (34.5%) consuming both. Only 9/422(2.2%) kindergartners consumed fruit and it was all from home-packed meals. Total vegetable consumption was 144/422 (34.1%), of which 143/144 (99.3%) was from school-provided meals. SDC was associated with social class (p < 0.05{χ^2^ =18.98}).

**Conclusion:**

F&VC is low in the study population, regardless of socio-economic class. SDC was high especially in kindergarteners from higher socio-economic class schools. Following the dissemination of our findings to the participating schools, three of the schools now encourage F&VC by making them available.

# Sub article: Evaluation of the Maternal and Neonatal Tetanus Surveillance System in Bangui, 2009-2011

**Published:** 2015-07-22

**Authors:** Ptakilnam Bossokpi-Passi

**Affiliations:** 1 Central African Field Epidemiology and Laboratory Training Program

## Abstract

**Background:**

The Central African Republic is committed to eliminating maternal and neonatal tetanus (MNT). In Bangui, despite the availability of an effective vaccine, 110 cases and 76 deaths due to MNT were notified between 2009 and 2011. Effective surveillance is essential, but the system was never evaluated. Therefore, this evaluation was conducted to see whether it meets its objectives.

**Methods:**

We reviewed 2009-2011 surveillance data and interviewed surveillance staff from 24 of 58 randomly-selected health units, as well from the eight sanitary districts, and the regional directorate. Attributes were assessed according to CDC's Updated Guidelines for the Evaluation of a Public Health Surveillance System

**Results:**

Simplicity was satisfactory with 81.8% (27/33) of staff knew the MNT case definition; 75.8% (25/33) reported to complete the form in less than 10 minutes; and 88% (29/33) knew the information circuit. Seventy-nine percent accepted MNT surveillance as part of their daily work. Completeness of the reporting forms was 79% (617/780), and 85% of forms were validated by a supervisor. Sensitivity was 46%. Only 61% of the reports were sent on time to the next level. 15% of the 24 health units conduct on-site data analysis for decision making purposes. In 2011, the system integrated sentinel surveillance of bacterial meningitis and rotavirus.

**Conclusion:**

MNT surveillance system is simple, generates quality data, is accepted by the staff involved, and can integrate other diseases without additional resources. Nevertheless, sensibility, timeliness and local analysis of data should be improved. Regular training of surveillance staff, as well as regular supervision at all levels will help to improve the MNT surveillance system in Bangui.

# Sub article: Evaluation of the Influenza Surveillance System in Ghana, 2012

**Published:** 2015-07-22

**Authors:** Joseph Asamoah Frimpong

**Affiliations:** 1 Ghana Field Epidemiology and Laboratory and Training Program

## Abstract

**Background:**

The world health organization seasonal Influenza fact sheet No.211 indicates that seasonal Influenza affects 5-10% of the world's population resulting in 250,000 to 500,000 deaths annually. Influenza epidemics are characterized by an increased morbidity and mortality in the community and increased absenteeism from work and school. Following the outbreak of Influenza A (H5N1) in 2007, where 13,371 birds died and 27,355 were culled, a surveillance system was established in Ghana. We conducted the evaluation to determine whether the objectives of the system are being met and to assess its attributes.

**Methods:**

We reviewed surveillance data for 24 sentinel sites from 2008 - 2011. We interviewed surveillance staff and other stakeholders using the centres for disease control and prevention (CDC) updated guidelines for public health surveillance system evaluation.

**Results:**

Out of 9830 suspected cases of Influenza -like illness (ILI), 1972 (20%) were Influenza virus positive and were characterized as Influenza A (84%) and Influenza B (16%). The system has few levels of reporting and case definition is easy to apply. It detected the Influenza A (H1N1) pdm09 pandemic. Case based form was modified to capture suspected cases of A (H1N1)pdm09 during the outbreak in Ghana in 2010 and 75% of the sites are optimally active. The system has a positive predictive value of 19%. Over 95% of laboratory results were sent to sites in less than a week and 93% of the forms were completely filled. Data was not analyzed at the facility, District and regional level.

**Conclusion:**

The Influenza surveillance system is complex but stable and has satisfactory data quality. There is nationwide coverage which gives a good representation of Influenza activity in the country. The system is sensitive and has the capacity to identify and characterize circulating Influenza strains in Ghana. There is fair acceptability and timeliness has improved over the years. The system is donor dependent. Our recommendations were data should be analyzed at the facility, regional and District level. The National Influenza Centre (NIC) should regularly publish and share their findings. Following our recommendation, the NIC has set aside every Friday for manuscript writing for information sharing.

# Sub article: Evaluation of the Schistosomiasis Surveillance System-Northern Region, Ghana, 2012

**Published:** 2015-07-22

**Authors:** Atasige Stephen

**Affiliations:** 1 Ghana Field Epidemiology and Laboratory and Training Program

## Abstract

**Background:**

Blood flukes of the genus Schistosoma cause Schistosomiasis. An estimated over 207 million people are infected worldwide, 90% in sub-Saharan Africa. The prevalence ranges between 54.8% - 60% in Ghana, mostly along the Volta basin. The objectives of the Schistosomiasis surveillance system are to understand the magnitude and patterns of the disease, to monitor changes in trends in different geographical locations, to inform interventions and permit assessments of control measures. A 2010 evaluation of the system from 2008-2010 in the Eastern region showed that the northern region had the lowest (0.3%) prevalence and regional rates of completeness and timeliness of reporting.

**Methods:**

The Centers for Disease Control and Prevention (CDC) guidelines for evaluating public health surveillance systems was used as reference. The point of entry was the Central Gonja District of the northern region, located along the Volta basin. Stakeholders were interviewed, observations were made and documentation was done using a checklist. We reviewed surveillance data from 2008-2012 from the national level through to the District.

**Results:**

All 10 regions reported from 2008-2012. Timeliness of monthly reporting for regions was 80%, below the target of > 90%. The northern region recorded 439 cases for the period, 34.4% were from the point of entry and a 64% (lowest) regional rate of completeness. 61.8% of cases in the region were between the months of August to December. The 5-34 age groups recorded 68% of cases. Data is not analyzed and no laboratory diagnosis is done at the District.

**Conclusion:**

Most cases are reported from settlements along the Volta basin. The region has the lowest prevalence, a possible effect of low regional timeliness and completeness of reporting. This measures the acceptability of the system in the region. The system is simple, representative, and useful with low specificity. Feedback to stakeholders after the evaluation called for an improvement in case detection, testing and acceptability of the system in the region. Seven suspected cases have been tested in the District from a previous zero. Use of community based volunteers along the Volta basin in the region will increase sensitivity and reporting.

# Sub article: Explosive Measles Outbreak in Nyanza Province, Kenya 2012

**Published:** 2015-07-22

**Authors:** Dickens Onyango

**Affiliations:** 1 Kenya Field Epidemiology and Laboratory and Training Program

## Abstract

**Background:**

Globally, Measles is a leading cause of death in children younger than five years, it is responsible for 1% of deaths annually. Although the occurrence of the disease worldwide has remarkably reduced, the disease has remained endemic in the Horn of Africa (Kenya, Ethiopia and Somalia), where persistent periodic outbreaks still occur. In 2012, an explosive Measles outbreak occurred in Nyanza Province in Kenya.

**Methods:**

We analyzed data obtained from routine case based surveillance which were laboratory investigated from January to September 2012; all cases from Nyanza Province were included. Cases with suspected Measles were interviewed by public health nurses using a standardized report form. These cases were entered into a line list for the province. A suspected case of Measles was any person presenting with fever and macula-papular rash and a cough, coryza or conjunctivitis while a confirmed case was IgM positive for Measles.

**Results:**

A total of 254 suspected cases were investigated at the Measles laboratory; 58% (n = 148) samples tested positive for Measles IgM, 39% (n = 99) tested negative and 3% (n = 7) were not tested. Among suspected cases, males constituted 51% (n = 130); the median age was 5 years (Inter-quartile range = 6) and Measles vaccine coverage was 75% (n = 190) in contrast to 83% administrative coverage. The positivity rate was 84% among those who had received two doses, 74% among the unvaccinated and 48% those vaccinated with one dose. Case fatality rate was 0.4% (n = 1). The index case was notified 3/1/2012, 24% (n = 61) cases occurred between January to March, 43% (n = 108) between April to June and 33% (n = 84). Of the 148 confirmed cases, 55% (n = 81) were males, 51% (n = 76) were aged 5 years and above whereas 18% (n = 26) were aged under one year. Seventy two percent (n = 107) had received at least one dose of Measles vaccine; 28% (n = 42) had received two doses.

**Conclusion:**

Most confirmed cases had been vaccinated. Primary vaccine failure may be responsible for this outbreak. We recommend strengthening the cold chain system to ensure children are vaccinated with potent antigens. Vaccination campaigns should also target children above age five years.

# Sub article: Prevalence, knowledge, attitudes and practices of the population on Nodding Syndrome in Pader District, Uganda

**Published:** 2015-07-22

**Authors:** Grace Mongo Bua

**Affiliations:** 1 Uganda Field Epidemiology and Training Program

## Abstract

**Background:**

Nodding syndrome (NS) is a newly emerging disease characterized by repetitive involuntary head drops towards the chest, physical and cognitive impairment in the affected children (3-15 years). These seizures are triggered by food or cold weather. Its aetiology remains unknown including treatment. NS cases have been described in South Tanzania, South Sudan and recently in Northern Uganda where statistics indicate it is occurring in epidemic proportions. Progress was made on aetiology (River Blindness) but still requires more research. The public health challenge of NS lies in the escalating cases of morbidity, mortality and disability to the population. This study assessed prevalence, knowledge, attitudes and practices of the population on NS in Pader District.

**Methods:**

Study was cross-sectional with Quantitative data collected using semi-structured questionnaires. House-to- house census using trained village health teams (VHTs) were used to identify suspect cases of NS in Pader District. Identified cases were entered into a Microsoft excel data-entry screen and frequencies run to document the descriptive epidemiology of the disease. Disease prevalence was calculated a proportion of the cases identified to the midyear district populations. Qualitative data was obtained through a focus group discussion with the caretakers at Atanga Treatment Centre and interviewing 12 Key informants. The interviews were analyzed using master sheet technique. Themes were based on specific study objectives. Data were analyzed manually using content analysis technique.

**Results:**

There were 1,978 NS cases and 107 NS deaths with a case fatality of 4.5% in Pader District. Prevalence of NS was 14.6%. There was a lot of stigma, poor care practices and low levels of knowledge. Most caretakers believed NS was caused by an evil spirit, overcrowding in IDP camps, contaminated relief food and very few from filarial worms (*Ochocerca volvulus*). A sense of fear gripped the district. Stigma existed towards patients. There was poor health seeking behavior and poor patient handling practices. Progress has been made by providing supportive care through nutritional rehabilitation and drugs.

**Conclusions:**

Ongoing Research reveals most of the perceived causes of NS are either negative or insignificant. Priority should be on funding research and supportive care.

# Sub article: Evaluation of Acute Flaccid Paralysis (AFP) Surveillance in Border Regions, Tanzania Mainland, 2009-2011

**Published:** 2015-07-22

**Authors:** Deogratius Bintabara

**Affiliations:** 1 Tanzania Field Epidemiology and Laboratory and Training Program

## Abstract

**Background:**

In 1995, Tanzania began polio eradication activities that included routine vaccination of infants, national immunization days, and active surveillance for Acute Flaccid Paralysis (AFP). The last polio case in Tanzania was identified in 1996. Tanzania has not been certified polio free since some regions have not attained the required AFP detection rate. AFP surveillance system was evaluated to determine its capacity to detect importation of wild polio virus in the border regions.

**Methods:**

CDC guidelines were used to evaluate the performance of AFP surveillance in five regions (total of 22 districts) between 2009 and 2011. We also did review of protocols, reports and data collection tools and records. The World Health Organization (WHO) sets operational targets for AFP surveillance and these were used for comparison with our findings. Data was analyzed using Epi-Info software.

**Results:**

A total of 408 AFP cases were detected between 2009 and 2011 in all the five regions, of which 54% were male and 99% were less than 15 years of age. Of all AFP cases 285 (70%) had the required number of specimens to be tested. These samples were sent to WHO accredited laboratory within 3 days and were all negative for Polio. The non-polio AFP detection rates for Dar es Salaam, Kagera, Kigoma, Mara and Rukwa were 2.0, 4.2, 3.0, 2.6 and 2.5 per 100,000 populations under 15 years respectively. The proportion of the District s in each of the regions that met the operational target of reporting at least two cases of non-polio AFP per 100,000 population under 15 years were (2/3) 66.7% for Dar es Salaam, (4/6) 66.7% for Kagera, (2/4) 50% for Kigoma, (4/5) 80% for Mara and (3/4) 75% for Rukwa. Completeness of 408 forms that were reviewed was 91.9%. Out of 22 districts, 15 were able to achieve the operational target of reporting AFP cases and 80.6% of AFP cases were notified timely.

**Conclusion:**

Training, sensitization and proper reporting should strengthened so as to achieve WHO targets for AFP surveillance within the districts so as to achieve the global certification of eradication especially in the poor performing districts.

# Sub article: Diarrhea Disease Investigation Masasi District, Mtwara, Tanzania January 2012

**Published:** 2015-07-22

**Authors:** Violasia Mushi

**Affiliations:** 1 Tanzania Field Epidemiology and Laboratory and Training Program

## Abstract

**Background:**

Diarrhea disease outbreak was reported to the Tanzania Ministry of Health and Social Welfare on 3 January 2012 by the Mtwara Regional Health Officer (RHO). The disease suspected to start after the ritual ceremony in one of the kin resides in Masasi District. Four people died on the unknown disease before seeking medical care, and was associated rituals believe. This investigation aimed to determine the magnitude, establishing the etiological agent and risk factors associated with the outbreak.

**Methods:**

An unmatched case control study was conducted and it involved active case finding. Rectal swab for laboratory investigation was collected. Controls were selected from the neighborhood. The investigation involved administering of structured questionnaire to cases and controls, house to house inspection and environmental sanitation assessment. A case was defined as any person ≥2 years of age or more with lots of watery Diarrhea from Lukuledi and Ndomoni ward from 17 December 2011.

**Results:**

Vibrio Cholera Ogawa serotype O1 was isolated. A total of 194 cumulative patients were verified in this outbreak with 2.6% case fatality rate. The age group 20-30yrs was the mostly affected. The epidemic started on 17th December 2011 and lasted for about 4 weeks. The epidemic curve showed the propagated type of an outbreak with three picks. Eating cold food (OR = 15.25), p = 0.001) was found to be associated risk factor in contracting Diarrhea disease. Scarcity of water and poor hygiene were also found to be associated with the outbreak although not statistically significant.

**Conclusion:**

We confirmed an outbreak of Cholera that was due to eating cold food as main of the risk factor Lukuledi village. Health education on food hygiene, personal hygiene and environmental sanitation were provided to community.

# Sub article: Evaluation of Laboratory-Based Multi Drug Resistant Tuberculosis Surveillance System, Tanzania, 2012

**Published:** 2015-07-22

**Authors:** Nsiande Andrew Lema

**Affiliations:** 1 Tanzania Field Epidemiology and Laboratory and Training Program

## Abstract

**Background:**

Tanzania ranks 14th among the 22 countries with the highest burden of Tuberculosis (TB) worldwide. TB cases have increased by 60% since the HIV/AIDS epidemic debut in the 1980s. The prevalence of multidrug resistant TB (MDR-TB) is 1.1%, and that of drug resistance by Mycobacterium Tuberculosis strains to any of the four first-line drugs in a new patient is 8.3%. In re-treatment patients, the prevalence of MDR-TB and of any TB drug resistance was 3.9% and 20.6% respectively. The evaluation was done to assess the system performance.

**Methods:**

Evaluation was done using CDC Guidelines for Evaluation of Surveillance Systems. Sixty forms from the cases were randomly sampled and assessed for blank response. Sample come from all over Tanzania were the MDRTB are suspected. A structured questionnaire was used to interview laboratory workers at the national level to assess the process indicators and attributes. Data was analyzed by using Epi-Info software.

**Results:**

A total of 2,527 MDR TB cases were suspected between 2008 and 2012 of which 288 (11.4%) were laboratory confirmed. Fourteen laboratory workers were interviewed. The majority 10 (71.4%) revealed that the system is simple and it is not complex in the testing algorithm. Most 9 (64.2%) of them responded that if the lab testing approach changes, it will not affect the MDRTB surveillance system. There was 95% external quality assurance concordance that meets WHO standards. Majority 82% of received specimens were in good quality. The turnaround time (TAT) of the system is 112 days in culture and drug sensitivity test by routine culture process and 60 days by using mycobacteria Growth incubation/indicator tube (MGIT) technique. The system predictive value positive (PVP) was 11.3%

**Conclusion:**

The system is simple, useful and acceptable. It is flexible with good data quality and regular internal quality control ensuring the good quality of laboratory results. The system is also stable, representative and highly sensitive with low PVP. Emphasis should be made on frequent rather than occasional use of MGIT technique as it reduces the TAT almost by half.

# Sub article: An Epidemiologic Review on the Lead Poisoning Disaster in Zamfara, Nigeria, 2010 – 2013

**Published:** 2015-07-22

**Authors:** Nasir Umar-Tsafe

**Affiliations:** 1 Nigeria Field Epidemiology and Laboratory and Training Program

## Abstract

**Background:**

The lead poisoning (LP) disaster in Zamfara State was officially reported in March 2010 and has been described as the largest in modern times by scope and magnitude. The LP incidence in Zamfara was more than 95% among under five children (U5s) from gold ore processing communities with severe outbreaks. This reduced to about 2% in 2011-2012 following interventions and about 3% in March 2013. The aim of this review is to provide an update and to summarize the relevant short and long term recommendations to effectively address the situation.

**Methods:**

We present a review of the outbreak cross-sectional, chain-referral and cluster sampling surveys conducted among U5s in some affected communities, 3 local government areas and the state wide population levels, respectively. A value of > 400 ppm is considered an elevated soil lead level (ESLL) and value of > 10 µg/dL is defined as elevated blood lead level (EBLL), confirming LP.

**Results:**

A total of 199 villages were surveyed in Zamfara, 38(19.1%) of which were confirmed to be contaminated by both ESLLs and EBLLs. Of over 5,395 U5s screened so far, more than 3,349(62.1%) are affected with various levels of lead intoxication. Up to 734 U5s were reported to have died. nterventions rendered includes environmental soil remediation conducted in only 8 of the 38 villages due to inadequate funds, chelation therapy provided to only 2,070(61.8%) LP confirmed U5s (by March 2013), and safer mining practices implemented in only 3 of 199 mining process sites (more on the way). At least 11 of the 38 villages currently require emergency remediation and chelation interventions, with an estimated 3,198(59.3%) U5s still requiring chelation treatments.

**Conclusion:**

Among the affected U5s, implementation of recommendations from these studies has caused LP-based mortality to be almost ceased. Incidence has effectively reduced from > 95% LP prevalence in 2010 to < 3% in 2013. However, despite such recorded successes the situation remains an emergency, due to lack of sufficient funding, access to low-cost intervention mechanisms, relevant action plans and political will. Over 3,000 estimated LP U5s are continuously exposed to lead intoxications in Zamfara, Nigeria.

# Sub article: Laboratory Confirmed Rotavirus Infection among Children under 5 year Hospitalized with Acute Gastroenteritis in North Eastern, Kenya, 2012

**Published:** 2015-07-22

**Authors:** Ahmed Fidhow

**Affiliations:** 1 Kenya Field Epidemiology and Laboratory and Training Program

## Abstract

**Background:**

Kenya plans to introduce rotavirus vaccines into the routine national immunization schedule. Knowledge on disease burden and circulating strains of rotavirus has been limited in many parts of the country due to lack of surveillance system and routine testing of rotavirus. As a prerequisite to vaccine introduction, we sought to know for the first time the burden, risk factors and circulating strains of rotavirus in North Eastern Kenya.

**Methods:**

We conducted a cross sectional study among children <5 years hospitalized with acute gastroenteritis at the study hospital. A semi structured questionnaire was used to collect data on socio-demographic and clinical characteristics of the study participants. Stool specimens were obtained from participants and screened for rotavirus using an ELISA technique. Further rotavirus strain characterization was performed using PAGE and RT-PCR techniques. Proportions and means were determined for categorical and numerical variables respectively. Bivariate analysis was performed to determine factors associated with rotavirus infection. Chi-square test was used for significance testing at p < 0.05.

**Results:**

Overall, 17% (n = 41) rotavirus prevalence was detected among the study participants. Independent risk factors for rotavirus infection were; underweight {OR = 14.37, CI (3.53- 58.58)}, low birth weight {OR = 5.56, CI (1.83-16.85)}, varied nutrition {OR = 5.78, CI (1.33 -25.11)} and lack of formal education among mothers {OR = 49.54, CI (4.64 -529.56)}. Long RNA electropherotypes were the predominant rotavirus RNA profiles, while G3, G9 and P4 were the predominant rotavirus genotypes.

**Conclusion:**

Rotavirus is a common aetiology of acute gastroenteritis among children <5 years. Modifiable risk factors and genotypes protectable by Rotarix and RotaTeq vaccines were responsible for rotavirus transmission in this region. We recommend nutritional support services to malnourished children in the prevention and control of rotavirus infection alongside introduction of rotavirus vaccines to children <5 years.

# Sub article: Investigation of Food Poisoning Outbreak on the Celebration Event of Circumcision in Klelen, Yogyakarta, 2012

**Published:** 2015-07-22

**Authors:** Lukman Fauzi

**Affiliations:** 1 Indonesia

## Abstract

**Background:**

On January 4, 2013, food poisoning outbreak had occurred on the celebration event of circumcision in Sleman. The investigations were conducted to obtain information about transmission source and risk factors affecting the outbreaks.

**Methods:**

The study designs were descriptive and analytic with Retrospective Cohort design. Ill subject is defined as people who consume food derived from circumcision celebration event and have clinical symptom of food poisoning. Healthy subject is defined as people who consume food derived from circumcision celebration event, but no clinical symptom of poisoning. Statistical test used was Chi-square (bivariat).

**Results:**

The number of food poisoning cases, were 133 with dominant clinical symptoms: Diarrhea (76%) and abdominal pain (45%). The shortest and longest incubation periods were 3 and 10 hours. The highest attack Rate (AR) of the food in case group was “Bacem” fried chicken (61.3%). Statistical analysis showed that risk factors related to food poisoning outbreak were consuming “Bacem” fried chicken (p-value: 0.000; RR: 3.9) and cooked rice (p-value: 0.016; RR: 0.76). Moreover, risk factors that are not related to food poisoning outbreak were consuming raw vegetable (p-value: 0.103; RR: 0.79) and spicy sauce “Sambal” (p-value: 0.697; RR: 1.04). Laboratory result indicated that there were Staphylococcus aureus bacteria in “Bacem” fried chicken. Contamination occurred because “Bacem” was stored in warm condition when distributed from seller to the celebration event organizer and the lag time between cooking process and consuming “Bacem” fried chicken was up to 28 hours.

**Conclusion:**

Food poisoning outbreak was caused by “Bacem” fried chicken which was contaminated by Staphylococcus aureus bacteria. Recommendation given to fry chicken producer were separate cooking place with cleaning place of chicken, store fried chicken in normal temperature, and distribute fried chicken as soon as cooked. To health department, always monitor and train food hygiene-sanitation to informal food producer.

# Sub article: Diarrhea Disease Outbreak in an Orphanage Associated with Multi-Drug Resistant Salmonella, Nairobi, Kenya December, 2012

**Published:** 2015-07-22

**Authors:** Nkatha Meme

**Affiliations:** 1 Kenya Field Epidemiology and Laboratory and Training Program

## Abstract

**Background:**

Diarrheal disease is the second leading cause of death in children <5 years old, resulting in 1.5 million deaths worldwide each year. In December 2012, four children from an orphanage in Nairobi were admitted to the hospital with acute Diarrhea and confirmed to have multi-drug resistant non-typhi Salmonella. We conducted an investigation to characterize the outbreak, determine risk factors, and provide recommendations to prevent further cases.

**Methods:**

We conducted a cross-sectional survey at the orphanage. A case was defined as ≥3 loose stools in 24 hours in a child <12 months old residing in the orphanage between the months of October and December 2012. We interviewed caregivers of the children using standardized questionnaires collecting epidemiological and clinical information. We also interviewed staff at the orphanage to determine if they had Diarrhea during the past three months. We collected stool samples from children and staff for culture, and infant formula and water samples for environmental analysis.

**Results:**

Of the 28 children <12months old living in the orphanage, 17 (61%) met the case definition. Among the case-patients, median agewas4 months (range: 0-9 months), and 9 (61%) were male. Ten (58%) patients were between three and six months old. All were HIV negative. Seventeen (100%) had fever, 15 (88%) had vomiting, and 14 (82%) were not feeding as usual. Fifteen (71%) patients were fed a new brand of infant formula (Odds Ratio = 6.2, 95% Confidence Interval 0.7-77). Stool specimens from two (11%) children grew non-typhi Salmonella resistant to Ampicilin, Cefoxitin, Ceftriaxone, Gentamicin, Nitrofuratoin and chloramphenicol. Four (10%) staff, which included three caregivers and one cook, reported Diarrhea in the past three months. Three (15%) specimens from staff grew E. coli. Infant feeding formula and water samples did not yield any bacterial growth.

**Conclusion:**

We confirmed an outbreak of multidrug resistant non-typhi Salmonella among infants living in an orphanage in Nairobi. This outbreak may have been associated with consumption of infant feeding formula contaminated most likely during the preparation process. The orphanage staff was educated on proper sanitation and hygiene practices, including sterile procedures for preparing infant formula.

# Sub article: Chikungunya Outbreak in Al-Hawtah District, Lahj Governorate, Yemen, 2012

**Published:** 2015-07-22

**Authors:** Maha Obadi

**Affiliations:** 1 Yemen Field Epidemiology and Training Program

## Abstract

**Background:**

Chikungunya (CHIK) is a mosquito-borne viral disease characterized by an abrupt onset of fever frequently accompanied by joint pain. An outbreak of CHIK was first confirmed in Yemen in 2010. In April 2012, FETP residents were requested by ministry of health to investigate an outbreak of unexplained fever with severe arthritis in Al-Hawtah District, Lahj governorate.

**Methods:**

CDC CHIK case definition was used: an acute onset of fever >38.5°C and severe arthralgia/ arthritis not explained by other medical conditions, within the period from 21 March-8 May 2012 in Al-Hawtah District. Data was collected on standardized investigation form covering demographic, clinical and epidemiological aspects through an active household search together with entomological surveys. Blood samples were collected at the beginning for confirming CHKI outbreak using IgM ELISA.

**Results:**

Two hundred thirty four suspected cases met the case definition. The mean age was 31 years with no significant gender difference. The index case was a 14 years old boy affected on 21 March with no history of travel or having visitors from endemic area. All cases reported fever and headache, 95% reported arthralgia and 64% joint swelling. The highest incidence rate of 10/1000 was among active age group of 25-29 years. Around half of cases concentrated in the fish market area with an attack rate 78/1000 (108/1377) which is supporting the hypotheses that infection was imported by fishermen coming from Al-Hudieda costal governorate where the first CHIK outbreak was reported. Entomological results showed high vector indices in the fish market area with house index of 50%, and container index of 28%. Samples collected for the outbreak lab confirmation found to be positive for CHIK IgM.

**Conclusion:**

This is the first CHIK confirmed outbreak in Lahj and the fourth in Yemen since 2010 that highlight the need for improving surveillance system for timely detection and prompt response. Health education and indoor fogging were implemented and successfully control the outbreak. Providing training to health personnel on early detection, reporting and proper case management together with community awareness rising and engaging in preventive efforts are recommended.

# Sub article: Evaluation of the Bacterial Meningitis Surveillance System in the Efoulan Health District - Cameroon, from 2007 to 2011

**Published:** 2015-07-22

**Authors:** Baonga Ba Pouth Simon Franky

**Affiliations:** 1 Central African Field Epidemiology and Laboratory Training Program

## Abstract

**Background:**

Bacterial meningitis, responsible for many outbreaks in Cameroon, is being surveyed together with 24 other diseases. Since its implementation in the Efoulan Health District in 2002, the system has never been evaluated. The aim of this study was to evaluate the bacterial meningitis surveillance system in the Efoulan Health District from 2007 to 2011.

**Methods:**

A descriptive cross-sectional study was conducted in 2012. Data was collected through interviews of 52 key informants, two in each facility using a structured questionnaire. Review of surveillance data was also performed using a data collection sheet. The evaluation was done according to the CDC updated guidelines for evaluating public health surveillance systems. Data was analyzed using the Epi-Info version 3.5.3 software.

**Results:**

Forty four (84.6%) key informants didn't have access to the surveillance guide and 50 (96.1%) had not been trained on surveillance in the past two years. The District witness ruptures of sample collection kits during 8 weeks and means of communication for 6 months in 2011. The case definition was mastered by 44 (84.6%) key informants, 50 (96.1%) found the notification form easy to fill and 48 (92.3%) took less than 10 minutes to fill the notification form. The proportion of health facilities weekly reports received at the District level increased from 54.1% in 2007 to 69.1% in 2011 and the proportion of reports received on time in 2011 was 18.0% at District level and 26.9% at regional level. From 2007 to 2011, the District notified 43 (44.8%) of the 96 suspected cases found in consultation registers. Cerebrospinal fluid from 32 of the notified cases were analyzed and 7 (21.8%) tested positive. No documented decision was taken by the District based on surveillance data.

**Conclusion:**

The surveillance system though simple didn't have enough resources necessary for its proper functioning. Its representativeness, reactivity, sensitivity and positive predictive value were poor. To improve the functioning of the system, we suggest training and supervision of personnel as well as supplying health facilities with sample collection kits and surveillance guide.

# Sub article: Risk factors for Contracting Anthrax in Tsholotsho District, Matabeleland North Province, Zimbabwe 2011

**Published:** 2015-07-22

**Authors:** Tafadzwa Goverwa

**Affiliations:** 1 Zimbabwe Field Epidemiology and Training Program

## Abstract

**Background:**

On 6 October 2011 Tsholotsho District notified the Provincial Medical Directorate for Matabeleland North of a suspected human cutaneous anthrax outbreak after laboratory confirmation of the disease in cattle by the Veterinary department in Tsholotsho. This study was conducted to investigate the risk factors for contracting anthrax in the affected areas of the District.

**Methods:**

A 1:2 unmatched case-control study was conducted. A case was any person in wards 2, 14 or 15, who developed itchiness of exposed skin surface, followed by a painful papular lesion then vesiculated and eventually evolved into a depressed black eschar from 28 September 2011 to 26 October 2011. A control was a resident of the same area without such a skin lesion during the same period. Data was analyzed using Epi-Info to generate frequencies, proportions, odds ratios and confidence intervals.

**Results:**

Forty cases and 80 controls were interviewed. Independent risk factors for contracting anthrax were belonging to a household where cattle deaths had occurred (AOR 10.7, 95% CI 2.26-51.0) and having cuts or wounds on the hands while handling the carcass (AOR 16.8, 95% CI 3.89-73.0). Community practices encouraging handling of infected carcasses were very common.

**Conclusion:**

Anthrax in Tsholotsho resulted from handling of infected carcasses. To avoid future outbreaks we recommend annual cattle vaccinations. The District must also hold regular zoonotic meetings and strengthen community surveillance for anthrax through health education campaigns.

# Sub article: Maternal Death Notification System Evaluation in Mashonaland Central Province, Zimbabwe, 2012

**Published:** 2015-07-22

**Authors:** Tendai Madziro-Nyagura

**Affiliations:** 1 Zimbabwe Field Epidemiology and Training Program

## Abstract

**Background:**

The Maternal Death Notification System (MDNS) in Mashonaland Central province had not been performing well with non-submission of notification forms to head office, submission of incomplete forms and irregularity of maternal mortality meetings reported in a provincial review meeting. The study was carried out to evaluate the performance of the MDNS in the province.

**Methods:**

An evaluation was carried out in 4 out of 8 randomly selected districts and at provincial level using CDC guidelines for surveillance systems evaluation. Information on usefulness and system attributes was collected using checklists and interviewer administered questionnaires to 17 primary respondents and seven key informants. Maternal death notification forms (MDNFs) for 2010 and 2011 were also reviewed to assess data completeness and quality.

**Results:**

Although all respondents reported usefulness of the MDNS, there was no evidence of data use at all levels. The MDNS only recorded 2 of the 53 deaths picked up by the T5 surveillance system in 2010. None of the health workers knew the timeline of submission to head office. In 2010 and 2011, none of the MDNFs reached national level on time. Reasons for untimely notifications included hierarchy of submission (14/17), unavailability of medical officers to complete forms (12/17) and shortage of staff (8/17). Using the paper-based system, the cost of notifying one maternal death in the province was estimated to be US$75.77.

**Conclusion:**

The MDNS was unstable, untimely and not sensitive such that in the current state it was unable to meet its objectives. Revival of maternal mortality meetings, revision of the notification hierarchy and making the MDNS electronic can help improve the system performance and cut the cost of reporting. Following the results of this study, an Epi-Info based MDNF was developed in the province and is being considered for possible roll-out to the rest of the country.

# Sub article: Epidemiology of the 2008-9 and 2010 Cholera Outbreaks in Kadoma City, Zimbabwe

**Published:** 2015-07-22

**Authors:** Brian Maponga

**Affiliations:** 1 Zimbabwe Field Epidemiology and Training Program

## Abstract

**Background:**

Kadoma City experienced Cholera outbreaks in 2008-9, and 2010, affecting 6,393 and 123 people, respectively. The study was conducted to compare the epidemiology of the two outbreaks.

**Methods:**

We carried out secondary data analysis of outbreak line lists for the 2 outbreaks. Proportions, means were generated and compared using the Chi Square test at 5% level of significance.

**Results:**

Cholera cases were similar by gender and age, with the 20-30 years age group being most affected. Rimuka township contributed 80% and 100% of city cases in 2008-9 and 2010, respectively (p = 0.000). In 2008-9, 91% of Cholera cases did not present within 2 days of onset compared to 98% in 2010. The 2010 outbreak evolved faster, resulting in higher proportion of cases being managed in Cholera Treatment Centres. Case Fatality Rate (CFR) was 2% in 2008-9, and 3.3% in 2010 (p =0.31). At least 70% of deaths occurred in treatment centres in Rimuka for both outbreaks.

**Conclusion:**

The 2008-9 and 2010 Cholera outbreaks were similar by age and gender. Rimuka Township was most affected by the outbreaks. There was worsening of delay in seeking treatment. The 2010 outbreak was more rapid, leading to early opening of Cholera Treatment Centres. CFR was consistently above 1%, being mainly institutional. There is need to strengthen case management through training and supervision. Health education on early treatment seeking should be strengthened, and evaluated.

# Sub article: Salmonella Outbreak Investigation at Dendera High School, Mudzi District, Zimbabwe, November 2011

**Published:** 2015-07-22

**Authors:** Simon Nyadundu

**Affiliations:** 1 Zimbabwe Field Epidemiology and Training Program

## Abstract

**Background:**

More than 85 students developed Diarrhea after eating food at a boarding school. This study set out to determine factors associated with contracting the Diarrhea illness and make recommendations to curb the outbreak and prevent future outbreaks.

**Methods:**

An Unmatched 1:1 case control study was done at Dendera, a mixed day boarding school, in Mudzi District, Mashonaland East Province, Zimbabwe. Case: was a student who reported Diarrhea and any other symptom such as abdominal cramps, vomiting, fever, headaches between the 17th and 22nd November 2011whislt a control was a student who did report any Diarrhea. Structured questionnaires were used to collect data from 80 cases from the line list and 80 controls, who were systematically selected. Environmental survey of the school was done. Clinic and patient records were reviewed. Stool, food and water samples were analyzed. Epi-Info was used to calculate means, proportions control for confounding, identify effect modification, and calculate 95% confidence intervals and to do stepwise logistic regression.

**Results:**

A total of 80 cases and 80 controls were interviewed. Factors associated with contracting Diarrhea were eating makanyanise (a mixed vegetable mince) [0R 2.6:95% CI1.03-6.39], washing hands after toilet use (OR = 0.48; 95% CI: 0.25-0.91) and washing hands before eating meals (OR = 0.32; 95% CI: 0.16-0.63). Resident students (OR 21.8; 95% CI: 6.5-72) were affected more than the non-residents. Environmental survey revealed poor sanitary conditions and facilities and lack of water at the school. Salmonella was isolated from only 2 of 40 stool specimens.

**Conclusion:**

The outbreak was due to salmonella isolated from makanyanise which was prepared under unhygienic conditions. Poor sanitary conditions, water shortages and poor food handling practices facilitated the spread. The District Health team has to address the laboratory weaknesses and poor case management identified in this study.

# Sub article: Evaluation of the Sentinel Surveillance System for Rotavirus Gastroenteritis at the Mother and Child Centre in Yaoundé - Cameroon, 2007 – 2011

**Published:** 2015-07-22

**Authors:** Yomog Mathieu

**Affiliations:** 1 Central African Field Epidemiology and Laboratory Training Program

## Abstract

**Background:**

The Sentinel Surveillance System (SSS) for rotavirus gastroenteritis was established in the Mother and Child Centre (MCC) in Yaoundé in 2007 to collect data for the introduction of the anti-rotavirus vaccine. Since its inception, this system has never been evaluated. This study was done to evaluate the SSS for rotavirus gastroenteritis in MCC, specifically to assess its attributes and usefulness.

**Methods:**

We conducted a documentary review of data from MCC and its three satellite sites. We administered a questionnaire to 36 key informants from these four sites. We used the CDC guidelines for evaluating surveillance systems as reference for the evaluation of attributes and the usefulness of the system.

**Results:**

The system's flow chart is simple with 34(94.4%) of the 36 respondents having good mastery of the case definition. Twenty-seven (75%) accepted that surveillance activities make up part of their routine task. The system is supported by WHO and CDC. It is flexible as the form could notify other diseases. Data quality showed that, out of 73 056 spaces on the form 58 402 (79.94%) were filled. All the forms were counterchecked by a different person for validity. The system operated uninterrupted from 2007 to 2011, making it stable. For sensitivity, only 1664(43.6%) were captured out of 3819 registered cases during these five years, and 621 of them were confirmed by EIA giving a positive predictive value of 37%. The system was useful in determining the most affected age groups, and the seasonality of the rotavirus strains circulating in Yaoundé.

**Conclusion:**

The surveillance of rotavirus gastroenteritis at the MCC is simple, acceptable, flexible, stable, and useful. However its positive predictive value and sensitivity were unsatisfactory. Data quality was good. Health Authorities need to improve the notification of the disease and promote the collection and testing of specimen from all notified cases.

# Sub article: Rabies Outbreak investigation in pigs and increased dog bites among humans in Wakiso District, 2012

**Published:** 2015-07-22

**Authors:** David Mugabi

**Affiliations:** 1 Uganda Field Epidemiology and Training Program

## Abstract

**Background:**

Rabies caused by Lyssa virus is transmitted to humans through infected animal bites. A suspected Rabies outbreak in pigs and increased dog bites among humans was reported in Wakiso District. Two pig farms and the surrounding community were reported affected. We investigated the outbreak and instituted public health control measures to avert the problem.

**Methods:**

Record reviews from health centre were done and 68 cases were listed as suspected Rabies among humans. Visits to 2 farms with dog bite cases among pigs were done. Three key informant interviews with the veterinary officer, health staff and community leader and 3 focus group discussions with community members were done. 10 brain samples from affected pigs were analyzed in laboratory using fluorescent antibody technique.

**Results:**

In humans among the 68 dog bite cases, 40(58.8%) were aged 5-12 years and 28(41.2%) aged 20-30 years. Males were 36(52.9%) of the cases. In pigs,3(30%) of the brain samples were positive for Negri bodies in the brain cytoplasm. The 10 pigs bitten were not housed.

**Conclusion:**

Rabies outbreak was confirmed in pigs and high number of dog bites in humans was attributed to irregular dog vaccination and presence of stray dogs. Rabies community sensitization was done and mass dog vaccination against Rabies was done in the affected communities. People were advised to seek medical help from health facilities as soon as possible whenever bitten by an animal.

# Sub article: Cross-border Measles outbreak in a scattered and highly mobile population - Somali region, Ethiopia, 2012

**Published:** 2015-07-22

**Authors:** Kasim Mahdi

**Affiliations:** 1 Ethiopia Field Epidemiology and Training Program

## Abstract

**Background:**

Measles is a leading vaccine preventable childhood disease, which has been designated for elimination by the World Health Organization. Measles is still responsible for an estimated 10 million cases and 164, 000 deaths worldwide each year. On January 15, 2012, we received a report of unusual increase of unidentified acute febrile illness from Elkeri- District of Somali region in Ethiopia. We investigated the outbreak to identify etiology of agent and implement control measures. We also determined Measles vaccination coverage and factors related to the outbreak.

**Methods:**

A suspected case of Measles was any person presenting with fever and maculopapular rash and a cough, coryza or conjunctivitis while a confirmed case was IgM positive for Measles. We conducted active case search from health facilities and house to house visit and collected serum sample from five suspected patients. We interviewed parents or caregivers to collect data on vaccination history and selected socio-demographic characteristics. We also assessed health service delivery status; cold chain and immunization coverage of the District.

**Results:**

We identified 71 suspected Measles cases (Attack rate: 2.8 /1000) and three community deaths (case fatality rate (CFR): 5.6%). Four of the five samples were Measles-IgM positive. The most affected age was 5-9 years old (AR: 6.3/1000) followed by age 1-4 years old (AR: 5.6). All death and 52 (73.4%) of the cases were not immunized. The index case was imported from Somalia. Vaccination coverage of the District was 25%. No health facility was providing routine vaccination in affected kebeles. 3/4 refrigerator (75%) were malfunctions.

**Conclusion:**

We confirmed imported Measles outbreak in Somalia region of Ethiopia with an index case from neighboring Somalia country. Un-vaccination and primary vaccine failure may have increased susceptibility. We recommended to improve cross-border routine and supplementary vaccination, surveillance system and expand national supplementary vaccination from the current under 5 up to 5-9 age in low immunization coverage area.

# Sub article: Evaluation of the Effectiveness of Veterinary Disease Surveillance system: A case study of Mubende District, 2012

**Published:** 2015-07-22

**Authors:** Grace Asiimwe

**Affiliations:** 1 Uganda Field Epidemiology and Training Program

## Abstract

**Background:**

Public health surveillance is done to assess the health status of a population, establish public health priorities, and reduce the burden of disease in a population. In Uganda, notifiable diseases including hemorrhagic fevers are a constant threat to the health of both animals and humans. Rabies and Ebola were confirmed in central Uganda in 2012 where animals are reservoirs for the diseases. However the veterinary disease surveillance system has long not been evaluated for its effectiveness in early disease detection. The study evaluated the effectiveness of the Veterinary disease surveillance system in Mubende District, central Uganda.

**Methods:**

We reviewed and abstracted secondary data from monthly reports of 15 reporting subcounties of the District 2007 to 2011. Key informants were interviewed using an interview guide. We assessed the District surveillance system attributes using CDC guidelines. Data was entered and analyzed using Epi-info version 3.5.1

**Results:**

Only 34% of the total monthly reports were received at District in 2007, 70% in 2008, 43% in 2009, 11% in 2010 and 4% in 2011. Of the expected field reports 38% were received before 7th and 97% before 15th of the next month at the District. (Ministry expects reports by 15th). Standard data collection tools not used by all staff, structure hard in data flow, understaffing (16%), no zero reporting, no laboratory at district; use of visual surveillance by staff and data kept in hard, inconsistency in reporting and bulky surveillance tool. On average, 32% of the expected reports were received at District in five years. The system was able to capture three outbreaks but unable to confirm any diseases including Rabies and Trypanosomiasis. Key informants showed that low performance was due to no incentives to staff and ministry restructure.

**Conclusion:**

The veterinary disease surveillance system for Mubende District is not optimal. The ministries of health and veterinary should recruit staff and sensitise on disease surveillance and use of standard surveillance tools. Provide basic laboratory facilities to screen and confirm diseases. Training of Community based animal health workers and village health teams in surveillance, zoonotics and epidemic disease detection, prevention and control.

# Sub article: Rotavirus Diarrhea Outbreak among under-fives in Korogwe and Tanga city (Tanzania) March- April 2013

**Published:** 2015-07-22

**Authors:** Clarah Adel

**Affiliations:** 1 Tanzania Field Epidemiology and Laboratory and Training Program

## Abstract

**Background:**

Rotavirus is the major cause of Diarrhea and vomiting in young children worldwide. The infection is highly contagious and may lead to severe dehydration and death. More than a third of 1.34 million Diarrhea deaths in under-fives and 40% of the 9 million Diarrhea-related hospitalizations worldwide are due to rotavirus disease. On 9th April 2013, Ministry of Health and Social Welfare was notified of a suspected Rotavirus Diarrhea outbreak in Tanga region. A total 227 suspected cases and 5 deaths (CFR = 2.2) were reported. An outbreak investigation team was sent to establish the existence of an outbreak, confirm diagnosis and set control measures.

**Methods:**

A case control study was conducted. A case was any person living in Tanga City or Korogwe District from 9th March to 29th April, 2013 with fever, stomach cramps, vomiting, and Diarrhea. Control was any person living in neighborhood of a case with no history of suffering Diarrhea illness at the same period. Data was obtained by record review and active case finding. Stool analysis was done. Univariate, bivariate and multivariate analysis were done using Epi-Info version 3.5.1.

**Results:**

There were 294 cases cumulatively with 5 deaths (CFR = 1.7%). Majority 286 (97.3%) were <5 years. Sex was proportionate distributed. All cases presented with Diarrhea 294 (100%), 189 (64.2%) with vomiting and 97 (33%) with fever. A total of 10 stool samples tested positive for rotavirus infection. A total of 116 children participated in a case control study. Factors associated with higher odds of Rotavirus Diarrhea included aged 7-12months [AOR = 26.27; 95% CI: 3.82-180.5], history of recent travel [AOR = 21.09, 95% CI: 2.38-187.25], Diarrhea contact at home [AOR = 5.70, 95% CI: 1.42-22.97]. Mixed breastfeeding was a risk in bivariate analysis only [OR = 6.46, 95% CI: 2.66-15.72].

**Conclusion:**

An outbreak of Rotavirus was confirmed mostly affecting children aged between 7-24 months. History of travelling and presence of Diarrhea contact at home were risk factors for this outbreak. Health education on proper food handling and thorough hand washing was given. With introduction of the Rotavirus vaccine in 2013 in Tanzania, the burden associated with Rotavirus will be minimized.

# Sub article: Evaluation of Laboratory-Based Measles Surveillance, Tanzania, 2011

**Published:** 2015-07-22

**Authors:** Theophile Clemence Malibiche

**Affiliations:** 1 Tanzania Field Epidemiology and Laboratory and Training Program

## Abstract

**Background:**

Measles is among the top ten causes of death in children less than five years of age in many African countries, Tanzania is among the countries which are in Measles reduction phase, implementing comprehensive reporting of case based Measles surveillance. Measles laboratory based surveillance was introduced in Tanzania in 2003 aiming at diagnosing and provide epidemiological proof in line with elimination strategies. This evaluation aimed to determine whether Laboratory based Measles surveillance meet its purpose and objectives in 2011 and to evaluate the system attributes.

**Methods:**

CDC's guideline for evaluating surveillance system was used and it was complemented by reviewing of reports and documents (MUHAS-National Measles laboratory, EPI); and interviewing stakeholders using structured questionnaires. Data was analyzed by Epi-Info software.

**Results:**

The system was useful in Measles detection. Among 428 samples that were collected in 2011, 44.9% were confirmed positive for Measles. Only 75% of health workers had knowledge regarding the surveillance system. The system is donor fund dependent. No feedback to lower health delivery level. Only 25% of staff were trained on surveillance system. Data quality ranged from 43.6% to 76.2%. Form incompleteness and incorrectness ware observed from staffs that had partial training or untrained. Timeliness on sample delivery was 1.3%, difficulties in transportation accounts for late sample submission to the laboratory however quality of the samples were 100%. The system is flexible to incorporate Rubella cases. All the regions in the country participate in the reporting. The system was simple, flexible, acceptable, sensitive and representative, however donor fund dependent and unstable.

**Conclusion:**

The system is meeting its intended purposes in detection of Measles, however, we recommend strengthening monitoring of Measles laboratory network within the country, timely reporting and transportation and training to all surveillance sites.

# Sub article: Enhanced Surveillance for Improved Maternal Health: a Case Study of Maternal Death Reviews in Uganda

**Published:** 2015-07-22

**Authors:** Emily Atuheire

**Affiliations:** 1 Uganda Field Epidemiology and Training Program

## Abstract

**Background:**

Maternal death reviews are necessary in understanding why women could have died and developing an effective response. In 2008, the Uganda Ministry of Health (MOH) mandated all hospitals to notify, and review report maternal deaths occurring at their facilities to the MOH. Data from maternal death review (MDR) forms should be routinely collected using standard Health Management Information Systems (HMIS) tools. Complete and accurate identification of all deaths associated with pregnancy is a critical first step in the prevention of maternal deaths. Having a clear understanding of the magnitude and causes of pregnancy-associated mortality is key in formulating comprehensive strategies to prevent these unfortunate deaths among young and healthy women. We analyzed MDR data to understand the pattern of medical causes of maternal deaths and provide information that effectively guides actions to eliminate preventable mortality.

**Methods:**

We extracted data for the period 2009-2011from HMIS and MDR forms that were routinely submitted to the national data base (MOH Resource Centre) by health facilities within the national health system. Data was cleaned to minimize errors. The factors analyzed include routine reporting and notification of maternal deaths, death by age, and pregnancy status at admission, antenatal attendance, parity, place of delivery, severity of illness, duration of stay in facility, referrals, HIV status and cause of death as reported on the review forms. Excel program was used to calculate descriptive statistics.

**Results:**

Of the 116 hospitals, only 22(19%) submitted MDR forms. All 315(100%) MDR forms submitted were analyzed. 4419 deaths were reported through HMIS. Notified deaths were 13(0.6%), 45(3.6%), 129(12.8%) in 2009, 2010 and 2011 respectively. The five medical causes of death are hemorrhage (39%, n = 123), abortion (13%, 40 deaths), hypertensive disorders of pregnancy (10%, n = 31), uterine rupture (8%, n = 26) and sepsis (8%, n = 26). About 3/4 of the deceased were aged below 30years (70%, n = 222), and 1/4 were teenagers, the youngest being 14 years. 32%(n = 101) died in labor and 25%(n = 79) were critically ill on admission. 51.4%(n = 162) of the deceased spent over 24hours in the facility before death. Nearly half (49.2%, n = 155) had no records on HIV testing 22.7%(n = 72) had attended the World Health Organization recommended four antenatal care visits.

**Conclusion:**

Maternal killers are similar to those already known and preventable. Strengthening MDR and death notifications is key in designing targeted interventions for reducing maternal deaths; and probably required before scaling up the program.

# Sub article: Cholera Outbreak in Bungudu Local Government Area - Zamfara State, Nigeria; July, 2011

**Published:** 2015-07-22

**Authors:** Kabiru Getso

**Affiliations:** 1 Nigeria Field Epidemiology and Laboratory and Training Program

## Abstract

**Background:**

Cholera remains a major public health problem in Nigeria usually occurring as large scale outbreaks leading to high morbidity and mortality. Between June-July, 2011; we investigated suspected Cholera outbreak in Tofa and Samawa wards in Bungudu Local Government Area (LGA) of Zamfara State, northern Nigeria to confirm the outbreak and implement targeted interventions.

**Methods:**

We conducted cross sectional study. A suspected Cholera case was defined as any resident of Tofa or Samawa ward with at least three episodes of acute watery Diarrhea with or without vomiting between 30th May and 4th July, 2011. We reviewed hospital records and used case-based line listing to collect patients’ data. Environmental assessment was conducted. We collected and analyzed 5 stool and 2 water samples using Thiosulfate Citrate Bile Salts Sucrose and Mac Conkey agars. Data were analyzed using Epi-Info and Microsoft Excel.

**Results:**

Altogether, 111 cases were recorded (attack rate 0.59%) with 5 deaths (Case fatality rate: 4.5%). The Male to Female Ratio was 1:1. Median age was 5 years with age range of 4 months to 65 years. About half of cases (47.7%) were children between 0-4 years (p = 0.01), age-specific attack rate for 0-4 years age group = 1.06%. All (100%) stool specimens yielded V. cholerae 01, serotype Ogawa. Environmental assessment revealed unsanitary conditions and inadequate and unsafe water supply. Water samples tested negative for V. cholerae but yielded growths of E. coli.

**Conclusion:**

Bungudu LGA had confirmed Cholera outbreak. We strengthened case management and conducted health education focusing on personal hygiene and environmental sanitation. Advocacy visit was paid to local Authorities to intensify health education and provide adequate and potable water to affected communities.

# Sub article: Polio Outbreak Investigation and Response in A Previously Polio Free State in North Central Nigeria – 2012

**Published:** 2015-07-22

**Authors:** Shember Igbabul

**Affiliations:** 1 Nigeria Field Epidemiology and Laboratory and Training Program

## Abstract

**Background:**

Globally, Nigeria is one of the three countries currently endemic with poliomyelitis. In November 2012, routine surveillance picked up a paralytic polio child from Doma, Nasarawa State, Nigeria. This was the first report after three years interruption of polio transmission in this part of Nigeria. We visited the area to define the magnitude of the outbreak and recommend control measures to stop further transmission.

**Methods:**

We reviewed health-facility records, surveillance and laboratory data, interviewed stakeholders, conducted 30 household immunization coverage survey and house-to-house Acute Flaccid Paralysis (AFP) case search in the polio patient's community. The case definition for AFP was any child under 15 years of age with sudden weakness or floppiness of one or more limbs or any person of any age in whom a clinician suspects poliomyelitis while paralytic polio was AFP case with wild polio virus isolation in stool.

**Results:**

The polio child had taken 9 doses of oral polio vaccine (OPV). The family dwelling had poor drainage and refuse disposal facility. 11/16 (69%) of family members were migrant traders. Household survey revealed an active unreported AFP case in the neighborhood. Only 20% of children 0-5years had received 3 OPV doses in routine immunization (RI). Zero dose RI up-take was 11/30 (37%) and reason for this was mostly no felt need 9/10(90%). Only 11/29 (38%) of children 0-5 years had received OPV in the last supplementary immunization activities (SIA) held 8 months earlier. 12/14 (86%) non-vaccination in last SIA was due to displacement of affected families during communal conflicts.

**Conclusion:**

Poor immunization coverage, unsanitary dwelling, and migrant trading were risk for polio transmission. Evidence-based recommendations of findings initiated immediate mopping-up and a state-wide polio response immunization campaign.

# Sub article: Measles Outbreak in Adults, Axum, Ethiopia, 2012

**Published:** 2015-07-22

**Authors:** Taame Haftom

**Affiliations:** 1 Ethiopia Field Epidemiology and Training Program

## Abstract

**Background:**

Comprehensive immunization programs in Ethiopia have improved nationwide vaccination coverage from 42% in 2002 to 79% in 2012, and Measles has been targeted for elimination by 2020. We received reports of a Measles outbreak in Axum Town Tigray Region of Ethiopia and investigated to identify additional cases and contacts, prevent spread, identify risk factors and implement control measures.

**Methods:**

We used the World Health Organization (WHO) definition for a suspected Measles patient as any person with fever; maculopapular rash; and either cough, conjunctivitis, or coryza or any person suspected of Measles by a clinician. Vaccination status was determined by case-patient recall. We investigated all suspected Measles patients from November 4 to December 4, 2012 and obtained 5 blood samples for laboratory confirmation. We conducted a matched case control study of 28 Measles cases and 30 controls.

**Results:**

We identified 3 laboratory confirmed and 25 epidemiologically linked Measles patients. There was one death. The median age of the Measles patients was 21years and twenty-seven (96%) were older than 15 years. Sixteen (57%) were students at Axum University. Twenty-seven (96%) of the Measles patients had not been vaccinated for Measles. Being vaccinated was highly protective of illness (OR= 0.02, 95% CI= 0.002-0.16).

**Conclusion:**

We identified the fourth Measles outbreak among adults since 2011 in the Tigray Region of Northern Ethiopia. We believe that the improvement in immunization rates among children in the region has created a demographic shift where Measles is increasingly affecting adults. We are recommending that Measles control strategies in the region include protocols that identify, manage, and immunize both children and at-risk adults - especially in high-density populations such as schools, universities, and prisons.

# Sub article: Evaluation of Veterinary and Human Surveillance Systems for Rabies detection in Nakasongola, Uganda, 2012

**Published:** 2015-07-22

**Authors:** Fred Monje

**Affiliations:** 1 Uganda Field Epidemiology and Training Program

## Abstract

**Background:**

Effective disease control necessitates good public health surveillance systems. However, in Nakasongola District, the high persistence of animal bites (suspected cases of Rabies), raises doubts on the performance of the surveillance system. On average, 429 animal bites annually were recorded in humans from 2008 to 2011 in the district. Hence, our objective was to evaluate veterinary and human surveillance system for Rabies detection and assess system attributes so as to design measures to improve its performance.

**Methods:**

This was a cross-sectional study conducted from 26th, April-30th, June 2012 in Nakasongola District. Suspected cases of Rabies were abstracted from the District health records. We reviewed 2008-2011 District veterinary surveillance records. Conducted Key informant interviews of the veterinarians and health workers. Used CDC updated guidelines for Surveillance evaluation. Entered and analyzed data using Epiinfo.

**Results:**

Veterinary surveillance system attributes: Simplicity: standard form was not used, Flexibility: 4(36.4%) out of 11 personnel, no budget allocation. Data quality: Completeness of reporting sub-counties was 72.2% on average, among the received reports; only 50.1% of them had all variables filled. Acceptability: Completeness of reporting sub-counties was 72.2%, sub-county vets report monthly to District. Timeliness: 2 (22.2%) of 9 reports received monthly before 15th of following month. Sensitivity: suspected cases of Rabies outbreaks were not detected and recorded. No formal collaboration between District veterinary and human sectors. However, in Human surveillance system attributes: Simplicity: standard form was used, Flexibility: 305 (67.3%) out of 453 health workers, has budget allocation. Data quality: Completeness of reporting health facilities was 90.6%, among the received reports, 95.7% of them had all variables filled. Acceptability: completeness of reporting health facilities was 90.6% on average, health facilities report weekly and monthly to the District. Timeliness: 29 (90.6%) of 32 reports received before 15th of following month. Sensitivity: Suspected Rabies outbreaks detected including 6 human deaths in 2010.

**Conclusion:**

Human surveillance system detects suspected cases of Rabies outbreaks. However, veterinary surveillance does not. To improve on Rabies detection, veterinary sector should use standard form which captures animal bites and streamline data flow. District veterinary and health worker should strengthen their collaboration.

# Sub article: An outbreak of Cerebrospinal Meningitis (CSM) in Kebbi State, Nigeria - February 2013

**Published:** 2015-07-22

**Authors:** Mikhail Abubakar

**Affiliations:** 1 Nigeria Field Epidemiology and Laboratory and Training Program

## Abstract

**Background:**

The largest burden of meningococcal disease occurs in an area of sub-Saharan Africa known as the meningitis belt, which stretches from Senegal in the west, Nigeria in the middle to Ethiopia in the east. On 27 February 2013, cases of suspected cerebrospinal meningitis including deaths were reported in Kambazar Fulani, Gwandu Local Government Area (LGA), Kebbi state, Nigeria. We investigated the outbreak to identify risk factors for infection and institute control measures.

**Methods:**

We conducted descriptive and case control studies. We defined a case as any person from Kambazar Fulani with sudden onset of fever (>38.5°C rectal or 38.0°C axillary) and any one of the following signs: neck stiffness, altered consciousness, other meningeal signs from 15-27 February 2013 and a control as any person from the neighborhood of cases without the defined symptoms. We actively searched for cases and conducted additional environmental assessment. We collected data on socio-demographic characteristics, living conditions and other risk factors. We performed serological analysis on cerebrospinal fluid (CSF) specimen.

**Results:**

We identified 28 cases among 252 persons of which eight died (attack rate (AR): 11%, case fatality rate: 29%). The AR was highest among 6-11 years and 30-35 years old (29% each). The incidence was highest (79%) in one of the areas were large number of persons are living in one household. Compared with 28 controls, the 28 cases did not differ in terms of age and sex. However, among households, cases with Meningitis sleep more in unventilated rooms than controls (Odds ratio: 4.5, 95% confidence interval: 1.46-13.89). An environmental assessment showed a cluster of small-unventilated rooms per household in more than 50% of the houses. Two out of three specimens tested positive for both H. Influenza e type b and S. pneumoniae.

**Conclusion:**

Overcrowding and inadequate ventilation where identified as major risk factors for infection. H. Influenza e type b and S. pneumoniae may have been the source of the outbreak. Cases were isolated and broad-spectrum antibiotics (ceftriazone) administered which resulted in the control of the outbreak. We recommended to Kebbi State Government for immediate CSM vaccination throughout the State to prevent future outbreaks.

# Sub article: Lassa Fever Outbreak investigation, Plateau State - Nigeria, 2013

**Published:** 2015-07-22

**Authors:** Lilian Okeke

**Affiliations:** 1 Nigeria Field Epidemiology and Laboratory and Training Program

## Abstract

**Background:**

Lassa fever (LF) is an acute viral hemorrhagic zoonotic disease associated with high morbidity and mortality. Globally, between 300,000- 500,000 cases of LF with 5000 deaths occur annually. Plateau State is among the states in Nigerian that have reported frequent LF outbreaks in the past. In December 2012, a suspected case of LF was reported in Plateau State. We conducted an investigation to describe the magnitude of the outbreak, conduct environmental assessment, and institute control measures.

**Methods:**

We reviewed hospital records, interviewed suspected cases, and healthcare workers to obtain information on suspected cases socio-demographic characteristics. We collected specimens from suspected cases and sent to LF reference laboratory for analysis. A suspected case of LF was defined as any person with acute illness of < 3 weeks’ duration and temperature ≥ 380C, with no response to effective antimalarial after 48 hours between 26th January and 2nd March, 2013 at Bassa, Yaoundé South, Yaoundé North, Riyom, Barkin Ladi, and Kanke Local Government Areas in Plateau State. A confirmed case was defined as a suspected case with laboratory confirmation. Environmental assessment was conducted to observe for rat infestation, proper storage of food items and proper waste disposal around 11 affected communities in the 6 LGAs.

**Results:**

Eleven suspected cases were reported with 4 deaths (CFR 36.4%) and attack rate of 3.14 per 100,000 persons. Median age was 26 years (range 2-45 years), six cases (54.5%) were females. Of the 6 blood samples analyzed, 4 (66.6%) tested positive for LF IgM antibodies. There was evidence of burrows indicating rat infestation, lack of proper storage of food items and no proper waste disposal around the 11 affected households. A one day public health sensitization talks on signs and symptoms, preventive and control measure of LF was organized for healthcare workers and the affected communities.

**Conclusion:**

This investigation confirmed the outbreak of Lassa fever. The lack of proper storage and no proper waste disposal could be an attraction for the vector. Public health sensitization talks on preventive and control measures of LF should be sustained.

# Sub article: Evaluation of Rabies Surveillance in the Upper West Region, Ghana, 2008-2012

**Published:** 2015-07-22

**Authors:** Eugene Yelfaanibe

**Affiliations:** 1 Ghana Field Epidemiology and Laboratory and Training Program

## Abstract

**Background:**

Rabies is a fatal zoonotic disease of serious public health importance. Annually, one person dies of Rabies in the Upper West Region of Ghana. The objective of this evaluation is to assess whether the surveillance system is meeting its objective of early detection of Rabies and prompt public health action or not.

**Methods:**

We used the Centre for Disease Control and Prevention Guidelines on Evaluation of Surveillance System. We reviewed the outbreaks of scheduled diseases reporting formants and analyzed data on Rabies at the national, regional and District levels. We engaged stakeholders and discussed our findings with them.

**Results:**

We found out that Rabies is one of twenty-eight reportable diseases to AU-AIBAR. The System comprised of community animal health workers, District, regional, national veterinary offices/laboratories/staff and AU-AIBAR/OIE/FAO. Information flow was satisfactory. The case definition was very sensitive. We observed inadequate budgetary support and late releases of funds threatened the stability of the system. Feedbacks and quality of data were poor. Timeliness of the system (33% early) was poor. The system was simple, acceptable and sensitive. PVP was 33.3%

**Conclusion:**

We concluded that the Rabies surveillance system was not meeting its objectives. We therefore made recommendations to improve upon the system. To improve upon the data quality, District veterinary officers should organize quarterly in service training for staff on quality data collection and filling of the outbreak of scheduled diseases reporting formant. To improve upon the timeliness of reporting, the regional veterinary officer should query/sanction officers who report cases late. To make the system stable, District coordinating directors should allocate and release early adequate funds from the common fund for surveillance.

# Sub article: An unusual outbreak of Pharyngo-tonsillitis caused by *Aeromonas hydrophila* in Karaye, Kano State - Nigeria 2012

**Published:** 2015-07-22

**Authors:** Usaini Bala

**Affiliations:** 1 Nigeria Field Epidemiology and Laboratory and Training Program

## Abstract

**Background:**

In Nigeria, pharyngo-tonsillitis, an inflammation of the pharynx and the tonsils, is a common illness in both adults and children. In Kano State the incidence of pharyngo-tonsillitis is higher during dry season (November to May) but outbreaks are relatively uncommon. On 6th August 2012, Kano State Ministry of Health received a report of an outbreak of Pharyngo-tonsillitis in Karaye, Kano State. The affected individuals complained of fever, sore throat with difficulty and /or painful swallowing and cough in some. We formed a rapid response team that investigated to describe the outbreak, identify the causative organism and institute public health actions.

**Methods:**

We conducted a descriptive study. We defined a case as any residence of Tudun kaya, Daura, Kwanyawa, Turawa and Yanmedi wards with fever and sore throat between 27th July, 2012 and 19th September, 2012. We reviewed health records of five health facilities and line-listed all cases meeting the case definition. We conducted active case search in the community to identify additional cases. We collected four throat swabs specimen and performed culture and sensitivity test to isolate the causative organism and determine its antimicrobial susceptibility.

**Results:**

A total of 462 cases were reported with nine deaths (Case fatality rate: 1.9%). Of the 462 cases, 313 (67.7%) were females. Median age was 9.5 yrs with age range 0 .5-70 yrs. The attack rate was highest among 0 - 5 year olds and (0.11%); lowest among 40year olds and above (0.01%) Cases first occurred on 27/07/2012, reached its first peak on 31/07/2012, subsided over four weeks the second peak was observed on 28/08/2012 and decreased on 19/09/2012. Three of the four (75%) throat swab specimens tested positive for Aeromonas spp, and further Genomic sequencing revealed all the organisms as Aeromonas hydrophila.

**Conclusion:**

This outbreak of Pharyngotonsillitis was caused by Aeromonas hydrophila most likely an epidemic strain. Health care workers were sensitized about the disease and its management. Health talks were given to the community on the dangers of harmful traditional tonsillectomy and uvulectomy practices.

# Sub article: A Review of Five Year Data on Bovine Tuberculosis (BTB) in the Volta Region, 2006-2011

**Published:** 2015-07-22

**Authors:** Vitus Burimuah

**Affiliations:** 1 Ghana Field Epidemiology and Laboratory and Training Program

## Abstract

**Background:**

Bovine Tuberculosis is a chronic infection cause by Mycobacterium bovis, which infects nearly all warmed-blooded animals including humans. Wild ruminants and carnivores are the natural reservoirs of the infection. Approximately 85% of cattle and 82% of the human population of Africa live in areas where Tb is partly controlled or neglected. In Ghana, a study conducted showed that out of 70 persons diagnosed with Tb, 3% was due to M. bovis. The objective of this study is to determine the distribution of bovine Tb in both cattle and human populations in the region to provide information to guide control activities.

**Methods:**

We conducted the study in the Volta region of Ghana using secondary data from abattoirs, Regional Veterinary Laboratory and the National Tb program. Data was analyzed by person, place and time using Microsoft Excel version 2007. Distribution by districts of bovine TB in cattle and Tb in humans were compared using graphs.

**Results:**

Between 2004 and 2008, 149 cattle (0.7%) were diagnosed with bovine Tb of which only 17 (11.41%) were laboratory-confirmed. Three thousand three hundred and nine (0.3%) persons were diagnosed with Tb between 2006 and 2008. There was no sub-typing of the mycobacterium. The study revealed that communities where BTB was detected in cattle, there were persons affected with TB.

**Conclusion:**

Communities where BTB was detected in cattle also had humans afflicted with Tb. However, the practice of not sub-typing the mycobacterium in affected persons makes it unclear if M. bovis is responsible for the infections in both cattle and humans in these communities. Health facilities in the region should be empowered by the Ministry of Health (MOH) to subtype all diagnosed Tb cases. Also, VSD should ensure collaboration between veterinary laboratories and field veterinarians for the confirmation of all suspected cases of BTB in the region. They should provide logistics to enhance clinical and postmortem diagnosis of the disease. As a result, clinical diagnostic kits were sent to the region and the first intra-dermal tuberculin testing carried out in cattle in many parts of the region.

# Sub article: Assessing Multi Drug Resistance Tuberculosis in Coast Province, Kenya 2009-2012

**Published:** 2015-07-22

**Authors:** Hajara EL-Busaidy

**Affiliations:** 1 Kenya Field Epidemiology and Laboratory and Training Program

## Abstract

**Background:**

Kenya is one of the 22 high burden TB countries that collectively contribute about 80% of the world's TB burden. From 2003 to 2008, there were a total of 353 MDR TB cases. Coast Province in Kenya, with a high burden of HIV and poverty levels, has been reporting a high prevalence of MDR TB. We analyzed surveillance data to characterize MDR TB cases in the province.

**Methods:**

We retrospectively reviewed all MDR TB cases who had received treatment in Coast Province from 2009 to 2012. A case of MDR TB was defined as any laboratory diagnosis of TB resistant to rifampicin and isoniazid. Variables extracted included demographic, HIV status, history of drug abuse, and month of first sputum culture not growing the TB.

**Results:**

Forty-eight cases were reported; 11 (23%) completed treatment, 4 (8%) died, and 33 (63%) were currently on treatment. Thirty-four (71%) occurred in males and mean age was 34 years (SD = 8.8yrs, range 9-53 years). The prevalence of MDR TB in the province was 1.4/100,000 ranging between 0.3 and 2.9/ 100,000 in the districts. Twenty-eight (58%) cases were from Mombasa County (the capital town). Fifteen (30%) reported using illicit drugs. Nineteen (20%) cases occurred among HIV positive patients, and this increased from 11% in 2009 to 42% in 2012. Among the cases, 20 (42%) had been treated with first line drugs only once before diagnosing with MDR TB. 36 (75%) patients were culture negative by 3rd month of treatment.

**Conclusion:**

MDR TB diagnosis was made after only one treatment with first line drugs; this could mean they were primary MDR cases. There is increasing co-infection of MDR TB with HIV. MDR TB should be sought in all age groups and patients not responding to first line TB drugs and HIV cases should be screened for MDR as early as possible.

# Sub article: Descriptive analysis of program data related to occupational Post-exposure Prophylaxis for HIV at Maputo Central Hospital, 2011

**Published:** 2015-07-22

**Authors:** Paixão Torres

**Affiliations:** 1 Mozambique Field Epidemiology and Laboratory and Training Program

## Abstract

**Background:**

Occupational post-exposure prophylaxis for HIV consists of short-term anti-retroviral therapy (ART) to reduce the risk of HIV infection in health care workers (HCW). This program provides services to health professionals with potential exposures to HIV in the workplace, and establishes the notification of exposure, initial medical care, and orientation of the HCW regarding follow-up and chemoprophylaxis. For each occupational exposure, a form is completed with data regarding work location and function, years of service, and past occupational accidents. Also noted on this form are the time, location and nature of the accident and information regarding the source patient, including their diagnosis and HIV status. Blood samples are collected from the source patient for rapid HIV testing (Determine and Unigold). The objective of the present work is to provide a descriptive analysis of occupational accidents involving potential HIV exposure.

**Methods:**

A retrospective descriptive analysis was conducted based on data available from registry books and occupational exposure report forms from Maputo Central Hospital (HCM) in 2011.

**Results:**

There were 76 occupational accidents with potential HIV exposure reported during 2011, with a monthly average of 6.3 accidents per month, and the highest monthly total occurring in June with 10. Analysis by professional category showed 22 (28.9%) nurses and 15 (19.7%) physicians. Work location of accidents showed a predominance in services characterized by more complex procedures such as surgery (n = 15, 20%), emergency department (n = 15, 20%), and post-surgical ward (n = 11, 14%). The most common type of accident was needle sticks (n = 55, 72.4%). Test results in workers following the accidents showed 72 (95%) HIV negative and 4 (5%) HIV positive. Data on workers’ HIV serologic status prior to the accident were not available. Fifty-nine (78%) HCWs started post-exposure prophylaxis.

**Conclusion:**

Work related accidents can affect all HCWs; however, the severity of work related HIV exposures is associated with occupational category and type of lesion. Post-exposure prophylaxis (PEP) continues to be an important intervention to reduce the risk of occupational HIV transmission, especially in countries such as Mozambique with high population prevalence of HIV.

# Sub article: Evaluation of Tuberculosis infection prevention and control measures in four Counties in Rift valley, Kenya 2013

**Published:** 2015-07-22

**Authors:** Evans Kiprotich

**Affiliations:** 1 Kenya Field Epidemiology and Laboratory and Training Program

## Abstract

**Background:**

Tuberculosis (TB) is a bacterial disease that can be fatal if untreated. Any inadequacy in prevention and control of TB increases the chances of transmission. Tuberculosis Infection prevention and Control (IPC) measures are important in TB control. Emergence of drug resistant TB and high burden of TB/HIV AIDS in Kenya complicate the situation. The divisional of Leprosy TB and Lung disease (DLTLD) is scaling up IPC measures. This survey was done to evaluate TB infection control (IC) measures in health facilities in January 2013.

**Methods:**

We selected districts in four counties supported by Walter Reed program; Kericho, Nandi, Bomet and Narok and randomly selected facilities within this districts. Two HCWs from each facility were selected for three day training on IPC using DLTLD curriculum and later allowed to return and assess their department, using a standardized questionnaire. Two days were used to give feedback. Data analysis done using Epi-info and excel.

**Results:**

Eleven facilities were evaluated contributing 45 departments; comprehensive care, inpatient department, laboratory, maternal child health (MCH), outpatient department (OPD), and Tuberculosis (TB) clinic. Thirty three (73%) of departments had IPC departmental committees while 12 (26%) of the departments had TB IPC plans. Thirty one (69%) departments had at least one staff trained on TB IPC and 24 (53%) had continuous medical education (CME) on TB IPC in the last quarter. Nine (75%) department had assessed TB infection control plan within the last 1 month. Confidential HIV test to health care workers were done in 20 (44%) departments while 25 (56%) had posters on cough hygiene prominently displayed. Availability of IPC plan is associated with IPC risk assessment (OR = 49, CI = 5.32- 460.39, P< 0.001) and CME of HCWs in the last quarter (OR = 16.9, CI = 1.95-147.13, P < 0.001).

**Conclusion:**

Three quarter of the departments evaluated had IPC plans and had at least one staff trained. Availability of the plans is associated with increased IPC risk assessment and conducting CME. Tuberculosis IPC program implementation needs more strengthening. Training of HCWs improves capacity to implement TB IPC measures.

# Sub article: Evaluation of the Early Infant Diagnosis Program in Mazowe District, Zimbabwe, 2012

**Published:** 2015-07-22

**Authors:** Tendai Madziro-Nyagura

**Affiliations:** 1 Zimbabwe Field Epidemiology and Training Program

## Abstract

**Background:**

The main objective of Early Infant Diagnosis (EID) of HIV using DNA PCR is to identify HIV infected infants early enough to avoid morbidity and mortality through early initiation of antiretroviral therapy (EIT). Despite having implemented EID for the past two years, Mazowe District still had a low EID coverage of 10% whilst outcomes of HIV positive infants identified were also unknown. The study was carried out to evaluate the EID program in Mazowe District and to determine outcomes of the HIV positive infants identified through the process.

**Methods:**

A descriptive cross-sectional study was conducted amongst 29 health workers (HWs) in 13/26 health facilities in Mazoe District. We also conducted a retrospective cohort analysis on all EID records between January and December, 2011 to extract information on Dry Blood Spot (DBS) samples collected for DNA PCR and on outcomes of HIV positive infants. Information on program performance was collected using checklists.

**Results:**

All 29 HWs had received EID training. Of these, 27 (93.1%) could correctly describe the EID process. All health facilities in the study had DBS testing kits available and reported no stock out in the 6 months preceding the study. The mean duration between collection of DBS samples and the results being dispatched from the reference laboratory was 48 days. Of the 90 HIV positive infants identified in 2011, 62 (69%) had unknown outcomes whilst 17 (19%) were documented to have been initiated on antiretroviral therapy (ART), with a median duration between DBS sample collection and ART initiation of 19 weeks (Q1= 12; Q3 = 23). EID challenges reported by HWs included long turn-around times for DBS results (27/29), non-disclosure to male partners (16/29), long walking distances to clinics (13/29).

**Conclusion:**

Although the EID program has been in place for two years in Mazowe District, it is not meeting its main objective of EIT as only 19% of HIV positive infants were initiated on ART. In order to improve EIT we recommended community based follow-up of HIV exposed infants, scaling up male partner involvement, increasing geographical access to EID and use of mobile phone technology to notify DBS results.

# Sub article: Delays in Smear Positive Pulmonary Tuberculosis diagnosis in Harare city, Zimbabwe, 2012

**Published:** 2015-07-22

**Authors:** Tendai Madziro-Nyagura

**Affiliations:** 1 Zimbabwe Field Epidemiology and Training Program

## Abstract

**Background:**

In 2010, Harare city reported a low Tuberculosis (TB) notification rate, a high and increasing TB death rate. Literature reports that delay in TB diagnosis is one of the causes of low notification and high TB death rates. This study was carried out to quantify the delay to Pulmonary Tuberculosis (PTB) diagnosis as well as to identify factors associated with delayed PTB diagnosis in Harare.

**Methods:**

A cross sectional study using interviewer administered questionnaires was conducted at Beatrice Road and Wilkins Infectious Diseases hospitals. The study population was consenting newly diagnosed smear positive PTB patients aged at least 15 years and had been resident in Harare for at least 6 months prior to the onset of the illness. A cut off point of 4 weeks was used to define total delay in PTB diagnosis.

**Results:**

Of 190 participants enrolled, 139 (73.2%) had delayed PTB diagnosis. Median total delay was 6 weeks (Q1 = 4 weeks, Q3 = 10 weeks) of which patient delay contributed the greater proportion. The independent factors associated with total delay to PTB diagnosis were having 3 or more health care patient (HCP) encounters leading to diagnosis [AOR 2.66, 95% C.I (1.27-5.57)], first health provider consulted being a private practitioner [AOR 6.57, 95% C.I (1.86-23.24)], having a positive smoking history [AOR 2.78,95% C.I (1.1-7.04)] and belonging to an apostolic religious sect [AOR 4.09, 95% C.I (1.44 -11.64)].

**Conclusion:**

The high proportion of smear positive PTB patients delaying diagnosis in Harare implies continued transmission of TB in this community. Increased public awareness of TB signs and symptoms as well as enhancing public-private partnerships in TB control is recommended in order to reduce diagnostic delay in Harare. A similar study conducted in a rural Zimbabwean setting is also recommended in order to fill the knowledge gap in this regard.

# Sub article: Post Market Surveillance of HIV rapid test kits among testing facilities in Mwanza and Mara Regions, Tanzania – 2012

**Published:** 2015-07-22

**Authors:** Mura Ngoi

**Affiliations:** 1 Tanzania Field Epidemiology and Laboratory and Training Program

## Abstract

**Background:**

Introduction HIV pandemic continues to be a burden globally. Of 34 million persons infected in the world, 26 million live in Sub Sahara Africa with Tanzania having a prevalence of 5.1%. With the support of PEPFAR funding CDC has supported Tanzania in scaling up of HIV testing. This poses a greater challenge in testing hence forcing Tanzania Ministry of Health (MOH) to start PT program in order to monitor the quality of testing. This study aimed at determining the performance of different HIV rapid tests and factors that influence proficiency testing results.

**Methods:**

This was a Cross sectional study conducted in Lake Zone health facilities from November 2012-March 2013. Study settings were Laboratories, VCTs and PMTCTs enrolled in HIV EQA program. Data were collected by administering questionnaire; evaluation checklist was used to assess quality elements and EQA PT panels were provided to assess and compare performance across sites and among individuals. Data were entered, cleaned and analyzed using Epi-Info Version 3.5.1 and OR was used measure the association between testing requirements and EQA PT panels results.

**Results:**

General performance regarding HIV PT panels was 84.8% (89). Health workers who used SOP during testing were 3.7 times more likely to have given correct PT EQA results (OR = 3.7, 95% = 1.2, 11.1, P value = 0.02) while those who have never attended HIV training were less likely to give correct EQA PT results(OR0.2, 95%CI = 0.06, 0.53, Pvalue = 0.002). Other factors that influenced the performance of correct PT EQA results included Participation in EQA Programs, (OR = 3.84, 95%CI = 1.24, 11.9, P value = 0.023). Alere Determine and Uni-Gold had a concordance of 90% to 100% respectively when compared with reference kits, while SD Bioline HIV Ag/Ab Combo had Sensitivity of 98%, Specificity of 99.4%, PVP of 99.2% and PVN of 98.6% when evaluated against gold standard.

**Conclusion:**

Having not attended refresher training was associated with poor performance of Proficiency Testing. SD Bioline HIV Ag/Ab Combo Alere Determine and Uni-Gold kits had a sensitivity of above 95%. There is need to have regular refresher training for those who are involved in testing and revision of nation testing algorithm to include SD Bioline HIV Ag/Ab Combo.

# Sub article: Methicillin-Resistant Staphylococcus Aureus Colonization among Intensive Care Unit Patients and Health Care Workers at Muhimbili National Hospital, Dar es Salaam, Tanzania, 2013

**Published:** 2015-07-22

**Authors:** Alfred Geofrey

**Affiliations:** 1 Tanzania Field Epidemiology and Laboratory and Training Program

## Abstract

**Background:**

Methicillin-resistant Staphylococcus aureus (MRSA) has been recognized as important nosocomial pathogens worldwide. S aureus may induce clinically manifested diseases, or the host may remain completely asymptomatic. We conducted this study to determine the magnitude of MRSA colonization among intensive care unit (ICU) patients and health care workers at Muhimbili National Hospital (MNH), Dar es Salaam, Tanzania.

**Methods:**

A cross-sectional hospital-based study was conducted from October 2012 to March 2013 in two ICUs at MNH, Dar es Salaam, Tanzania. All admitted patients and health worker in ICU were interviewed using a standard questionnaires containing demographic and risk factors information. Nasal swab were taken from the study respondents and cultured using the conventional methods. Disk diffusion techniques were used to detect MRSA and determine antimicrobial susceptibility patterns of MRSA isolates. Data was entered, cleaned and analyzed using Epi-Info version 3.5.1. Univariate, bivariate and multivariate analysis was done where any factor with a P - value of < 0.05 was considered as statistically significant.

**Results:**

Of the 169 patients and 47 health workers who were recruited, the mean age was 43.4 years ± SD 15.3 and 37.7 years ± (SD) 11.44 respectively. Among the patients male contributed 108 (63.9%) while in health worker majority 39(83%) were females. The prevalence of MRSA colonization among patients and health workers was 11.83% and 2.1% respectively. All (21) MRSA isolates were resistant to penicillin and erythromycin, and high sensitive to vancomycin by 85.7%. Being male (Adjusted OR,6.74, 955CI 1.31-34.76), history of sickness in past year(Adjusted OR,4.89, 95% CI 1.82- 13.12), skin infections(adjusted OR 7.90,95% CI 2.16-29.17), diabetes(Adjusted OR 4.87, 95% CI 1.55-15.36) and use of drugs (Adjusted OR 10.18,95%CI 1.36-76.52) were found to independently associated with MRSA colonization.

**Conclusion:**

We identified a high prevalence of MRSA colonization among patients admitted in the ICU. Being male, history of sickness in the previous year, skin infections, use of drugs and diabetes were found to be independently associated with MRSA colonization among patients. Patients should be screened prior to ICU admission to identify the one carrying MRSA. Antibiotic sensitivity tests are recommended for effective treatment of MRSA.

# Sub article: Characteristics of People Living with HIV (PLHIV) diagnosed for Tuberculosis and treatment outcomes among HIV/AIDS patients attending TB clinic in Nigeria Institute of Medical Research, Lagos State, Nigeria

**Published:** 2015-07-22

**Authors:** Jamiyu Ganiyu

**Affiliations:** 1 Nigeria Field Epidemiology and Laboratory and Training Program

## Abstract

**Background:**

In 1993, Tuberculosis (TB) was declared as a global emergency and has remains one of the World's major causes of illness and death. In Nigeria, the disease is HIV-driven with a 25% TB-HIV co-infection rate. Misdiagnosis of TB among People Living with HIV (PLHIV) is a great impediment to reduction in morbidity and mortality. This study describes the characteristics of PLHIV diagnosed for TB and treatment outcomes among HIV/ AIDS patients attending TB clinic in Nigeria Institute of Medical Research (NIMR), Lagos State, Nigeria.

**Methods:**

A retrospective study was carried out on all patients (HIV-infected and non HIV infected), diagnosed and treated for TB between January 2006 to September 2012. Data on patients’ medical records which contained information on patients’ demographic characteristics, HIV status, clinical and treatment outcomes were reviewed and abstracted into excel spread sheet. Data were cleaned and descriptive; univariate and bivariate analysis were conducted.

**Results:**

Of the 4,339 TB cases reviewed, 3,459 (79.7%) were HIV-infected. Diagnosis of TB among HIV-infected was associated with older age (mean age 34.3 ±12.2 years, p < 0.001), being a female (OR = 1.72; CI 95%:1.48-2.01), having sputum negative result (OR = 4.28; CI 95%:3.63-5.04). PLHIV were more likely to achieve cure for TB (OR = 1.21; CI 95%:1.03-1.43) and less likely to develop failure to TB treatment (OR= 0.52; CI 95%:0.31-0.86).

**Conclusion:**

The study revealed that older age, being a female and having sputum negative results are characteristics associated with TB diagnosis among HIV patients. Achieving higher cure rate and lower failure rate was associated with HIV patients on TB treatment. This finding might be due to better adherence among HIV/AIDS patients, which might have been influenced by previous adherence counseling sessions before and when on anti-retroviral (ARV) drugs. Therefore, there is the need to strengthen and improve on adherence counseling among HIV negative TB patients by health care providers so as to achieve higher cure rate and minimize failure to Anti-TB drugs.

# Sub article: Prevalence and Risk Factors associated with HIV Transmission among Clients Accessing HIV Counseling and Testing (HCT) service in a Tertiary Health Facility in Lagos, Nigeria

**Published:** 2015-07-22

**Authors:** Jamiyu Ganiyu

**Affiliations:** 1 Nigeria Field Epidemiology and Laboratory and Training Program

## Abstract

**Background:**

The global estimate of people living with HIV/AIDS (PLWHA) and new infections was 34 and 2.5 million respectively in 2012. Nigeria ranks second among high burden countries with3.1 million PLWHA. The national coverage of HCT has remained low (1.5%). We conducted a study to determine prevalence and risk factors associated with HIV transmission among HCT clients in a tertiary health facility in Lagos Nigeria.

**Methods:**

We conducted a cross-sectional study on 633 clients who accessed HCT services in January 2013. We interviewed clients using intake forms to generate data on demographics, HIV risk behavior and practices. We used Serial HIV testing algorithm for HIV testing. We abstracted data into excel and carried out descriptive, univariate and bivariate analysis.

**Results:**

Of the 633 patients reviewed, 355(56.1%) were female and 306 (48.3%) were self-referred. The mean age was 31 (standard deviation: 13 years) with 68(10.7%) being children (<15 year). The overall HIV prevalence was 234 (37.0%) and varied from 17.6% among the children, 39.3% among the adults and 47.3% among females. HIV positive results in adults was associated with ever having a sexual intercourse (Prevalence Odds Ratio(POR) = 6.4; 95% Confidence Interval(CI):2.9-14.4), being a female (POR = 3.0; 95% CI: 2.0-4.3), being married (POR = 2.5; 95% CI: 1.8-3.7), unprotected sex with regular partner in the last 3 months (POR = 2.3; 95% CI: 1.7-3.3), having a Sexually Transmitted Infection(STI) in the last 3 months (POR = 2.6; 95% CI: 1.3-5.1), and blood transfusion in the last 3 months (POR = 2.8; 95% CI: 1.2-6.8).

**Conclusion:**

The prevalence among HCT clients in this tertiary facility is high with females having a significantly higher prevalence. Behavior al factors, sex practices, STI and blood transfusion may be responsible for the high HIV prevalence. The risk of transmission was higher among married couples which might have been influenced by marital sexual obligation as well as challenges of non-disclosure by infected spouse(s). Those who tested HIV positive were referred to treatment and care facilities while those negative were educated on risk reduction behavior. Government and stakeholders need to scale up prevention and behavior al change and communication activities in-country.

# Sub article: The prevalence of HIV and the Associated Factors in the fishing communities in Mwanza region, Tanzania-2012

**Published:** 2015-07-22

**Authors:** Simon Kabamanya

**Affiliations:** 1 Tanzania Field Epidemiology and Laboratory and Training Program

## Abstract

**Background:**

Fishing communities have been identified among the vulnerable groups at high risk for acquiring HIV. This vulnerability is mainly due to their mobility, time away from home, access to cash income and commercial sex at landing sites. The nature of their work contributes to low health service seeking behavior since much of their time is spent in water. Studies done in Uganda, Kenya, Cambodia and other parts of the world among the fishing communities reported higher prevalence of HIV compared to the general population. We conducted this study to determine the seroprevalence of HIV and the associated factors.

**Methods:**

This was a cross sectional community-based study that was conducted at Mwaloni landing site along the shores of Lake Victoria basin in Mwanza region in January 2013. Study respondents were interviewed using a standard questionnaire containing socio-demographic characteristics and risk factors information. Data was entered cleaned and analyzed using Epi-Info version 3.5.4.

**Results:**

A total of 448 individuals were included in the study. The mean age was 33.5 years (range 18-70 years). Many participants (61.2%) were males and (70.5%) had primary education. The prevalence of HIV was found to be 9.8%. Regular alcohol consumption was found to be a risk factor for acquiring HIV infection (OR = 4.4, 95%CI = 1.7-11.8). Study participants who had used the health services in the past one year were more likely to have HIV infection (OR = 3.8, 95%CI = 1.9-7.4). Results revealed a higher prevalence of HIV compared to the prevalence for the general population in Tanzania which is about 5.1%. About 129(29.3%) of the study participants reported to have utilized the health services in the last one year.

**Conclusion:**

The prevalence of HIV in the fishing community was found to be higher compared to the prevalence of HIV for the general population in Tanzania. Regular alcohol consumption was found to be a risk factor for acquiring HIV in the fishing community. Utilization of health of health services was found to be low. Efforts for addressing specific risk factors in fishing communities to reduce transmission of HIV are required. Regular mobile health services for prevention of HIV and treatment of opportunistic infections can help to minimize HIV transmission.

# Sub article: Implementation and usefulness of Fine Needle Aspirate and Cytology (FNAC) for Tuberculous Lymphadenitis diagnosis

**Published:** 2015-07-22

**Authors:** Uwimana Innocent

**Affiliations:** 1 Rwanda Field Epidemiology and Laboratory and Training Program

## Abstract

**Background:**

Tuberculous Lymphadenitis (TL) is a chronic specific granulomatous inflammation with caseation necrosis of the lymph node. Suspected Tuberculous lymphadenitis is difficult to diagnose and remains expensive when applying surgical procedures. We described and evaluated the usefulness of the fine needle aspirates and cytology (FNAC) to diagnose TL.

**Methods:**

Both clinicians and laboratory technicians were trained on how to collect FNAC samples and apply Papanicolaou and Ziehl Neelsen (ZN) staining techniques respectively. We collected lymph node fluid from suspected patients with palpable masses and triplicate slides smears were made. Slide #1, was stained by hot ZN method to identify acid-alcohol fast bacilli. Slide #2 was fixed in 95% alcohol and stained with Papanicolaou technique to identify Tuberculous Lymphadenitis features, malignant cells and other pathologies. Gram staining was performed on slide #3 to exclude any other bacterial infection.

**Results:**

Out of 120 medical personnel trained countrywide, 71 (59.1%) were clinicians or nurses and 49 (40.9%) were laboratory technicians. Training covered at least 59.0% (26/44) out of 44 District hospitals. From 138 FNACs samples collected, 83 (60.1%) were done from the neck (cervical area), 10 (7.2%) were done in the axillary region, 9 (6.5%) were from both cervical and axillary areas, 36 (26.1%) were done outside the axilla or neck which included inguinal, retro-auricular, forearm, anterior chest wall. Out of 138 patients, 10 (7.2%) were supportive for Tuberculous Lymphadenitis while 15 (10.9%) were suspicious for Tuberculous Lymphadenitis; there were no features for Tuberculosis in 113 (81.9%) patients. From all samples, only 52.8% (73/138) specimens had pus at the time of sample collection, from which Gram stain revealed 2 (1.4%) cases of Gram positive cocci and Gram negative bacilli. HIV sero-status showed that 40 (29.0%) patients were HIV positive, 44 (31.9%) patients were HIV negative. In the remaining 54 (39.1%) cases the determination of HIV status could not be ascertained.

**Conclusion:**

FNAC is safe, cheap and less traumatic compared to biopsy applying surgical procedures. The FNAC is useful technique since it gives more information from one single sample. It can be recommended in low income settings due to its minimal instrumentation requirements. Key words: Tuberculous lymphadenitis, fine needle aspirates and cytology, Papanicolaou technique and Ziehl Neelsen.

# Sub article: HIV prevalence and associated factors amongst prison inmates in Kuje federal prison, Federal Capital Territory, Abuja, Nigeria, 2013

**Published:** 2015-07-22

**Authors:** Adebobola Bashorun

**Affiliations:** 1 Nigeria Field Epidemiology and Laboratory and Training Program

## Abstract

**Background:**

HIV infection is of public-health importance. Worldwide, studies/service data have reported high morbidity amongst inmates due to diseases including HIV/AIDS. Gaps exist in the quantity/quality of information available on HIV/AIDS in Nigeria prisons and inmates are usually neglected in country-wide HIV plans. We conducted this study to determine HIV prevalence and associated factors amongst male prison inmates in Nigeria so as to guide prevention strategies and provide baseline information.

**Methods:**

We conducted a cross-sectional study with 154 inmates; we administered questionnaire; conducted Focus Group discussion; HIV testing using rapid test kits. Knowledge, attitude and practices variables were scored: one for correct response and zero for wrong, then graded over 10 [poor (0-4), fair (5-8), good (9-10)]. We used Epi-info V.3.5.3. for univariate, bi-variate and multi-variate analysis at 95% confidence interval, 0.05 significance with Chi^2^ test.

**Results:**

Mean age was 31.9 (+7.8) years. HIV prevalence was 4.0% (1.5-8.6%, 95% CI) with convicts: 3.3% and AT: 4.2%. 10.4% knew all the ways of HIV transmission. HIV knowledge mean score was 42% with 3 (1.9%) having good knowledge. 77.9% had positive attitude on HIV (mean score: 84%). 105 (68.1%) had ever been HIV tested. 15.6% used condoms consistently before incarceration. Based on quantitative, no (0%) heterosexual and male-male sex (MSM) practices; however, qualitative methods detected MSM. Proportions with tattoo, body piercing, injecting drug users (IDUs) were very low. HIV practices mean score was 61%, 0.0% had good practice. Age <25 years, prison stay <3 years, tattoo, body piercing were associated risks but not statistically significant while ever attended school, sexual transmission knowledge, condom use seemed protective but not significant. On Logistics regression, never heard of condom (AOR 33.1, P-value-0.016) and history of STI (AOR 18.4, P-value 0.05) were risks.

**Conclusion:**

HIV prevalence of 4% is above 1% reference for low level epidemics. Correct/complete knowledge and good practice on HIV were unsatisfactory. Never heard of condom and history of STI are independent predictors of HIV infection amongst Kuje prison inmates while various studies else identified tattoo, IDUs and MSM as risks. Advocacy and recommendations on behavioral change, STIs treatment, improving condoms accessibility and functional HIV testing services were made.

# Sub article: Factors associated with the uptake of mass drug administration for schistosomiasis control in Koome islands, Mukono district

**Published:** 2015-07-22

**Authors:** Doreen Tuhebwe

**Affiliations:** 1 Uganda Field Epidemiology and Training Program

## Abstract

**Background:**

Schistosomiasis is a neglected tropical disease targeted for elimination in Uganda and Mass Drug Administration (MDA) is one the strategies endorsed by the World Health Assembly for schistosomiasis elimination. In Koome Islands, an endemic area in Uganda, MDA is community directed using community resource persons in fixed sites and house-to-house visits however the uptake is still below target coverage on the islands and the reasons are not clear. This study determined the proportion of MDA uptake among community members in Koome Islands, and the factors associated with MDA Uptake.

**Methods:**

In March 2013, a cross sectional study was conducted in 15 randomly selected village camps in Koome. A total of 615 respondents aged 18 years old and above who had lived on Koome Islands for not less than 12 months participated using a structured questionnaire. Uptake estimates were calculated and factors assessed for association with swallowing schistosomiasis drugs were respondents’ predisposing, enabling and need factors like socio-demographics, knowledge and perceptions on schistosomiasis and MDA. Odds ratios were used to determine factors associated with MDA uptake.

**Results:**

It was estimated that 44.7% of the sampled population swallowed the schistosomiasis drugs during the 2012 MDA. Over half of the sampled population swallowed the drugs from their household and 55.5% of the respondents were knowledgeable about schistosomiasis causes, symptoms and prevention and MDA. Perceived need for MDA was associated with MDA uptake (OR= 3.37, 95% CI: 2.34-4.84) and fear of side effects was a significant barrier to MDA uptake (OR = 0.36, 95% CI: 0.25-0.54).

**Conclusion:**

The proportion of the sampled population that swallowed schistosomiasis drugs was much lower than the target coverage of 85%. The factors that were significantly associated with uptake of MDA were: length of stay in the village, fear of side effects, knowledge about MDA and perceived need of MDA. There is need to exert greater efforts to increase the people's knowledge on schistosomiasis and MDA and their understanding of perceived benefits of the drugs in order to increase uptake. These may contribute to the successful elimination of schistosomiasis on the Islands.

# Sub article: Malaria Outbreak Investigation in Pokot North District, Kenya- 2012

**Published:** 2015-07-22

**Authors:** Okunga Wandera

**Affiliations:** 1 Kenya Field Epidemiology and Laboratory and Training Program

## Abstract

**Background:**

Seasonal Malaria epidemics in Kenya have been characterized by high morbidity and mortality. In July 2012, an upsurge in Malaria cases beyond action thresholds from sentinel facilities in Pokot North District was detected. We conducted an investigation to characterize the outbreak by person, place and time.

**Methods:**

We reviewed clinical and laboratory registers at four sentinel surveillance sites in Pokot North District. A Malaria case was defined as patient with fever and at least two of the following symptoms: headache, backache, chills, sweats, myalgia, nausea or vomiting and a positive diagnostic test for Malaria presenting to a sentinel site between May and July 2012. EpiInfo^©^ was used for descriptive epidemiological analysis.

**Results:**

Of 1,538 patients with a clinical diagnosis of Malaria, 1,057 (69%) were tested and 827 (78%) had documented results. A total of 628 (76%) patients met the case definition; 321 (51%) had positive microscopy results and 307(49%) had positive rapid tests. The median age of case-patients was 7 years (interquartile range: 3-16 years), and 345 (54%) were female. Positivity rates increased across age groups from 72% in children <4 years, 78.5% in >4-9 years, 85% in >9-14 years, to 88% in >14-19 years before declining in adults >19 years to 69%. Overall test positivity rate for all case-patients was 76%. Only 62 (10%) case-patients were hospitalized while 566 (90%) were treated as outpatients. No deaths were reported. Two administrative divisions accounted for the majority of case-patients, Alale (47%) and Konyao (37%). The outbreak peaked the last 2 weeks in June 2012.

**Conclusion:**

Children were at greater risk of developing Malaria compared to adults; Malaria positivity rates were highest in older children and adolescents. These age-groups have not historically been targeted for Malaria prevention. Prevention efforts, including use of long-lasting insecticide-treated nets and educational messaging, should be focused on older children and adolescents.

# Sub article: Evidence of Circulation of Selected Arboviruses in Ijara and Marigat Districts, Kenya- 2013

**Published:** 2015-07-22

**Authors:** Victor Ofula

**Affiliations:** 1 Kenya Field Epidemiology and Laboratory and Training Program

## Abstract

**Background:**

Arboviruses are transmitted by arthropods with humans becoming infected during blood feeding by infected mosquitoes, ticks and sand flies. Characterization of arboviruses circulation and transmission has not been well documented in developing nations. The human involvement in the transmission cycle of these viruses has however not been demonstrated. This study sought to determine the sero-prevalence of a range of arboviruses in Districts where vector surveillance has been done.

**Methods:**

The study utilized samples from an ongoing study, whose main objective was to “to monitor circulation, transmission and maintenance of arboviruses with focus on Rift Valley Fever at hot spots in Kenya”. About 5µl of patients’ serum samples was used to test for each of the viruses selected. All the samples were tested by IgG ELISA; positive samples were confirmed using the Plaque Reduction Neutralization Test to determine the most likely causative agent.

**Results:**

A total of 351 patient serum samples were analyzed using IgG ELISA, of these 193 (54.9%) were male with age ranging between 3 and 73. The overall arboviruses prevalence was 53/351 (15%) with a prevalence of 7% (10/143) in Marigat and 21% (43/208) in Ijara. Of the positives, Flavi-viruses were 69%, alpha viruses 30% and Bunyaviridae 1.4%. Uganda S virus was the most prevalent with 10%, followed by West Nile virus 6%, Sindbis 5%, Dengue 2%, Semliki-forest virus-specific antibodies were detected by plaque reduction neutralization test in 3/351 (0.85%) persons tested. Antibodies against Sand fly Sicilian and Zika viruses were not detected.

**Conclusion:**

The study has demonstrated the circulation of the selected arboviruses in the two sites among human population. Preventive actions and awareness among clinicians in patient’ recognition and management should be enhanced.

# Sub article: Assessment of Malaria Control Interventions amongst high-risk groups attending health facilities - Oyo State, Nigeria, September 2011

**Published:** 2015-07-22

**Authors:** Elizabeth Adedire

**Affiliations:** 1 Nigeria Field Epidemiology and Laboratory and Training Program

## Abstract

**Background:**

Nigeria accounts for one-fourth of Malaria burden in Africa. Children less than 5 years (U5) and pregnant women (PW) are the high risk groups. Malaria is responsible for 30% of childhood mortality and 11% of maternal mortality. A three- prong approach to Malaria control comprising Artemisinin-based Combination Therapy (ACT) as first line treatment in all age groups except in first trimester of pregnancy, Intermittent Preventive Treatment in pregnancy using Sulfadoxine-pyrimethamine (IPTp) and use of Long Lasting Insecticidal Nets (LLINs) is practiced majorly in Nigeria. These commodities are available at health facilities and laboratory diagnosis (LD) is recommended prior to dispensing ACT. We evaluated the implementation of these Malaria control measures by health-care providers in Oyo State, Nigeria.

**Methods:**

We reviewed January-December 2011 Malaria surveillance data from the 564 health facilities of Oyo state, using the Monthly/ Quarterly Summary of Treatments and Preventive Services on Malaria and the Integrated Disease Surveillance and Response (IDSR) data for the State. We conducted descriptive analysis of clinical diagnosis, LD and patterns of Malaria presentation, treatments and preventive measures for U5 and PW using Microsoft excel 2007.

**Results:**

A total of 435,050 patients were suspected to have Malaria accounting for 71.2% of patients in the health facilities, highest incidence of Malaria was seen in month of June to September with a peak in July 2011. Of the 435,050 patient with suspected Malaria, there were 223,411 (64.5%) febrile U5 children. Of these febrile U5 children, 220 (0.6%) had severe Malaria, 1,322 (0.6%) had LD, and 84,673 (37.9%) received ACT; Of the 147,557 women who attended ante-natal clinic, 82,006 (55.6%) had at least one dose of IPT and 13,369 (9.0%) received LLIN.

**Conclusion:**

Laboratory confirmation of Malaria is rarely practiced. Use of ACT for Malaria treatment in U5 and distribution of LLIN to pregnant women attending health facilities is low. Utilization of IPT for prevention of Malaria in pregnancy is far from the target of 100%. There is a need to strengthen LD, and we recommend the training and sensitization of health workers to improve utilization of Malaria control measures. Keywords: Malaria control, Pregnant Women, Under-Fives, Artemisinin-based Combination Therapy, Intermittent preventive treatment in pregnancy, Long Lasting Insecticidal Nets.

# Sub article: Risk Factors for Death from Severe Malaria among children aged Under Five Years in Rwanda, 2010-2011

**Published:** 2015-07-22

**Authors:** Alphonse Rukundo

**Affiliations:** 1 Rwanda Field Epidemiology and Laboratory and Training Program

## Abstract

**Background:**

Malaria is a major public health concern in Rwanda with Malaria mortality at health facilities for children aged under five years estimated at 13% of total deaths in 2010. The objective of this study was to identify potential risks factors that may contribute to Malaria deaths so that appropriate interventions can be initiated.

**Methods:**

Between October and December 2011, we conducted a retrospective case-control study covering a period of 18 months from January 2010 to June 2011, in nine District hospitals in Rwanda. Cases (n = 95) were children aged under five years hospitalized for and dying of confirmed severe Malaria. The control subjects (n = 95), matched for age, sex and date of hospitalization; were children hospitalized for confirmed severe Malaria who were discharged after recovery. Risk factors assessed included self-medication, promptness of hospitalization, clinical and para-clinical signs. Odds ratios (OR) and 95% confidence intervals (CI) for the potential risk factors for death from Malaria were calculated.

**Results:**

We enrolled 95 cases and 95 controls. Overall, 49 (51.6%) were girls and 46 (48.4%) boys. The average age of participants was 24 months (range: 6-59 months) and the average hospitalization among cases was 1.4 days (range: 0-9 days). The following factors were identified to be associated with a significant increase in risk of death from Malaria: late seeking of treatment (OR = 17.7 95%CI: 8.5-36.7), high parasitaemia (OR = 6.5 95%CI: 2.5-16.6) and chronic malnutrition (OR = 3.9 95%CI: 2.8-5.0). In contrast the Malaria deaths were not statistically significantly associated with self-medication (OR = 1.3 95%CI: 0.6-2.1), form of severe Malaria (OR = 1.2 95%CI: 0.7- 2.2), age group of the children (OR = 1.2 95% CI: 0.7-2.2) or fever of 38° C or higher (OR = 1.5 95%CI: 0.7-2.8).

**Conclusion:**

Delayed care-seeking, high parasitemia and malnutrition were identified as risk factors for Malaria deaths. There is need for more health sensitization to encourage early care-seeking for fever, community-based interventions, and short-term education for mothers in order to increase their knowledge on nutrition needs.

# Sub article: Knowledge and Prevalence of Human African Trypanosomiasis among Residents Kachia Grazing Reserve - Nigeria, 2012

**Published:** 2015-07-22

**Authors:** Belinda Uba

**Affiliations:** 1 Nigeria Field Epidemiology and Laboratory and Training Program

## Abstract

**Background:**

Human African Trypanosomiasis (HAT) is a vector borne parasitic disease transmitted to humans by bites of infected tsetse flies. Human infections cause significant morbidity and mortality including delayed child mental development. In 2012 a suspected outbreak of a HAT outbreak was reported in Kachia Grazing reserve. We set out to investigate the outbreak, determine the prevalence and knowledge of HAT among residents of the grazing reserve.

**Methods:**

We conducted an active community and health facility search of HAT cases. We conducted a cross-sectional descriptive study using multi-stage sampling with probability proportionate to size. Respondents were administered structured questionnaire on Knowledge, practices relating to HAT prevention and screened for HAT using card agglutination test for Trypanosomiasis (CATT). Knowledge of HAT was scored into 5 domains and categorized as poor knowledge and satisfactory based on the mean knowledge score. Predisposition to risk of infection was defined as frequent exposure to ≥two known risk factors for HAT. A case of HAT was defined as any respondent that tested positive on CATT. We analyzed data using Epi-info and MS-excel.

**Results:**

There were no active cases in the community or the health facilities. Of the 300 respondents, mean age 39 (±17years) interviewed, 56.3% were males. Only 12.0% had good knowledge of HAT and 76.3% were exposed to HAT risk factors. HAT prevention practices included clearing of overgrown bushes around houses (99%), use of insecticidal treated nets (75.7%) and protective clothing (41.0%). Being a Male {Odds Ratio (OR) 5.0; 95% Confidence Interval (CI) 1.8 - 13.6} older than 40 years (OR 5.0; 95% CI 1.1 - 24.4) and having a family history of HAT (OR 8.7; 95% CI 2.4, - 32.1) were statistically significantly associated with HAT knowledge. None of the respondents tested positive on CATT screening.

**Conclusion:**

Knowledge of HAT was poor while practices relating to HAT prevention were common among residents. A zero prevalence of HAT was recorded.

# Sub article: Human Rabies Outbreak - Cross River State, Nigeria, September, 2012 - the role of canine vaccination and Post Exposure Prophylaxis

**Published:** 2015-07-22

**Authors:** Charles Michael

**Affiliations:** 1 Nigeria Field Epidemiology and Laboratory and Training Program

## Abstract

**Background:**

Rabies is an acute, highly fatal, viral zoonosis caused by Rabies virus. It causes 55,000 annual human deaths globally. 22400 (44%) of these deaths occur in Africa with 10000(18%) recorded in Nigeria. Cross River State, Nigeria, reported human deaths from Rabies infection between June and August, 2012. All victims had a history of dog bite and demonstrated signs of hydrophobia, aerophobia, convulsion/ spasms, excessive salivation and difficulty swallowing prior to death. We investigated the outbreak to identify factors associated with Rabies among dog bite victims.

**Methods:**

We identified dog bite victims and the status of biting dogs by reviewing hospital and State Veterinary Clinic (SVC) records from March-September, 2012. We followed up dog-bite patients and where deceased, family members, to collect data on associated factors using structured questionnaire. We conducted Focus Group Discussions (FGD) with physicians at the hospitals on dog bite patient management.

**Results:**

At the SVC 16 dog bite incidences were recorded, seven (43.7%) dogs were presented for clinical examination, four dogs (25%) were killed, and the status of five dogs (31.3%) was unknown. Only two dogs (12.5%) were vaccinated. Eight out of 90 dog-bite patients presented at the hospitals with neurologic signs suggestive of Rabies and were clinically diagnosed with Rabies. All eight patients died. Median age of dog-bite victims was 25 years. Eight four bitten individuals (94%) did not receive post-exposure prophylaxis (PEP). In 17 out of 24 (70.8%) followed-up patients, bites were unprovoked, 21 (87.5%) of the respondents lacked knowledge of Rabies.

**Conclusion:**

Poor knowledge about Rabies, improper management of dog bites victims, non-availability of PEP, and preponderance of unvaccinated dogs were identified as associated factors for the outbreak. The lack of PEP and standard treatment protocols resulted in poor case management. We linked the hospitals with sources of PEP and advised them to administer it to all subsequent victims of dog bite. We educated the public on Rabies via television and radio stations in the state. The State Ministry of Agriculture was asked to vaccinate dogs.

# Sub article: Human and Animal Rabies in Namibia, 2007-2012

**Published:** 2015-07-22

**Authors:** Emmanuel Hikufe

**Affiliations:** 1 Namibia Field Epidemiology and Laboratory and Training Program

## Abstract

**Background:**

Rabies is a highly fatal zoonotic disease caused by a virus of the family Rhabdoviridae. Globally, Rabies is responsible for 55,000 human and millions of animal deaths annually. The disease is common in Namibia resulting in loss of life and income. Although data on Rabies is routinely collected, analysis is not done. We conducted this study to determine the disease trend in human and animal and to estimate income loss due to Rabies.

**Methods:**

We conducted a retrospective descriptive study. We reviewed veterinary data on animal Rabies from 2007 to 2012 and the Ministry of Health (MOH) data on human Rabies from 2008 to 2011 and analyzed the data in terms of person, place and time. We estimated the income lost due to death of bovines and kudus by multiplying the average price of the particular animal by the number of deaths.

**Results:**

From 2007 to 2012, a total of 1,703 animals comprising bovines 523 (30.7%), dogs 521(30.6%), kudus 409(24.0%) and other animals 250(14.7%) died of Rabies in Namibia. Animal deaths in 2007, 2008, 2009, 2010, 2011 and 2012 were 189(11.1%), 120 (7.0%), 221 (13.0%), 472 (27.7%), 374 (22.0%) and 327(19.2%) respectively. Income lost due to bovine and kudus deaths from Rabies were $1,625,000 and $1,022,500 respectively. A total of 1,363(80%) animal deaths occurred in the northern sector of the country. From 2008 to 2012, there were 62 Rabies related human deaths comprising 37(60.0%) males. The number of human deaths in 2008, 2009, 2010 and 2011 were 13(21%), 14(22.6%), 17(27.4%) and 18(29.0%) respectively. Fifty two (84%) deaths occurred in children less than 16 years old. Fifty six deaths (90.3%) occurred in the northern sector of the country.

**Conclusion:**

Rabies is a significant cause of death in animals resulting in huge income losses. Human Rabies is increasing in trend. Majority of human deaths are children. Rabies is more common in the northern sector of the country. We recommended joint control efforts by the veterinary service and MOH, mass animal vaccination, pre-exposure prophylaxis for children in the northern sector of the country, intensified surveillance and health education.

# Sub article: Preparedness against Cholera Epidemic among local health authorities in Oyo State, Nigeria

**Published:** 2015-07-22

**Authors:** Eze Emmanuel Ikechukwu

**Affiliations:** 1 Nigeria Field Epidemiology and Laboratory and Training Program

## Abstract

**Background:**

An evaluation of the state of preparedness is therefore required to determine gaps and proffer solution to strengthening epidemic response in the State. This study is therefore aimed at assessing the level of preparedness to respond to Cholera epidemic by local health Authorities in Oyo State.

**Methods:**

The study is descriptive cross-sectional in design. A self-administered questionnaire was used to collect data on availability of surveillance logistics and supply from Disease Surveillance and Notification Officers (DSNOs) (34), Monitoring and Evaluating officers (M/Es) (32), Primary Health Care Coordinators (PHCCs) (33) and epidemiologist (1) in Oyo State health service. A seven-point knowledge scale was used to assess knowledge of Cholera case definition. A nineteen-item preparedness scale which included checklist on surveillance practice and outbreak response strategies was used to assess the level of preparedness. Descriptive statistics and chi-square test were used to analyze data.

**Results:**

Less than half (49.0%) knew the number of cases that constitutes an epidemic, although 83.0% knew the symptoms of Cholera. More DSNOs (70.6%) had adequate knowledge of Cholera case definition compared with the PHCCs (64.7%) and M/Es (21.9%) (p = 0.019). A little above half (53.0%) of respondents had adequat knowledge of Cholera case definition. Only one (3.0%) of the thirty-three LGAs had supplies reserved for outbreak response. Though 78.8% of the LGAs had the capacity to transport specimen to laboratory for confirmation, only 36.4% had specimen bottles. The logistic support available include surveillance office (81.8%), transportation (90.9%), health personnel (33.3%), imprest (33.3%) and survey forms (100%). Only 21% of the epidemic preparedness team meets regularly. While the adequacy of the LGAs in areas of surveillance and logistics were 66.7% and 60.6% respectively.

**Conclusion:**

There is need to improve knowledge concerning Cholera case definition especially among M/Es and strengthen epidemic preparedness in the state in areas of supplies and funds.

# Sub article: Diagnosis of Epidemic Typhus in a Youth Rehabilitation Center in Rwanda, 2012

**Published:** 2015-07-22

**Authors:** Francois Habiyaremye

**Affiliations:** 1 Rwanda Field Epidemiology and Laboratory and Training Program

## Abstract

**Background:**

In August 2012, the Rwanda Ministry of Health received notification of an unknown febrile disease in a male youth rehabilitation center in Eastern Rwanda. Investigations were undertaken to confirm the diagnosis, and guide treatment and control measures.

**Methods:**

A suspected case was defined as any camp resident presenting with one or more of the following symptoms: fever and/or joint-muscle; and/or abdominal; and/or thoracic pain. Clinical chemistry and Malaria tests were conducted by the admitting hospital laboratory. Whole blood and/or serum samples (acute and convalescent) were collected from 21 hospitalized patients and sent to CDC reference laboratories to test for multiple pathogens (including Arboviruses, Brucella, Bartonella, Leptospira and Rickettsia) using serology, molecular tests and indirect immunofluorescence assays (IFA). Samples were considered confirmed if positive for the specific pathogen by real-time PCR, DNA sequencing of nested PCR products, or by four-fold change in IgG titers by IFA in paired sera. A probable diagnosis was considered an IFA titer ≥ 1:64 for *R prowazekii*, or ≥ 1:256 for *B. quintana*.

**Results:**

During the period, April-August, 199 patients (attack rate: 10.4%) were identified in the center which housed 1,910 male residents; median patient age was 25 years (range: 11-45). Seven deaths (CFR: 3.5%) occurred within 48 hours of hospital admission. Initial laboratory results mostly showed Hypoglycaemia, and high urea, Creatinine and Transaminase levels, and raised erythrocyte sedimentation rates. All were negative for Malaria. Molecular and IFA test results confirmed epidemic typhus as well as co-circulating *Bartonella quintana*. Out of the 21 patients tested, four (19%) were confirmed *Rickettsia prowazekii*, three (14%) as mixed *R. prowazekii* and *Bartonella quintana*, one (5%) confirmed *R. prowazekii* and probable Bartonella group, eight (38%) probable typhus and probable Bartonella group, three (14%) probable Bartonella group, and one (5%) probable typhus group, while one (5%) was negative. Other tests for arboviruses, leptospirosis, brucellosis and typhoid were negative.

**Conclusion:**

The detection of epidemic typhus and bartonellosis in this outbreak highlights the significance of these diseases as causes of acute febrile illnesses in Rwanda. Doxycycline was recommended for treatment of patients and delousing and improved hygiene and sanitation recommended as prevention and control measures.

# Sub article: Meningococcal Meningitis Outbreak Investigation- Southern Nations Nationalities Peoples Region, February 2013

**Published:** 2015-07-22

**Authors:** Omod Opeeno

**Affiliations:** 1 Ethiopia Field Epidemiology and Training Program

## Abstract

**Background:**

Meningitis is an acute infection of the meninges. Meningitis caused by *Neisseria meningitidis* is a major health problem in African meningitis belt where highest incidences of a disease are observed and annual recurrent epidemics occur during the very hot, dry season most frequently caused by serogroup A. In January 2013 Gamo Gofa Zone of South Nations Nationalities Peoples Region reported an increased number of suspected meningitis cases. We investigated to identify the agent, evaluate the risk factors and offer recommendations.

**Methods:**

We defined a suspected case of meningitis as the occurrence of sudden onset of fever (>38.0oC rectal or >38.0oC axillary) and one of the following: neck stiffness, bulging fontanel, convulsion or meningeal signs among residents of Arbaminch Hospital, Lante Health Center or Channo Mille Health Post during January to February, 2013. We described the outbreak by time place and person. We conducted a case control study and compared risk factors in cases with neighborhood unmatched controls using Epi-Info 7.

**Results:**

We identified 46 cases and 7 deaths (AR 24/100,000 populations, highest among persons15-29 years of age.) The outbreak occurred from 12-January to 21-February, 2013. *N. meningitidis* serotype C was detected by rapid diagnostic test from one of the four cerebrospinal fluid specimens. Among 32 cases and 64 controls, people with cases within their household or neighborhood were 3.2 times more likely to develop meningococcal meningitis (OR =3.2, 95% CI= 1.3-8.2). People with upper respiratory tract infection in the previous two weeks (OR = 4.6, CI = 1.6-12.7) were 4.6 times more likely to develop disease.

**Conclusion:**

This investigation confirmed an outbreak of meningococcal meningitis and suggested that a case within the household or neighborhood and upper respiratory tract infection in the previous two weeks were risk factors for contracting disease. One case was confirmed to be caused by serogroup C. We recommended active case search, community education, case treatment and a mass vaccination campaign. Further studies are needed to determine the prevalent serogroups of meningococcal meningitis during outbreaks in Ethiopia to guide effective responses.

# Sub article: Outbreak of Measles among Farmers Children, Gwadabawa Local government Area, Sokoto State, Nigeria – 2012

**Published:** 2015-07-22

**Authors:** Binta Bako

**Affiliations:** 1 Nigeria Field Epidemiology and Laboratory and Training Program

## Abstract

**Background:**

Measles is highly infectious vaccine preventable disease, associated with high morbidity and mortality is the fifth leading cause of death among children younger than 5 years in Nigeria. Northern Nigeria suffers recurrent outbreaks due to low immunization intake. We investigated a suspected Measles outbreak in Gwadabawa District to identify risk factors and institute control measures.

**Methods:**

An unmatched Case-Control study was conducted. A case was a person less than five years residing in Gwadabawa presenting with fever, generalized maculopapular rash, plus any of the followings, Fever, Cough, Conjunctivitis, or Coryza between 1 June and 1 July 2012, neighborhood controls were used. We administered questionnaires to 63 cases and 63 controls to obtain information on risk factors, socio-demographic characteristics, and immunization status. Fifteen Blood samples were collected for Measles serology.

**Results:**

The mean age was 25 Months (±SD of 14.4). Nine samples tested positive to Measles IgM. Being vaccinated with one dose of Measles and having a national Vaccination card was found to be protective (OR = 0.0182, 95% CI: [0.009-0.3532] OR = 0.0067 CI: 0.0059-0.7550] respectively. Measles infection was associated with a history of contact with a known measles case (OR = 43, 95% have Measles (95%CI [1.44-69.26]) Diarrhea and pneumonia were less common (95%CI [12.26-151.87]). Children living 5Km within a Health facility were 10 times more likely to present complications reported after Measles 77% and 37% respectively. Among persons interviewed Measles vaccine uptake was 36%. There were no statistical significant relationships between Measles disease, Household income, history of recent travel and overcrowding.

**Conclusion:**

Measles outbreak was confirmed and risk factors included contact with Measles case, lack of Vaccination, Reactive Vaccination campaign, prompt case management and Health promotion activities were instituted. Local government should strengthened routine immunization and active case based surveillance.

# Sub article: Meningococcal Meningitis Outbreak of Sero-group A with emergence of Sporadic Serogroup W135, Ethiopia- 2013

**Published:** 2015-07-22

**Authors:** Taame Haftom

**Affiliations:** 1 Ethiopia Field Epidemiology and Training Program

## Abstract

**Background:**

Meningococcal meningitis has been a major cause of morbidity and mortality in the “meningitis belt,” which extends from Senegal to Ethiopia. Historically most cases of meningococcal meningitis in this region have been caused by serotype A, and a novel vaccination program for serotype A is being implemented for the 26 countries in the meningitis belt. The emergence of serogroup W135, which was responsible for a large meningitis epidemic in Burkina Faso, may alter vaccination policy. In February 2013 an increased number of meningitis cases were reported from the Dale and Wensho Districts of Ethiopia. We investigated to confirm the outbreak, identify risk factors and meningococcal serogroups, and implement control measures.

**Methods:**

A suspected case of meningococcal meningitis was defined as a person with a sudden onset of fever and stiff neck. A probable case was a suspected case who had turbid cerebrospinal fluid (CSF) and a confirmed case was a suspected case who had *Neisseria meningitidis* detected from CSF by culture or antigen detection (PCR) from January 17- March 5, 2013. We conducted a case control study with 32 cases and 58 age-matched controls and performed a multivariate analysis.

**Results:**

We identified 32 suspected meningococcal meningitis cases, of which 10 were probable cases, and 12 were confirmed cases. Among the 12 confirmed cases, we identified 9 cases with serogroup A and 3 cases with serogroup W135. There was one death. All cases were unvaccinated. The attack rate (AR) was highest in children aged 5-14 years (AR = 17/100,000) followed by persons aged 15-44 years (AR = 9/100,000). Literacy (odds ratio [OR]:0.2, 95% CI: 0.1-0.5) was protective and travel history to meningitis epidemic areas (OR: 11.0, CI 1.9-65.3) was a risk factor for meningococcal meningitis.

**Conclusion:**

We confirmed a *Meningococcal meningitis* outbreak caused predominantly by serogroup A in southern Ethiopia. Cases of serogroup W135 were identified for only the second time in Ethiopia. During this outbreak we strengthened the diagnostic capacity of public health laboratories by introducing Real-Time PCR. We recommend strengthening the surveillance program for meningococcal serogroups to help guide future responses and vaccination programs.

# Sub article: Measles Outbreak investigation- Gesha District, Keffa Zone of Southern Ethiopia, 2012

**Published:** 2015-07-22

**Authors:** Mekdes Demissie

**Affiliations:** 1 Ethiopia Field Epidemiology and Training Program

## Abstract

**Background:**

Measles is one of priority public health problem in Ethiopia. More than 5,000 cases have been reported since the beginning of 2011 and number of children at risk of Measles was estimated to be 2 million. In October 2011, we received a report of unusual increase of Measles cases in Gesha District of South Western Ethiopia. We investigated to confirm the outbreak, identify risk factors, and implement control measures.

**Methods:**

Suspected case of Measles was defined as any person with fever and maculopapular rash with one of the following signs cough, coryza or conjunctivitis while a confirmed case was a suspected case with positive laboratory test for Measles IgM or epidemiologically linked with confirmed cases. Unmatched case-control study was conducted, and odds ratios with 95% confidence intervals were calculated to compare risks of exposure among cases and controls.

**Results:**

We identified 79 confirmed cases of which 5-14 years of age constitute 48% (38/79). The outbreak started in September, 2012 peaked twice until first week of February 2013. Case patients were clustered around thirteen villages with overall attack rate and case fatality rate of 2.5% and 0.04% respectively. Five cases were laboratory confirmed for Measles IgM and the remaining cases were epidemiologically linked. Children who were not vaccinated were found to be more likely to contract Measles (OR = 11.06 [95% CI. 5.8-21.5]) than vaccinated, and children whose mothers don't know about Measles vaccination were also found to be more likely to develop Measles (OR= 5 [95% CI. 2.7-8.9].

**Conclusion:**

Laboratory confirmed Measles outbreak has occurred in Gesha District of Southern Ethiopia. Accumulation of unvaccinated older children and lack of awareness about Measles vaccination might have caused the outbreak. We recommend Supplementary Immunization campaign and education of mothers about Measles vaccination.

# Sub article: Outbreak of Cerebrospinal Meningitis in Gwandu Local Government, Kebbi State - Nigeria, 2013

**Published:** 2015-07-22

**Authors:** Joseph Rikoto

**Affiliations:** 1 Nigeria Field Epidemiology and Laboratory and Training Program

## Abstract

**Background:**

Cerebrospinal meningitis (CSM) caused by bacteria is a serious threat to global health. Globally, over 1.2 million cases and 170, 000 deaths due to bacterial meningitis are estimated to occur and is commonly caused by Haemophilus Influenza e, Streptococcus pneumoniae and Neisseria meningitides. In February 2013, an outbreak of CSM was reported in Kambazar-Fulani Village Gwandu LGA Kebbi State, North-western Nigeria. We investigated the outbreak to identify the causative agent, source of infection and possible risk factors associated with the outbreak.

**Methods:**

We collected cerebrospinal fluid (CSF) samples from suspected cases and analyzed using Pastorex TM Meningitis to identify the pathogen. We defined a suspected case of CSM as any person with sudden onset of fever, headache, neck stiffness, altered consciousness and any other meningeal signs in Kambazar-Fulani between February and March 2013., and a confirmed case as any suspected case confirmed by identification of the causal pathogen from cerebrospinal fluid (CSF) by agglutination test. We recruited 28 cases and 28 controls in a case-control study. We administered a structured questionnaire to obtain demographic information, history of travel and contact with a suspected case. We performed descriptive and bivariate analysis to describe the data in person, place and time and calculated odds ratio for exposure factors at a significant level of < 5%.

**Results:**

A total of 28 cases were identified with 8 deaths (case fatality rate: 28.6%). (Median age of cases was 9 years (range 9 months to 40 years). Half (50%) of cases were males. The most affected age group was <1 -10 years with 15 (53.6%) cases. Contact with a suspected CSM case was found to be associated with the infection (Odds Ratio 5.4, 95% Confidence Interval 1.7-17.1). *Haemophilus influenza* type B was isolated from 2 samples and Streptococcus pneumoniae type B from 1 CSF sample.

**Conclusion:**

An outbreak of CSM occurred in Kambazar-Fulani Village. We recommended the immediate provision of Ceftriaxone or oily chloramphenicol for control. We also recommended mass vaccination with conjugated polysaccharide vaccines and intensified CSM surveillance for prevention. The State Ministry of Health immediately instituted control measures.

# Sub article: Evaluation of Malaria surveillance system in Oyo state, Southwestern Nigeria January 2013

**Published:** 2015-07-22

**Authors:** Adedire Elizabeth

**Affiliations:** 1 Nigeria Field Epidemiology and Laboratory and Training Program

## Abstract

**Background:**

Nigeria accounts for one-fourth of Malaria morbidity in sub-Saharan Africa; Oyo State is holoendemic for Malaria and is one of the eight states targeted for Malaria control by the United States President Malaria initiative (PMI). Surveillance data are used to monitor trends and prioritize allocation of insecticide treated net distribution and Artemisinin based combination therapy treatments. In 1998, the national Malaria surveillance system was set up by the Federal government to support prevention, treatment and control efforts. We evaluated the Malaria surveillance system in Oyo state to assess its attributes and performance in line with set objectives.

**Methods:**

We assessed the surveillance system according to the Center for Disease Control and Prevention guidelines for evaluating public health surveillance system (2001). We quantitatively and qualitatively assessed the surveillance system's key attributes. We interviewed 7 key informants (KI) at state level and administered semi-structured questionnaire to all 33 Local Government Area (LGA) Malaria focal persons. We reviewed surveillance data from 2008-2012.

**Results:**

Case definition and tools are simple and are acceptable to key stakeholders. Data flow from the local Government (LGA) to the State is clearly defined but feed-back is irregular. Data quality is poor and surveillance data is collected mainly from public health facilities hence not representative. Late reporting is observed in 17 of 33 LGA (55.5%). Of the 7 KI, 5 (71%) agreed the system collects relevant data for the description of the epidemiological situation. There has been decreasing trend in Malaria morbidity, the prevalence of Malaria reduced by half in 2012 compared to 2010 (465116 cases versus 273094 cases). Partner agencies provide 95.3% of system's operating resources.

**Conclusion:**

The non-representativeness of the system increases the potential for incomplete reporting of the disease which may affect control efforts. There is therefore a need to focus effort on capturing excluded facilities in the surveillance system. There is a need to provide supportive supervision for data quality assurance to improve data quality and timeliness.

# Sub article: Progress towards Polio Eradication: Lagos State, 2001-2012

**Published:** 2015-07-22

**Authors:** Abimbola Aman-Oloniyo

**Affiliations:** 1 Nigeria Field Epidemiology and Laboratory and Training Program

## Abstract

**Background:**

Poliomyelitis is a crippling disease that affects mostly children. In 1988, the World Health Assembly adopted a resolution to eradicate polio. Nigeria is a signatory to this resolution and has had a marked reduction in annual Polio case detection in the last 10 years. Lagos State is one of the largest States in Nigeria with an area of 3345 sq. Km and population at 9,113,605 (Census 2006). 40% of the population is < 15 years old. The population density of 2,594 persons per sq. Kilometre increases the risk of spread of communicable diseases. We analyzed polio state data for 2001-2012.

**Methods:**

Annual data on Acute Flaccid Paralysis (AFP) surveillance and poliomyelitis case detection from the state were reviewed over a 12-year period.

**Results:**

Annual number of AFP from 2003 to 2012 has ranged between 222 and 357 with an average of 269.9 cases. This overall AFP detection rate consistently met the WHO requirement of 2/100,000 of population <15 years. Number of Wild Polio Virus Cases in Lagos State since 2001 has ranged between 0 and 12. The highest number of cases was seen in year 2003 (12). No human case was seen in 2002 and from 2010 to date. The last human case in Lagos State was in 2009 when 4 cases were detected. The age range of these 4 cases were1 yrs. 6 months - 1 Yrs. 10 months, more males (75%) were affected than females. Three cases (75%) had no evidence of receiving routine immunization (immunization cards). No case of polio was detected in 2002, 2006, 2010 and 2011. Prior to 2011, there appeared to be a cyclical pattern of “zero case” every 4 years. An environmental polio case was detected in 2012.

**Conclusion:**

The poliomyelitis eradication efforts in Lagos State have yielded positive results as the last human case detected was in 2009. The Polio Eradication Initiative (PEI) efforts however need to be consolidated to avoid a resurgence of cases in subsequent years.

# Sub article: Evaluation of the National Buruli Ulcer Surveillance System-Ghana, 2012

**Published:** 2015-07-22

**Authors:** George Kuma

**Affiliations:** 1 Ghana Field Epidemiology and Laboratory and Training Program

## Abstract

**Background:**

Buruli ulcer (BU) is an infectious chronic skin disease caused by *Mycobacterium ulcerans*, characterized by a painless nodule, papule, plaque or edema, progressing into a painless ulcer with undermined edges. It affects all age-groups, especially <15years. BU if not promptly diagnosed and treated leads to irreversible deformities rendering individuals unable to work and sometimes branded social outcasts. Of 170 Districts in Ghana, 58 are known to be endemic and about 16-22% of the population in some communities have been affected. Continuous surveillance and periodic evaluation is required to monitor BU trends, assess sensitivity of the system, disease burden and drive control policy, strategies and resource prioritization in managing the disease. We evaluated the BU surveillance system to determine if these objectives are being met and to assess its attributes and usefulness.

**Methods:**

Using an adapted CDC updated Guidelines for Public Health Surveillance Evaluation (2001), we conducted a cross-sectional survey from 18th December- 2011to 31st January-2012 in which we derived information from stakeholders and key informants. Surveillance records and data-set from 2006-2011 at the national, regional and District levels were reviewed and analyzed descriptively in Epi-info 3.3.5.

**Results:**

Cumulative cases for 2006-2011 was 5540. The system was sensitive in identifying a total of 971 cases in 2011. Early case detection rate assessed by Non-ulcerative: Ulcerative ratio was1:3.5 (22.4%). Categorized age-groups were 0-15, 16-49 and ≥ 50years with 15-49years as the predominant Sub-group affected: 392/946 (41.4%). The proportion of cases successfully treated to prevent disabilities was 721/925 (77.9%). It was flexible, acceptable, stable and fairly simple. Data quality was 97%. Proportion of cases confirmed by laboratory was ∼66%. Information derived from stakeholders and personal observations showed that data from the system was being used in prioritization and resource allocation.

**Conclusion:**

BU trends in Ghana show that 15-49yrs are most affected. The system is useful and meeting its objectives. However, we recommend District laboratories to perform more Ziehl Neelseen test on BU samples to improve laboratory confirmation rate which together with enhanced surveillance, will be the key to increasing significantly, early detection rate and management, hence reducing disabilities.

# Sub article: Evaluation of the Poliomyelitis and Acute Flaccid Paralysis surveillance (AFP) system in Sierra Leone, 2012

**Published:** 2015-07-22

**Authors:** Dauda Sowa

**Affiliations:** 1 Ghana Field Epidemiology and Laboratory and Training Program

## Abstract

**Background:**

Poliomyelitis is a highly infectious viral disease that affects children below the age of 15 years which is transmitted via the fecal-oral route from person to person. One in every 200 infections leads to irreversible paralysis, usually in the legs. Polio is preventable by immunization and earmarked by the World Health Organization for eradication. Sierra Leone is working toward certification of Poliomyelitis-free status. A suspected case of Polio is any child under 15 years of age with acute flaccid paralysis (AFP) or any person of any age with paralytic illness which the clinician suspects poliomyelitis involvement and a confirmed case is any suspected case from which the wild polio virus is isolated. The AFP surveillance system in Sierra Leone was evaluated to determine if the system was meeting its set objectives and to assess the system's attributes.

**Methods:**

We used guidelines developed by Centres for Disease Control and Prevention, Atlanta. We reviewed surveillance records and analyzed data sets on AFP from 2005 to 2012 from the National Surveillance Unit. We also interviewed stakeholders at all levels.

**Results:**

From 2005-2012, 8 wild polio virus case were identified in the country; in 2009 six wild polio cases were identified; Bo, Moyamba, Port-Loko and Western Area (Rural) had one case each whilst Kambia had 2. In 2010 and 2011; Western Area (Rural) identified a cases. The non-polio AFP rate increased from 2.4 to 6.2 cases per 100,000 children below 15 years from a target of 2 cases per 100,000 children below 15 years. The AFP surveillance system is integrated with other priority diseases and has a clear case definition and external laboratory support. The system is simple, flexible, sensitive, specific, stable and well represented. Incompleteness, poor data management and analysis at the District levels are the weaknesses. Vices are poor feedback, inadequate personnel and logistics.

**Conclusion:**

The AFP surveillance system meets set objectives but needs improvement in efficiency in logistics planning and handling, data management tools, data quality including completeness and analysis. Feedback from national levels through to the districts and below should be implemented and monitored including timely laboratory results.

# Sub article: Factors affecting disease notification and reporting among health workers in selected local government areas in Oyo State, Nigeria

**Published:** 2015-07-22

**Authors:** Dairo Magbagbeola David

**Affiliations:** 1 Nigeria Field Epidemiology and Laboratory and Training Program

## Abstract

**Background:**

Identification of gaps in relevant knowledge is required to design interventions to improve disease notification and reporting among health workers. This study assesses health workers knowledge, practices, and factors influencing disease reporting in urban and rural communities in Oyo State, Nigeria.

**Methods:**

A cross-sectional survey was carried out among 210 health workers who were focal persons responsible for disease reporting at their health facilities in one rural (Afijio LGA) and one urban (Ibadan North LGA) in Oyo State. A semi-structured, self-administered questionnaire was used to collect information on knowledge, practices and factors affecting reporting of immediate, routine, international and occupationally notifiable diseases. Knowledge was assessed on a scale of 50 points with score ≥30 as good. Data were analyzed using descriptive statistics; Chi square, t-test and linear regression.

**Results:**

Community Health Officers (30.1%), Nurses (26.0%) and Physicians (16.3%), constituted the majority of the respondents. Seventy-two percent (rural- 14.8% and urban- 57.1%) were aware of the existence of disease notification system while 26.5% knew the current strategy for reporting. Mean knowledge score for notifiable diseases among respondents was 27.6 ±8.4 with group means for rural and urban being 32.0 ±8.6 and 26.7 ±8.2 (p < 0.001) respectively. About eleven percent (11.2%) of the respondents had good knowledge of the notifiable diseases. Majority (82.8%) of the respondents forwarded their routine health facilities reports to their respective LGA while 17.1% sent theirs to the Ministry of Health. Fifty-six percent of respondents sent reports through their staff while the rest had their facilities report collected by staff from State Ministry of Health and LGA. Main reasons for non-reporting included: lack of training on reporting (84.0%), absence of legal enforcement (58.0%), ignorance of reporting requirements (50.0%) lack of supervision (48.0%) and lack of reporting forms and telephone facilities (38.0%). Health workers that were aware of notification system were five times likely to comply with reporting than those that were not aware. (OR = 5.0, 95% C.I = 1.5-17.5).

**Conclusion:**

Regular training, effective supervision and logistic support to responsible focal persons are recommended to prevent factors militating against reporting of notifiable diseases in the state.

# Sub article: High Rabies Mortality among children, a Secondary Data Analysis of the National Surveillance Rabies Data, Angola 2012

**Published:** 2015-07-22

**Authors:** Pedro Lussukamu

**Affiliations:** 1 Angola Field Epidemiology and Laboratory and Training Program

## Abstract

**Background:**

Lack of adaptation of the Rabies virus to their host, turns it to be one of the most feared diseases that affect human species. Unlike adults, children are most vulnerable to the disease due to their close association with infecting animals. Inadequate surveillance in most African countries makes it almost impossible to know the real magnitude of the problem. We aimed to describe the epidemiology of the disease and to evaluate the trends of the Diseases in Angola from 2006 to 2011.

**Methods:**

A descriptive analysis of secondary data from the National Surveillance Rabies data, for all provinces between 2006 and 2011 was done. A trend analysis to test on significant increase in Rabies cases over the years was performed.

**Results:**

Dog bites are reported for each province annually, with Luanda always having the largest annual number of bites followed by Benguela. A total of 963 deaths due to Rabies were reported between 2006 and 2011. There was a general increase in the fatality of cases between the periods. The annual distribution of cases was: 81(8.4%) in 2006; 118(12.3%) in 2007; 126(13.1%) in 2008; 249(25.8%) in 2009; 187(19.4%) in 2010 and 202(21.0%) in 2011, with the highest number of fatal cases recorded in 2008, indicating a significantly, (p = 0.028) increasing trend for cases over the years. However, there is a slightly predominance of cases during the months of January to February but the data does not indicate a clear seasonal tendency of the disease. The most affected age group with both bites and fatalities was the 4 - 9 year age group, 340(35%) followed by the 10 - 14 year age-group, 233(24.2%) and the 0 - 4 year age group 150(15.6%). Luanda is the most affected province with a total of 329(34.2%) fatal cases for the period followed by Huambo and Malange with 139(14.4%) and 94(9.8%) respectively.

**Conclusion:**

Children are most vulnerable to both bites and Rabies fatalities, with the 4 - 9 year age group mostly affected. There is need for massive awareness campaigns on Rabies and to educate people on the importance of animal vaccination especially in places with highest number of bites and case fatalities.

# Sub article: Sensitivity of Case-Based Measles Surveillance in Selected Local Government areas of Oyo State, Nigeria, January 2011- June 2012

**Published:** 2015-07-22

**Authors:** Adewuyi Peter

**Affiliations:** 1 Nigeria Field Epidemiology and Laboratory and Training Program

## Abstract

**Background:**

Measles is often under reported therefore appropriate public health interventions are not implemented. A sensitive system is essential to monitoring progress towards Measles elimination in Nigeria. However, the sensitivity of Measles surveillance in most states of the country is unknown. Therefore, we evaluated the Measles case based surveillance system in Oyo State, Nigeria.

**Methods:**

A cross sectional survey and a review of records was done. Cluster sampling technique was used. Oyo State was divided into 33 clusters based on the Local Government Areas (LGA). Six clusters were selected by simple random sampling. All the health facilities (75) in the selected clusters were involved in the study. Measles data collected included name, age, sex, address and date seen at health facility. Capture recapture method was used to estimate true number of Measles cases seen between January 2011 and June 2012. The Number of cases missed by the surveillance system was determined. Sensitivity was calculated by dividing the number of Measles cases recorded by the surveillance system, by the estimated true number of suspected Measles. Descriptive analysis was done and level of significance was at 95% C.I.

**Results:**

Of the 75 health facilities, the Measles surveillance system reported cases from 51(68%) health facilities. However, 56(75%) health facilities actually documented Measles cases in their registers. Twenty cases were reported/ notified in both the LGA and health facility registers. The estimated number of suspected Measles cases was 3,825 [95% C.I 2,363, 5,286]. The total missing cases in the LGAs were 3,197. The sensitivity of the surveillance system was 4.3%.

**Conclusion:**

The sensitivity of the Measles cases based surveillance system was very low, with very high estimated number of missing cases. There is need for public health intervention to improve upon the system.

# Sub article: Lead Residues in tissues of animals for human consumption at Gusau modern abattoir, Zamfara State, Nigeria

**Published:** 2015-07-22

**Authors:** Abubakar Jafiya

**Affiliations:** 1 Nigeria Field Epidemiology and Laboratory and Training Program

## Abstract

**Background:**

Lead is a bioaccumulative heavy metal usually found in nature combined with other elements to form lead compounds. Lead poisoning accounts for about 0.6% of global burden of diseases. Over 400 children were reported to have died as a result of lead poisoning outbreak in Zamfara State, Nigeria and about 2000 children are currently on treatment. We investigated the presence of lead residues in tissues of animals slaughtered at Gusau abattoir, Zamfara State, Nigeria.

**Methods:**

A total of 384 tissue samples of animals at slaughter were collected and tested for lead using Atomic Absorption Spectrophotometer. Information about species, source, age, sex, breed was collected using a structured questionnaire.

**Results:**

About 95% of the animals were sourced from markets within Zamfara State and out of all the samples from animals investigated 324 (84.4%) had lead residues. The lead residues in 314 (81.80%) of the animals were above the permissible limit of 0.1mg/kg recommended by World Health Organization and European Commission. Mean concentration of lead residues was highest (2.67mg/kg) in camels 10 years of age and (2.21mg/kg) in goats < 1 year. There was no significant difference (p > 0.05) observed in tissue accumulation of lead in animals based on difference in age, sex, species or breed of the animal.

**Conclusion:**

Lead residues are present in 84.4% of animals; therefore the environment from where these animals were sourced is contaminated with lead materials. Further investigation of animals at slaughter from other abattoirs within the State was recommended.

# Sub article: Prevalence and Risk factors for Mycobacterium Tuberculosis complex among slaughtered cattle and cattle handlers at Yaoundé South abattoir, Plateau state - Nigeria, 2012

**Published:** 2015-07-22

**Authors:** Lilian Okeke

**Affiliations:** 1 Nigeria Field Epidemiology and Laboratory and Training Program

## Abstract

**Background:**

Worldwide, Tuberculosis causes about 9 million new cases and 2 million deaths annually. Nigeria has the tenth highest burden of human Tuberculosis, Bovine Tuberculosis in humans is becoming increasingly important in developing countries like Nigeria because humans and animals share the same micro-environment and dwelling premises so the risk of zoonotic transmission is potentially high. This study determined the prevalence of Mycobacterium Tuberculosis (MTB) complex and factors associated with MTB complex among slaughtered cattle and cattle handlers at Yaoundé South abattoir in Plateau State.

**Methods:**

A cross sectional study design was employed. We collected 168 lung tissues with or without lesions from 485 slaughtered cattle consecutively and tested for acid fact bacilli (AFB) using Ziehl-Neelsen test and a duplex polymerase chain reaction technique for MTB complex detection. We collected sputum from 72 cattle handlers and obtained information on their socio-demographic characteristics, risk factors associated with MTB complex and information on their cattle. We performed descriptive, bivariate and multivariate analysis.

**Results:**

The mean age of the cattle was 5.6 years (SD ± 1.3), 64.3% were females. Prevalence of MTB complex was 21.4% by AFB test and 16.7% by duplex polymerase chain reaction. Out of 33 (19.6%) lung with lesion, 27 (75%) were positive for AFB while of 135 (75.0%) lungs without lesions, 9 (25%) were positive for AFB. Lung tissues with lesions were 52 times more likely to test positive to AFB test compared to those tissues without lesions (AOR = 52.3; 95% CI: 16.4-191.8). The mean age of the cattle handlers’ was 37 years (SD ± 32), 91.7% were males. Consumption of raw milk was associated with handlers’ education (p < 0.05) and occupation (p < 0.05). All sputum samples tested for acid fast bacilli were negative.

**Conclusion:**

The study determined the prevalence of Mycobacterium Tuberculosis complex of 21.4% by acid fast bacilli test and 16.3% by a duplex polymerase chain reaction. AFB positivity increased with the presence of lesion on tissue from slaughtered cattle. Consumption of raw milk was associated with cattle handlers’ education and occupation. Health education of handler’ is urgently required. Active surveillance for MTB complex at abattoirs is recommended.

# Sub article: Genotypes of Rotaviruses among Porcine and Children under five Years in the Ga East Municipality of the Greater Accra Region- Ghana

**Published:** 2015-07-22

**Authors:** Helena Acquah

**Affiliations:** 1 Ghana Field Epidemiology and Laboratory and Training Program

## Abstract

**Background:**

Rotavirus Diarrhea is a public health problem worldwide. Human rotavirus claims approximately 527,000 lives each year. Porcine rotaviruses have been estimated to be responsible for 10-15% of all Diarrhea cases and for large economic losses in pig production. Rotavirus is a genus of the family Reoviridae. Group A rotavirus is highly diverse antigenically and genetically. Animal rotaviruses have been shown to infect human. There is scarcity of data in Ghana to support domestic animals as the reservoir of rotaviruses. This study aimed to determine the prevalence and genotype distribution of rotavirus in pig farms and in children less than five years in the Ga East Municipal from January to March 2012, to assess evidence of interspecies transmission between human and porcine rotaviruses strains detected, to determine the risk factors of rotavirus infection and the rate of asymptomatic shedding of rotavirus among pigs in the study area.

**Methods:**

This study was a cross sectional, using qualitative and quantitative methods of data collection in the Ga East Municipality. The study involved children > 5 and pigs less than twenty weeks old. Stool samples were obtained from children less than five years with Diarrhea from the municipality. Data and 107 faecal samples of pigs were obtained from 28 selected farms. Rotaviruses were detected by PAGE and genotypes determined by reverse transcription polymerase chain reaction (RT-PCR). Univariate and multivariate analysis were conducted with the significance level set at 0.05.

**Results:**

Genotypes detected in children were G1P [8] and G12P [8] and that for porcine were G6, G10, P [5] and P [6]. No common genotype between children and porcine was detected. Incidence of rotavirus in children and pigs was 38.5% and 16.8% respectively. Incidence on pig farms was 46.5% and of asymptomatic shedding of porcine rotaviruses 77.8%, in weaners was 38.9%.

**Conclusion:**

The circulating rotavirus genotypes amongst children in the Ga East Municipality were, G1P [8] and G12P [8] and that of pigs were G10, G6, P[5], P[6]. Incidence of asymptomatic shedding of porcine rotaviruses is high. Keeping cattle, pets and using pig waste as fertilizer on farms were significant risk factors with rotavirus infection.

# Sub article: Evaluation of Measles Surveillance System in Ghana, 2012

**Published:** 2015-07-22

**Authors:** Godfrey Konnyebal

**Affiliations:** 1 Ghana Field Epidemiology and Laboratory and Training Program

## Abstract

**Background:**

Measles is a major cause of death among young children globally even though it is vaccine preventable. Up to 10% of Measles cases result in death. An estimated 158,000 children - mostly under five years died from Measles in 2011. More than 95% of these deaths occurred in Asia and Africa. Between 2005 and 2011 Ghana recorded a total of 464 confirmed Measles cases with no death. An effective surveillance system is needed to detect and manage cases early to prevent avoidable deaths, monitor trends and guide Supplementary Immunization Activities (SIA). We evaluated the system to assess its attributes and determine whether it is meeting its objectives or not.

**Methods:**

We described the Measles surveillance system and engaged stakeholders at all levels. An interview guide and a checklist were used to obtain data from persons directly involved in Measles -surveillance in Ghana. Data sets, facility records and case based forms from 2008-2011 were reviewed at various levels and analyzed.

**Results:**

We found that the system is integrated into the integrated disease surveillance and response (IDSR) system with clear channel of communication. It is able to detect cases and investigate all outbreaks. Positive predictive value (PVP) is 6.9%. Reporting is timely but feedback is poor. Data quality is poor and analysis is not thorough. The system is able to monitor Measles trends and guide Supplementary immunization Activities (SIA).

**Conclusion:**

The system is sensitive, flexible, stable but complex. It is useful and meeting its objectives with few challenges. We recommended that the information flow be modified so that the Public Health Reference Laboratory can give feedback promptly and directly to lower levels: Disease control officers and health information officers at all levels be given in-service training on surveillance data collection and analysis.

# Sub article: Polio Outbreak investigation in Dadaab Refugee Complex, Kenya, September 2012

**Published:** 2015-07-22

**Authors:** Said Shariff

**Affiliations:** 1 Kenya Field Epidemiology and Laboratory and Training Program

## Abstract

**Background:**

In July 2012, polio outbreak was notified to the Ministry of Public Health and Sanitation of Kenya. Two cases of acute flaccid paralysis (AFP) were detected in IFO-2 camp in Dadaab Refugee Complex, a case and one contact of AFP were found to have cVDPV genetically related to cVDPV cases from Somalia of 2011. Rarely, OPV can become virulent and cause AFP in the vaccine recipient and be transmitted within unimmunized populations referred to as circulating Vaccine-Derived Polioviruses (cVDPVs). We conducted an investigation to determine the magnitude of the outbreak, characterize the cases and to provide recommendations for further action.

**Methods:**

We carried out a descriptive study to investigate cases and contacts of AFP. A cVDPV case was defined as any child <15 years of age with AFP or any person of any age with acute onset of paralytic illness residing in Dadaab since 1st July 2012. Stool specimen was collected from cases and contacts for isolation of polioviruses. A standardized line-listing tool was used to extract epidemiological information from medical records from 1st April, 2012 to 31st August, 2012.

**Results:**

We enrolled 26 respondents; 2 cases and 24 contacts. The mean age of the affected children was 3 years (range 1-5, IQR; 3years). Seventeen (65.4%) of the respondents were males. Five (19.2%) of the contacts were laboratory confirmed for cVDPV. Sixty-one percent lived in household with more than seven occupants and only 42% had latrines. Twenty (76.9%) were not vaccinated against polio. While 23% of the respondents had received routine OPV at least once, others received OPV only during National Supplementary Immunizaton Days (NSID). Fourteen (54%) of the respondents had no basic education, 11 (42%) had religious education while only 1 (4%) had primary education.

**Conclusion:**

The disease notably affected the lower age group. Routine OPV immunization is low in Somali refugee children in Dadaab. Overcrowding and lack of latrines probably facilitated transmission of cVDPVs in Dadaab. Increasing routine immunization program outreach and conducting supplemental polio immunization campaigns are necessary to stop transmission of cVDPVs in Dadaab.

# Sub article: Evaluation of the Acute Flaccid Paralysis Surveillance System in the Biyem-Assi Health District from 2009 to 2011 - Yaoundé, Cameroon, 2012

**Published:** 2015-07-22

**Authors:** Aba Akam

**Affiliations:** 1 Central African Field Epidemiology and Laboratory Training Program

## Abstract

**Background:**

Acute Flaccid Paralysis (AFP) surveillance system has as objective to globally eradicate the wild polio virus (WPV). In the 2013-2018 Endgame Strategic Plan, stakeholders intend to archive a “polio-free world” by 2018. In 2010, this District accounted for eight of 15 (53.3%) AFP cases investigated in Yaoundé, recording an annual non-polio AFP rate (ANPR) of 12/100,000; the national minimum rate being 3/100,000. To appreciate the quality and reliability of this surveillance system, we evaluated its different attributes.

**Methods:**

The Updated Guidelines for Evaluating Surveillance System (CDC, 2001) were used to conduct an evaluation of the attributes of the AFP surveillance system: Simplicity, Flexibility, Data Quality, Acceptability, Sensitivity, Positive Predictive Value (PPV), Representativeness, and Timeliness. Sites selection was purposeful: all 26 surveillance sites of high and moderate surveillance priorities were involved. Fifty-seven staff implicated in the surveillance system were interviewed using structured questionnaires. Complementary information was gotten through document review. Performance standard(s) for each attribute was/were set in line with national surveillance objectives. Data was analyzed using Epi-Info3.5.3 and Microsoft Excel.

**Results:**

Only 26.9% of the different key components of the AFP case definition were mastered by participants, making system not simple. Although this case definition was flexibly modified and put in use, it was displayed in just 30 of 57 (52.6%) consultation posts. Forty-eight of 657 (7.3%) spaces in the surveillance forms were blank or invalidly-filled, thereby compromising the data quality. Twenty-two of 57 (38.6%) respondents mastered criteria for stool adequacy. The system's sensitivity rated “good”, having registered an aggregate ANPR of 7/100,000 and no missed cases found in consultation registers. Laboratory results for investigated cases were negative, resulting in a 0.0% PPV. Representativeness was unsatisfactory, as only 40 of 66 (60.6%) existing health facilities are implicated in the surveillance system. Fifty-eight out of 71 time-related surveillance indicators were realized within the recommended timeframes, giving an aggregate timeliness of 81.7%. The surveillance system was stable, having functioned uninterrupted within this period.

**Conclusion:**

The AFP surveillance system was sensitive, flexible and stable. However, the system fell short of the standards of simplicity, data quality, acceptability, representativeness, and timeliness.

# Sub article: Investigation of Measles Outbreak, Meta Robi District, Central Ethiopia, February2013

**Published:** 2015-07-22

**Authors:** Meaza Tilaye

**Affiliations:** 1 Ethiopia Field Epidemiology and Training Program

## Abstract

**Background:**

Measles is a highly contagious, serious disease caused by a virus in the family Paramyxoviridae. Based on the rumors obtained from the District subsequent investigation for verifying the existence of Measles outbreak and identification of the risk factors was made to institute interventional measures.

**Methods:**

Unmatched case control supported by descriptive cross sectional study was undertaken. Structured questionnaire was used to collect data from cases and controls coupled with laboratory investigation. Cases were those febrile individuals who presented with generalized maculo-papular rash and one or more symptoms of cough, coryza and conjunctivitis. Controls were neighbors of cases but without signs and symptoms. Bivariate statistical analysis was conducted using Epi-Info version3.5.1 to find association of illness with risk factors.

**Results:**

A total of 125 suspected cases of Measles were identified with an overall attack rate of 7.6 per 10, 000, and case fatality rate was 8 per 1000. The attack rate with in different age groups were 1,0.8 and 0.3 per 1000 for 5-14 year,0-4 year and 15 years and above age groups respectively. Majority of cases 81(64.8%) had within 5-14 years age group. The median age of cases was 9 (range of 1 to 26 years). The median age of controls was 9 (range of 1 to 45years). Of the total cases and controls 111 (88.8%) of the cases and 73(58.9%) of the controls had contact history with cases at school, at home or with a neighbor. Exposure to a Measles cases was significantly associated with illness (OR = 5.54, 95% CI 2.86-10.73). There was 62% reduction of Measles infection among those who received Measles vaccination with OR 0.38 and 95% CI 0.22-0.63.

**Conclusion:**

We confirmed Measles outbreak. Most of the cases were school age children who had contact history with other Measles case and are not vaccinated. We conducted social mobilization activities including schools awareness creation on the disease and health facilities were alerted about the outbreak to limit the disease. Measles vaccines were administered to all < 5 years old as part of prevention.

# Sub article: Evaluation of the Surveillance System of Flaccid Acute Paralysis /Poliomyelitis in Luanda- Angola, 2012

**Published:** 2015-07-22

**Authors:** Maria Yaoundé Soares

**Affiliations:** 1 Angola Field Epidemiology and Laboratory and Training Program

## Abstract

**Background:**

The World Health Organization (WHO) signed a commitment to eradicate poliomyelitis globally in 1988. Angola has not recorded a poliomyelitis case since July 2011. To be certified free from poliomyelitis, a country has to report no case of the disease for three consecutive years and to have a high quality epidemiologic surveillance. The aim of this study was to evaluate the surveillance system by assessing the attributes simplicity, sensitivity, and timeliness and to identify weakness on the system that may need to be reinforced.

**Methods:**

A cross-sectional study was conducted at all reference health units in Luanda where39 participants working in the health units were interviewed. Timeliness was evaluated using WHO recommended rates for timely and adequate stool sample collection. A table review was performed using a check list to evaluate the representativeness. We evaluated sensitivity using surveillance time period of one year, on the ability of the system to detect cases at a prevalence of 2 cases per 100 000 persons. The attribute simplicity was evaluated at two levels; from key informants and surveillance officers.

**Results:**

All key informants perceived the system difficult as it had five case definitions. However, the respondents found the forms easy to fill, 24 (75%) took 10 minutes; only 3 (9.5%) 20minutes; 3/9 municipalities did not met the target of detecting 2 cases per 100000 persons. From the 53 of the reviewed investigation forms for 2011, 34 (64.2%) were from age group 0-5 years, of them 30(56%) were male and 30(56%) female. Cases were notified with more prevalence in the months of February and March 18(33.9%), 8/9 municipalities met the WHO target of 80% for adequate stool collection rate.

**Conclusion:**

The existence of the five case definitions made the system not to be simple. Some of the municipalities were not able to meet WHO target on the timely sample collection. The system was timely and representative but not simple and not sensitive enough. We recommended regular refresher training for the surveillance officers to optimize AFP case detection. Ongoing supervision activities for each municipality have been implemented and regularly vaccination campaigns are being done.

# Sub article: Knowledge and Beliefs on Cervical Cancer and practices on Cervical Cancer screening among women, Ouagadougou, Burkina Faso, 2012

**Published:** 2015-07-22

**Authors:** Sawadogo Bernard

**Affiliations:** 1 West Africa Field Epidemiology and Laboratory and Training Program

## Abstract

**Background:**

In Burkina Faso, 3.8 million women ages 15 years and older are at risk of developing cervical cancer, 1230 are diagnosed with Cervical Cancer every year and 838 die from the disease. Screening rate is low and little is known about women's practices, knowledge and beliefs regarding cervical cancer. This study aims to describe women's practices regarding Cervical Cancer screening and to assess their knowledge and beliefs in order to provide health education area to enhance Cervical Cancer screening.

**Methods:**

A cross-sectional study was carried out in Ouagadougou interviewing 830 women living in Ouagadougou, aged 20 to 50 years about their knowledge, beliefs and practices regarding cervical cancer. Cluster sampling was used and women were asked about their history of Cervical Cancer screening, socio-demographic factors, risk behavior factors, knowledge, attitude and beliefs on cervical cancer. Epi-Info version 3.5.1 was used for data entry; univariate and multivariate logistic regression analysis were performed. Chi square test was used and p-value 0.05 was considered.

**Results:**

830 women were enrolled. Mean age was 29.5 ±7.77 years. 554/830 (66.74%) of participant were married and 493/830 (59.39%) have not been to school or left school at primary level. 378/830 (45.54%) of participant were using oral contraceptive and 649/830 (78.19%) had their first intersexual course at 20 years age or below. Of the 830 participant 378 (45.54%) had history of STI. While 534/830 (64.33%) of participant heart about cervical cancer, only 68/830 (8.19%) heart about Human papillomavirus. 649/830 (77%) don't know how Human papillomavirus is transmitted and 557/830 (67.10%) don't know that Cervical Cancer is preventable disease. Most participant 755/830 (90.96%) were worried to develop Cervical Cancer and 806/830 (97.10%) would accept to be screened. Of 830 participant only 93 (11.20%) were screened for cervical cancer. Worry to develop cervical cancer, heart about Human papillomavirus and about Cervical Cancer are significantly associated with screening history with p < 0.05.

**Conclusion:**

Knowledge and belief regarding Cervical Cancer is limited among Ouagadougou women and screening rate is low. There is need to enhance health education regarding Human papillomavirus and cervical cancer.

# Sub article: The Epidemiology of Rubella disease in Ethiopia: 2008-2012

**Published:** 2015-07-22

**Authors:** Etsehiwot Zemelak

**Affiliations:** 1 Ethiopia Field Epidemiology and Training Program

## Abstract

**Background:**

Rubella is a mild viral disease. However Rubella infection in pregnancy is major public health importance due to the teratogenicity effects that can result from congenital Rubella syndrome (CRS). In Ethiopia, Rubella surveillance system is not established and therefore information about the epidemiological burden of Rubella disease is unknown. This study was conducted to describe the epidemiology of Rubella disease in Ethiopia.

**Methods:**

Five year (2008-2012) data of Rubella disease which was reported through Measles case based surveillance system for laboratory confirmation of Measles and Rubella IgM antibody to the Ethiopian Health and Nutrition Research Institute (EHNRI) was analyzed to describe the burden and epidemiology of Rubella disease in Ethiopia.

**Results:**

A total of 11,026 samples were tested for Rubella IgM and 1346(12%) cases were positive for Rubella. The vast majority, 1231(91%) of cases were below the age of 15 years and 53% were females. Among the total Rubella positive cases, 1317(98%) had information on patient area of residence (rural vs. urban); of these, 684 (51%) were from rural area and 633(47%) from urban area. Addis Ababa 275 (2/100,000), Hareri 18 (1.8/100,000) and Dire Dawa 24 (1.3/100,000) contributed higher number of cases. Within the five year period, the annual incidence of Rubella cases increased 167% from 0.3/100,000 in 2008 to 0.9/100000 in 2012. Annually, increments in the number of cases were observed from March to June.

**Conclusion:**

Rubella is common in young children's below the age of 15 years. The disease is widely distributed throughout the country and it is more common in rural areas than urban areas. It occurred seasonally with peak from March to June. To further understand the burden and epidemiology of Rubella and CRS in Ethiopia surveillance system should be established.

# Sub article: Yellow Fever Outbreak in Kundungu Sub-District of Upper West Region, Ghana, March 2011

**Published:** 2015-07-22

**Authors:** Dodzi Kofi Amelor

**Affiliations:** 1 Ghana Field Epidemiology and Laboratory and Training Program

## Abstract

**Background:**

Yellow fever is an acute, febrile, mosquito-borne viral disease characterized in severe cases by jaundice, albuminuria, and hemorrhage. It is endemic in tropical and subtropical regions of Africa and South America. A suspected outbreak of Yellow fever was reported in Kundungu in the Wa East District, Upper West region-Ghana. We investigated to characterize the source and magnitude of the outbreak.

**Methods:**

We interviewed District Health Management Team members and hospital staff at Kundungu using an interview guide. We extracted and abstracted data from records at the District and regional levels. A suspected Yellow fever case-patient was any person with fever and jaundice or epidemiologically- linked to a person with these signs in kundungu and contiguous communities between February-March, 2011. Blood samples were collected and entomological assessment was done.

**Results:**

Of the four suspected cases, 3 (75%) were males within same household. The index-case, a 16-year old male had positive ELISA test for Yellow fever, confirmed by PCR at the WHO Dakar reference laboratory. Two of the three persons epidemiologically linked to the confirmed-case tested negative giving a population attack rate of 8/10000 population. There was no fatality and no cases were found during the active case-search in Kundungu and adjoining communities. Entomological study identified larvae of Aedes mosquito with a breteaux index of 6.

**Conclusion:**

A focal Sylvatic Yellow fever outbreak with no fatality and limited to an endemic sub-District occurred in the Upper-west region of Ghana. Exposure to Aedes mosquitoes with a breteaux index within the WHO threshold of 5-50 considered sufficient to promote an outbreak is implicated. We recommended for more health education in affected and adjoining communities. Refresher-seminars on Yellow fever were conducted for health workers at the community, District and regional levels. A Yellow fever vaccination was carried out in November, 2011.

# Sub article: Burden of animal bites in human populations, Risk factors and Rabies control efforts, Mubende District, 2012

**Published:** 2015-07-22

**Authors:** Grace Asiimwe

**Affiliations:** 1 Uganda Field Epidemiology and Training Program

## Abstract

**Background:**

Rabies is a viral zoonosis with global distribution, though under reported in developing countries. Humans acquire Rabies through bites by infected animals. Uganda reported 12,718 cases and 203 deaths from animal bites in 2010 with 82 cases from Mubende District. There was an increase in human animal bite cases in the District in 2011 and we initiated an investigation to describe trends of animal bites in human populations, risk factors and Rabies control efforts in the District, to guide public health policies aimed at enhancing human and animal health and prevent Rabies.

**Methods:**

We reviewed and abstracted reported animal bite cases from Mubende hospital and Veterinary records from 2010 to 2011. Cross sectional study conducted with animal bite cases and non-cases identified actively from the community. Self-administered interviews with both case and non-case households, Health and Veterinary personnel were done using a structured questionnaire. Data was entered and analyzed using Epi-Info 3.5.1.

**Results:**

In 2010 and 2011, 563 patients reported animal bites from all 15 sub counties to Veterinary department and referral hospital. Majority of the bites were due to dogs 540 (96%) most of which were stray, cats12 (2.1%), Jackals 5 (0.9%), goats3 (0.5%) and 0.5% (3/563) of the reported bites were inflicted by fellow humans. Patients were aged between 3 months and 84 years (median 13 and mean 20 years.) with females 293/563 (52%) most affected. Only 541 (96.1%) of the patients were treated with first dose of post exposure Rabies vaccine and 125/563 (23.1%) received 2nd and 3rd vaccine doses. Age group 10-19 years (28.3%) and school children walking to school (69.2%) showed the highest incidence of animal bites.

**Conclusion:**

Animal bites and human Rabies in Mubende District are primarily due to stray dogs. Rabies control measures are inadequate to prevent further animal bites and Rabies. Post exposure prophylaxis is inadequate to reduce mortalities. Further sensitization of the public especially school children on prevention, treatment and control strategies with mass vaccination of pets and elimination of stray dogs with combined efforts from both veterinary and medical staff is desired.

# Sub article: Knowledge, attitudes and practices related to Rabies among animal and human Health workers in Mbale, Uganda- 2013

**Published:** 2015-07-22

**Authors:** Fred Monje

**Affiliations:** 1 Uganda Field Epidemiology and Training Program

## Abstract

**Background:**

Rabies is a viral, zoonosis, killing over 55000 people globally and yet endemic in Uganda. Unfortunately, for the last five years, Mbale has persistently registered the highest number of suspected Rabies cases in Eastern Uganda, presented as 587 animal bites on average in humans and at least 19 human deaths annually. Sadly, knowledge, attitudes and practices (KAP) of health workers regarding animal bites or Rabies is uncertain. We set out to determine KAP regarding Rabies among animal and human health workers so as to inform policy makers and provide a basis for urgent action.

**Methods:**

A cross-sectional study was conducted between December 2012 and March 2013 among 147 animal and human health workers working in Mbale and consented to the study. Only 131 human health workers (nurses, clinical officers and medical officers) and 16 animal health workers were randomly selected after proportion to size sampling and interviewed using a semi-structured questionnaire. Data was entered into Epi-Info and exported to STATA for analysis.

**Results:**

Of all the respondents, only 65 (44.22%) had sufficient knowledge about Rabies. Three quarters of animal health workers, 12(75%), had sufficient knowledge about Rabies compared to only 53(40.46%) human health workers. Knowledge of Rabies was influenced by availability of refresher trainings (OR: 4.57; 95%CI: 1.79-11.62) and the type of qualification (OR: 3.45; 95%CI: 1.82-6.53). About a quarter of the respondents, 37(25.17%) had positive attitudes. Among the animal health workers, 7(43.75%) had positive attitudes towards Rabies management compared to30 (22.90%) human health workers. Refresher trainings (OR: 7.86; 95%CI: 2.71-22.82) influenced attitudes towards Rabies management. Nearly a half of the respondents, 73(49.66%), had poor practices. Respondents, knowledgeable about Rabies (OR = 3.65; 95%CI: 1.60-8.3) were more likely to have positive attitude towards Rabies management. Respondents with positive attitudes (OR: 2.22; 95%CI: 1.01-- 4.86), were more likely to have good practices against Rabies.

**Conclusion:**

Respondents had low knowledge, negative attitudes and poor practices against Rabies management. District heads should spearhead regular refresher trainings about Rabies to broaden staff knowledge and improve their attitudes and hence practices.

# Sub article: Trend and Risk factors of Poliomyelitis Transmission in Zamfara State, Nigeria -2009-2011

**Published:** 2015-07-22

**Authors:** Kabiru Getso

**Affiliations:** 1 Nigeria Field Epidemiology and Laboratory and Training Program

## Abstract

**Background:**

Poliomyelitis is a disease of public health importance which mainly affects children under 5 years of age. Nigeria is one of the three polio-endemic countries in the world and has the potential to serve as foci for transmission of Poliomyelitis to countries/regions that are certified polio-free. Data on Wild Polio Virus (WPV) cases from Zamfara State were analyzed to determine burden and trend of poliomyelitis in the State.

**Methods:**

We conducted descriptive analysis of data of WPV cases in August, 2011. Data from 2009 to 2011, obtained from Ministry of Health and WHO were reviewed. We performed univariate and bivariate analyses using Epi-Info 3.5.3 to obtain frequencies, proportions and odds ratios.

**Results:**

A total of 23 cases were reported during the period under review. Fifteen WPV cases were recorded in 2009, 5 cases in 2010 and 3 cases in 2011. Out of 23 cases recorded from 2009 to 2011, 14 (60.9%) were males and more than half (52.2%) were children aged 13 to 24 months. Additionally, 17.4% and 82.6% were cases of WPV1 and WPV3 respectively. Only 17.4% of the cases had ≥ 4 doses of Oral Polio Vaccine (OPV). Altogether, 18 (78.3%) of the cases came from rural settlements. Age of 13-24 months (OR: 2.99, 95% CI: 1.02-8.79), being from rural settlement (OR: 2.94, 95% CI: 1.07-8.06) and having received OPV < 3 doses (OR: 7.43, 95% CI: 1.73-31.93) were found to be significant factors associated with Poliomyelitis in Zamfara State.

**Conclusion:**

The trend of poliomyelitis in Zamfara State was declining and the burden was highest among male children aged 13 to 24 months which was largely due to WPV3. Age 13-24 months, living in rural settlement and having received OPV < 3 doses were identified significant factors for Poliomyelitis in the State. Government should strengthen AFP surveillance and scale- up routine and supplemental immunizations with OPV especially in the rural areas.

# Sub article: Acceptability of new combined campaign for Polio and Meningitis: Kumbotso LGA, Kano State, Nigeria- September 2012

**Published:** 2015-07-22

**Authors:** Aishatu Sadiq

**Affiliations:** 1 Nigeria Field Epidemiology and Laboratory and Training Program

## Abstract

**Background:**

Nigeria is prone to epidemic meningitis and maintains uninterrupted wild polio virus (WPV) transmission. Polio immunization campaigns currently target under-five year old children yet older age-groups could play a role in transmitting poliovirus. The introduction of a new conjugated meningococcal vaccine protecting children for ten years is planned in northern Nigeria. We assessed social acceptability of the new meningitis vaccine and expansion of the target age-group for polio vaccination in combined campaigns.

**Methods:**

We conducted key informant interviews with local government immunization officers (LIOs) and community mobilizers regarding their community's perceptions towards polio and the new meningitis vaccine in Kano State. One hundred and thirty five caregivers and stakeholders were recruited into 12 Focus Group Discussions (FGDs). Data was analyzed by thematic fields using a coding sheet.

**Results:**

Meningitis and polio were considered significant health problems by 11/12 groups (92%), eight (67%) mentioned vaccination as a means of preventing meningitis. Ten (83%) identified OPV as a means of polio prevention. Polio transmission risk considered present in older age-groups by four groups (33%). Affirmative responses regarding the new meningitis vaccine and the vaccination of older age-groups against polio: “Yes, these diseases kill all those affected in a very short time, and in large numbers” and “it's safe if there is sufficient information”. Involvement of traditional community leaders was considered by six groups (50%) essential to community acceptance of the new vaccine.

**Conclusion:**

Active community mobilization including traditional and religious leaders and mass media campaigns in local languages are essential to success of the new initiative. Adequate dissemination of information will ensure acceptance of the new vaccine. Planned seroprevalence studies involving older age -groups might address communities request for more information.

# Sub article: Acute Flaccid Paralysis (AFP) in Ethiopia, 2008-2012

**Published:** 2015-07-22

**Authors:** Woubayehu Kassa

**Affiliations:** 1 Ethiopia Field Epidemiology and Training Program

## Abstract

**Background:**

The global eradication of poliomyelitis (polio) depends on a combination of high immunization coverage and syndrome surveillance for acute flaccid paralysis (AFP) among children less than fifteen years of age. Ethiopia has been free of wild poliovirus since 2008 when it was imported into the Gambela region. Prior to that, it was imported from Somalia in 2006. The last case of indigenous wild poliovirus in Ethiopia occurred in 2001. We analyzed AFP surveillance data in Ethiopia from 2008-2012 to assess the immunization status of those reported cases, fulfillment of indicator criteria and the distribution and burden of wild and vaccine derived polioviruses.

**Methods:**

We conducted a descriptive cross sectional study on the national acute flaccid paralysis data from 2008 - 2012 in Ethiopia. Data were collected from Public Health Emergency Management (PHEM) and triangulated with data from the World Health Organization (WHO). Data cleaning and analysis was conducted by using EpiInfo version 7 and Microsoft Excel.

**Results:**

A total of 5042 AFP cases were investigated and tested from 2008-2012. Out of these 03(60/100,000) cases were confirmed wild polio virus which were imported cases reported from Gambela Region in 2008. Two of the cases had taken 3 polio doses and one case had taken <3 polio doses of vaccine. There were 12(240/100,000) cases of vaccine derived polio virus of which five cases had taken ≥3 doses of vaccine and seven cases took <3 doses of vaccine. There were 4733 discarded or negative cases and 65 cases were compatible with poliomyelitis after 60 days follow up. The proportion of non-polio acute flaccid paralysis (NP-AFP) cases with two stool specimens collected within 14 days of onset of paralysis was above 2/100.000 for all years and the stool adequacy rate was ≥80% which fulfilled 2 important indicators.

**Conclusion:**

Ethiopia has been free of indigenous polio virus since 2001 and free of imported polio virus since 2008. To eradicate polio virus important indicators should be met for every case. Case notification within 14 days and stool adequacy rates >80% are two indicators being met in Ethiopia. Continued AFP surveillance is a public health priority in Ethiopia in the global goal towards polio eradication.

# Sub article: Routine immunization coverage and associated factors among children aged 12-23months in Odeda Local Government Area, Ogun State, Nigeria

**Published:** 2015-07-22

**Authors:** Dairo Magbagbeola David

**Affiliations:** 1 Nigeria Field Epidemiology and Laboratory and Training Program

## Abstract

**Background:**

Available evidence suggests that these diseases contribute significantly to childhood morbidity and mortality in spite of concerted efforts aimed at preventing them through Routine Immunization (RI). The status of immunization coverage and the factors which militate against full RI are yet to be fully explored. The study was therefore conducted to assess the status of routine immunization coverage and factors responsible for incomplete coverage in Odeda Local Government Area, Ogun State Nigeria.

**Methods:**

Survey was carried out among 510 women with children aged 12-23 months. Respondents were selected using the WHO cluster sampling technique. A pre-tested structured questionnaire was used to obtain information on mothers’ knowledge about immunization and immunization status of their children aged 12 – 23 months. A child was considered fully immunized if he has received a dose of Bacillus Calmette - Guerin (BCG), three doses of Oral Polio Vaccine (OPV), three doses of Diphtheria – Pertussis – Tetanus (DPT), three doses of Hepatitis B Vaccine (HBV), and a dose each of Measles and Yellow Fever vaccines by 12 months of age while a partially immunized child would have missed one or more of these doses. A composite knowledge score about immunization was computed for each respondent on a 7 - item scale. Respondents with scores of ≥5 and <5 points were considered to have adequate and inadequate knowledge respectively. Descriptive, Chi-square and logistic regression statistics were used for data analysis at p = 0.05.

**Results:**

Mean age of the respondents was 28.8 ± 5.4 years, 68% had formal education and 57.3% were employed. The mean age of children was 17.9 to 3.9 months and 49.2% of them were males. Full immunization coverage was 22.2% while 51.9% were partially immunized and 25.9% were not immunized at all. Full coverage in respect of each of the antigens were BCG (57.5%), OPV (21.7%), DPT (23.8%), HBV (14.6%), measles (59.5%) and yellow fever (60.5%). Majority (68.3%) had adequate knowledge about immunization. Respondents with adequate knowledge were more likely to have their children fully immunized than those with inadequate knowledge (OR = 1.65, 95% CI = 1.024- 2.67). Thirty percent of respondents with secondary education had their children fully immunized compared to 17.3% of the non-educated ones. Employed mothers were twice more likely to fully immunize their children than unemployed mothers (OR = 2.3, 95% CI= 1.4-3.8). Inconvenient immunization scheduling (14.4%), lack of social support (12.2%), lack of knowledge about need for additional doses (9.4%) and lack of vaccine (8.1%) were among the reasons for incomplete routine immunization coverage.

**Conclusion:**

Full immunization coverage among children aged 12-23 months old in the study area was below the national target of at least 80.0%. Inadequate knowledge of mothers and logistic problems are the major limitations to inadequate coverage. Public enlightenment, social support and ready availability of vaccines are needed to address these challenges.

# Sub article: Upsurge of Animal Rabies in Greater- Accra, Ghana, 2007-2011

**Published:** 2015-07-22

**Authors:** Perdita Lopes

**Affiliations:** 1 Ghana Field Epidemiology and Laboratory and Training Program

## Abstract

**Background:**

Rabies, a highly fatal, viral disease caused by a Lyssa virus from the family Rhadoviridae, is acquired through the bite of an infected animal, mostly dogs. The disease and can affect all warm- blooded vertebrates including man. Estimated annual global human mortalities from Rabies is 55,000, of which 31,000 and 24,000 are from Asia and Africa respectively where an estimated US$ 583.5 million is spent on its control. Rabies has become endemic in Ghana. We undertook an analysis of Rabies data in the Greater-Accra Region to determine the trend of human and animal Rabies, and assess percentage coverage of animal anti-Rabies vaccinations annually from 2007-2011.

**Methods:**

We reviewed vaccination data for all ten Districts in the region, interviewed staff of the regional Rabies desk, and abstracted data from post-mortem and quarantine records of the National Veterinary Laboratory. Further, we reviewed human Rabies records from the Fevers Unit of the Korle-Bu Teaching Hospital and the Regional Public Health Directorate. Data was entered and analyzed in Microsoft Excel. We calculated percentages, drew graphs and analyzed trends.

**Results:**

Of a total of 309 animal specimens tested during the period, 283/309 were positive for Rabies; predictive value positive being 91.6%. Of the positive cases, 272 (283) (96.1%) were dogs, and 276/283 from animals with no previous vaccination history. The odds of a vaccinated animal testing positive for Rabies were about the same as that for animals with no previous vaccination history (Χ^2^ =0.42, {Fishers exact test= 0.54}). Of the specimens submitted for testing, 174/319 (54.5%) were from animals which had bitten 324 humans, and 55/174 bit more than one person, whiles 64/324 (19.8%) victims were bitten by their own dogs. The mean number of human rabid deaths from 2008-2011 was five. Pet vaccinations in the region were lowest in 2010, at 439. Interestingly, the number of outbreaks was highest in the same year, at 34.

**Conclusion:**

The upsurge in Rabies outbreaks could be due to the absence of simultaneous mass vaccinations across districts. Government should re-introduce free annual mass vaccination of pets. Key stakeholders should forge closer collaborations in Rabies control.

# Sub article: Sexually Transmitted Infections (STI) and infertility in Rwanda: Diagnostic significance of Molecular and IgG and IgA Antibodies testing

**Published:** 2015-07-22

**Authors:** Claude Muvunyi

**Affiliations:** 1 Rwanda Field Epidemiology and Laboratory and Training Program

## Abstract

**Background:**

In many developing countries, little is known about the prevalence of genital Chlamydia trachomatis infections and complications, such as infertility, thus preventing any policy from being formulated regarding screening for C. trachomatis of patients at risk for infertility. The objective of the present study was to determine the prevalence of C. trachomatis and evaluate the diagnostic utility of serological markers namely anti- C trachomatis IgG and IgA antibodies in women attending an infertility clinic.

**Methods:**

Two commercial species-specific ELISA to determine serum IgG and IgA antibodies to C. trachomatis, PCR on vaginal swabs specimens and Hysterosalpingography (HSG) was performed on Serum and vaginal swab specimens of 303 women presenting with infertility to the infertility clinic of the Kigali University Teaching Hospital and 312 fertile controls in sub-fertile women.

**Results:**

The prevalence of C. trachomatis infection by PCR and serological test (IgG and IgA) were relatively low in both sub-fertile and fertile women and no significant differences in overall prevalence rates of C. trachomatis among both groups were observed. The only factor associated with C. trachomatis infection in our study population was age 25 years. Evidence of tubal pathology identified by HSG was found in 185 patients in the sub-fertile group (67.8%). All the serological markers measured in this study had very low sensitivities and negative predictive values in predicting tubal pathology. The specificities for Ani Labsystems IgG, Vircell IgG, Ani Labsystems IgA and positive C. trachomatis DNA to predict tubal pathology were 84, 86, 95 and 98%, respectively, whereas their respective positive predictive values were 73, 76, 81 and 80%.

**Conclusion:**

The prevalence of C. trachomatis in our study population in Rwanda appears to be low and women aged 25 years are more likely to have genital infection with C. trachomatis. Since serological testing for Chlamydia shows an excellent negative predictive value for lower genital tract infection, specific peptide-based serological assays may be of use for screening in low prevalence settings. Our data suggest that C. trachomatis is not the primary pathogen responsible for tubal pathology in Rwandan women.

# Sub article: Improved Laboratory Capacity for Vibrio Cholera testing to support outbreak control in Mozambique: Contributions of the Field Epidemiology and Laboratory Training Program

**Published:** 2015-07-22

**Authors:** Khalid Azam

**Affiliations:** 1 Mozambique Field Epidemiology and Laboratory and Training Program

## Abstract

**Background:**

In 2010, Mozambique initiated a Field Epidemiology and Laboratory Training Program (FELTP). The program aims to build applied epidemiology and laboratory capacity through joint training and field activities involving both epidemiology and laboratory trainees. Despite annual outbreaks of Cholera in Mozambique, laboratory capacity for Vibrio Cholera (Vc) testing remains limited to reference laboratories (RL) in the capital Maputo. For Cholera outbreaks occurring elsewhere, specimens must be packaged and transported to the RL. An outbreak of Diarrheal illness suspected of Cholera was reported from Cuamba District (Cd) in February 2012 and an FELTP team, consisting of 1 epidemiology and 1 laboratory resident, was deployed to Cd to investigate.

**Methods:**

The FELTP lab resident received just-in-time training in Vc identification methods from RL staff before deploying, and brought necessary supplies to Cd for Vc identification. Specimens were collected from suspect cases, plated on culture media, and incubated at the District lab for identification.

**Results:**

A total of 7 rectal swab specimens were collected and tested in Cd, and all were compatible with Vc. These results were immediately reported to the local epidemiology unit, further informing the ongoing investigation. The estimated improvement in timeliness resulting in diagnostic capability at the District level was at least 5 days. The FELTP lab resident also trained District lab staff in methods for Vc diagnosis. The isolated colonies were later tested by the RL in Maputo and were confirmed as Vc 01, serotype Ogawa.

**Conclusion:**

In this outbreak investigation, laboratory capacity for Vc testing at the district level was established by the FELTP team, resulting in improved timeliness of diagnosis and facilitating the epidemiologic investigation and related disease control efforts. Joint field deployment of both lab and epidemiology residents in outbreak investigations may help overcome limited lab capacity at the provincial levels in Mozambique.

# Sub article: Evaluation of Dried Tube Specimens as an alternative approach for a Proficiency Testing Program, Rwanda- 2012

**Published:** 2015-07-22

**Authors:** Uwimana Jean Marie Vianney

**Affiliations:** 1 Rwanda Field Epidemiology and Laboratory and Training Program

## Abstract

**Background:**

Proficiency Testing (PT) is one of the components of an external quality assessment scheme for HIV Rapid Test (HIV-RT) allowing laboratories to monitor and improve the quality of testing. The Dried Tube Specimen (DTS) approach was developed by CDC to address some of the challenges of using traditional specimen types including availability and maintenance of cold chain. The procedure was piloted for the first time in Rwanda in 2011. Since then, testers for HIV-RT have been trained in preparation for implementation of the procedure countrywide. The current study was to evaluate the feasibility of this new approach by comparing the testing performance of laboratory technicians and non-laboratory technicians using DTS PT panels and assessing the most effective approach to collect PT results from sites and provide feedback and corrective action in a timely manner.

**Methods:**

The DTS panels were prepared at NRL using plasma specimens with different HIV reactivity (negative, positive, and weak positive). Sixty-seven participants received 5 tubes of DTS each in 35 different testing sites countrywide. The DTS were sent to the site with instructions on re-hydration, testing and result submission. Participants were assessed based on correct HIV reactivity identification for each of the five DTS panels received and scored out of 100. Results were analyzed using Epi-Info.

**Results:**

Overall participation rate was 95.4%. Approximately 55% of the participants were laboratory technicians and 45% were non-lab technicians. Ninety-eight percent of the participants sent results by hard copy and 63% sent by hard copy and e-mail. The overall mean score was 99%. There were no significant differences in scores between the two groups with the laboratory technicians scoring 98.8% % while the non-laboratory staff scored 99.3%.

**Conclusion:**

DTS is a feasible alternative specimen type for proficiency testing programs for monitoring the quality of HIV testing and performance of testers. All trained testers scored highly in the DTS PT and the results show non-laboratory technicians can perform HIV Rapid testing at par with laboratory technicians. It is important to increase compliance to above 95.4%. The use of email as a feasible and cost effective means for result submission and feedback should be enhanced.

# Sub article: Progress towards Laboratory Accreditation in Rwanda, 2012

**Published:** 2015-07-22

**Authors:** Innocent Nzabahimana

**Affiliations:** 1 Rwanda Field Epidemiology and Laboratory and Training Program

## Abstract

**Background:**

The accreditation preparedness under Stepwise Laboratory Improvement Process Towards Accreditation (SLIPTA) uses a checklist covering the twelve Quality System Essentials (QSEs), with stepwise progress scaled from 0 to 5 stars. Accreditation preparedness is achieved when the laboratories obtain 5 stars representing > 95% compliance with the checklist. After that the laboratory is then able to strive and apply for full accreditation using the binary pass/fail system. In Rwanda, in January 2010, five central referral laboratories were selected by the MoH for participation in the SLIPTA process with the support of the Stepwise Laboratory Management Towards Accreditation (SLMTA) program. To decentralize the process, 5 satellite laboratories with a local mentoring and Performance Based Financing (PBF) support were enrolled in January 2012 under a regional World Bank project. This assessment aimed to show the progress of the laboratory accreditation process in Rwanda with and without the additional mentoring and PBF support systems.

**Methods:**

For the 5 central laboratories a baseline laboratory audit was conducted in January 2010. Three SLMTA workshops were held during 2010. The SLMTA exit audit was conducted in May 2011 and an accreditation readiness audit took place in May 2012. Audits were conducted by a team of independent laboratory experts from ASCP, ICAP and CDC. For the decentralized SLMTA naive laboratories 2 audits have been held: Baseline in December 2011 and interim in March 2012. In addition to the SLMTA training, quarterly site visit combined with mentorship and Performance Based Financing (PBF) was used to support the satellite laboratories.

**Results:**

All the laboratories started at zero or 1 star before the accreditation process. After the May 2012 (two years after enrollment) assessment of central laboratories, four laboratories had achieved 3 stars and one laboratory 3 stars. The process was much faster in the satellite laboratories where after nine months since enrollment, three satellite laboratories had achieved 3 stars while two were at 2 stars.

**Conclusion:**

The laboratories have shown a steady improvement in the accreditation process. The number of stars represents a significant improvement in quality system essentials, a measure that demonstrates strengthened laboratory system in Rwanda. However as yet, no laboratory has achieved 5 stars and there is need to continue with the accreditation process. We recommend strengthening mentorship, PBF in satellite laboratories and initiation of the same at central level.

# Sub article: Antibiotic Resistance Rates of Enteric Pathogens isolated in Rwanda, 2012

**Published:** 2015-07-22

**Authors:** Innocent Nzabahimana

**Affiliations:** 1 Rwanda Field Epidemiology and Laboratory and Training Program

## Abstract

**Background:**

Bacterial infections continue to be a leading cause of morbidity and mortality worldwide; even as the burden of antimicrobial remains substantial. The aim of this study was to establish the prevalence of enteric pathogens circulating in Rwanda, and determine their antibiotic resistance rates and patterns.

**Methods:**

A cross-sectional study was performed in the 45 District hospital laboratories of Rwanda from January to December 2012. Specimens were collected from patients with Diarrheal disease and transported to the National Reference Laboratory (NRL) using standard transport media for stool and blood. Cultures were performed according to standard microbiological procedures. Isolates were identified by conventional methods and the antibiotic susceptibility profiles determined by the standard disc diffusion method according to Clinical Laboratory Standard Institute (CLSI) guidelines. Results were analyzed using Epi-info version 3.5.1.

**Results:**

A total of 278 specimens were collected during the 12 month period. Of these, 158 were blood and 120 were stool specimens. Sixty-nine (26.7%) of the specimens were cultured positive, and then tested for antimicrobial susceptibility. Twenty-eight Salmonella typhi (10.8%), two *Salmonella Typhimirium* (0.7%), three *Shigella dysenteria* type I (1,1%), twenty one Vibrio Cholerae 01 Inaba (8.1%), nine *Shigella flexneri* (3.4%), six *Shigella Sonnei* (2,3%) species were isolated. *S.Typhi* isolates were found to be susceptible to cefotaxime (100%), ciprofloxacin (97%), ceftazidime (85.8%) and resistant to Nalidixic acid (90.1%), co-trimoxazole (88.6%), ampicillin (86.1%) and chloramphenicol (76.5%). Shigella spp, were 100% susceptible to ciprofloxacin, Cefotaxime, Ceftazidime and Nalidixic acid. *Vibrio Cholerae* inaba, showed 100% susceptibility to ciprofloxacin and tetracyclin, 86%; Nalidixic acid, 83% for ampicillin, 88% for chloramphenicol and 91% for Cotrimoxazole

**Conclusion:**

Recommended antibiotics for bacterial Diarrheal disease treatment in Rwanda are Cefotaxime and Ciprofloxacine for Salmonella sp. infections and ciprofloxacin, Cefotaxime, Ceftazidime or Nalidixic acid for Shigella sp. infections, and Ciprofloxacine or tetracycline for Cholera. The high rates of antimicrobial resistance among recommended first line antibiotics, highlights the need to review the treatment guidelines for bacterial Diarrheal diseases. Monitoring of antibiotics susceptibility patterns in Rwanda should also be continued.

# Sub article: The Role of Leader Behavior on Follower Performance. A case study of Kawempe Health Centre 4

**Published:** 2015-07-22

**Authors:** Musa Sekamatte

**Affiliations:** 1 Uganda Field Epidemiology and Training Program

## Abstract

**Background:**

Leader behavior has always driven organizational bottom line performance. While organizations continue to seek overall performance improvements, a key internal consideration is to determine if the leaders’ influence is felt by followers as to perform their duties based on the leader influence. The study sought to help leaders understand how their behavior affects the follower performance and helps the followers appreciate the characteristics of good leader behavior. Objectives: To identify the dimensions of leader behavior; determine the level of follower compliance to the leader; establish the linkage between leader behavior and follower performance; identify other determinants of follower performance. Research Questions: What are the dimensions of leader behavior? What is the level of follower compliance to the leader? What is the link between leader behavior and follower performance? What are other determinants of follower performance?

**Methods:**

A qualitative approach which involved focus group discussion (FGD) and survey interviews was conducted. 6 FGDs of 6 chosen hospital departments and 6 interview surveys were conducted with 6 heads of the chosen departments. A total of 60 respondents in FGDs and 6 leaders for interview surveys were considered. Purposive sampling was used because participants had an experience of the subject being investigated. Inclusion criterion was all followers in chosen departments and leaders of the same departments. Data from FGDs and interviews then captured on a recorder and then transcribed later onto paper. Data analyzed manually by thematic analysis technique where major themes were identified.

**Results:**

Dimensions of leader behavior found included: Support, Goal Emphasis, and Interaction Facilitation. The dimension of Work facilitation was not found. Followers easily followed instructions from leaders with democratic and engaging leader behavior since it had increase of follower readiness. Positional leader behavior led to low follower readiness thus poor follower compliance and poor performance. Engaging and democratic leader behavior showed high follower readiness thus great follower performance. Positional leader behavior led to low follower readiness thus poor follower performance. Other determinants of follower performance include staff retreats, appreciation of work done and motivation facilitation.

**Conclusion:**

There is need for support, work facilitation, goal emphasis, interaction facilitation, democratic, engaging and no positional leader behavior.

# Sub article: High compliance with Newborn Community-to-facility Referral: An opportunity to improve newborn survival in Eastern Uganda

**Published:** 2015-07-22

**Authors:** Christine Nalwadda

**Affiliations:** 1 Uganda Field Epidemiology and Training Program

## Abstract

**Background:**

Ninety-nine percent of newborn deaths occur in low income countries. To improve neonatal health, WHO and UNICEF recommend home visits to mothers and babies in the first week of life to assess for danger signs (sickness) and counsel caretakers for immediate referral of the sick. However, there is a dearth of data on the level of timely compliance and determinants of newborn referral in sub-Saharan Africa. We assessed timely compliance to newborn referrals made by community health workers (CHWs), and its determinants in rural eastern Uganda.

**Methods:**

In this historical cohort study, we retrospectively followed-up all newborns referred to health facilities between September 2009 and August 2011 in Iganga-Mayuge Health Demographic Surveillance Site in eastern Uganda. Timely compliance was defined as caretakers of newborns complying with CHWs’ referral advice within 24 hours. Descriptive and logistic regression data analyses were done.

**Results:**

A total of 724 referred newborns were identified in the CHWs’ registers, of which 700 were successfully traced. Fifty-three percent (373/700) were referred for immunization and postnatal care and 47% (327/700) because of a danger sign (sick). Overall, 640 (91%) caretakers reported to have complied with the referral advice, with fewer, 63% (439/640) complying timely. In addition, 74% (243/327) of the caretakers for sick newborns complied timely. Factors found to be associated with timely referral compliance were: age of mother, with an Adjusted Odds Ratio (AOR) of 0.6 and 95% confidence interval (CI) of [0.4 - 0.9]; the CHW making a reminder visit to the referred newborn shortly after referral (AOR = 1.8 [1.2 -2.5]); and the newborn being sick at the time of referral (AOR= 2.6, [1.9 - 3.6]).

**Conclusion:**

Compliance with CHW community-to- facility newborn referral in this setting was high. Sick newborns, younger mothers and a reminder visit by the CHW to the referred newborn enhanced compliance to newborn referral. Integration of CHWs into maternal and newborn care programs may potentially increase timely care seeking for newborns, thereby contributing to reduction of newborn mortality and accelerate attainment of the fourth millennium development goal.

# Sub article: Determinants of Performance of Community Health workers managing Malaria, Pneumonia and Diarrhea in Wakiso District-Uganda

**Published:** 2015-07-22

**Authors:** James Bagonza

**Affiliations:** 1 Uganda Field Epidemiology and Training Program

## Abstract

**Background:**

Community Health Workers (CHWs) are widely used to provide curative interventions in communities that lack access to health care. Uganda adopted the Integrated Community Case Management (ICCM) policy in 2010 to improve child survival through the management Malaria, pneumonia and Diarrhea using CHWs. There was little documentation on the performance of CHWs treating Malaria, pneumonia and Diarrhea in Uganda. We assessed factors influencing performance of CHWs under the ICCM strategy in Wakiso District -Uganda.

**Methods:**

We conducted a cross sectional study between March and June 2012 among 336 CHWs managing Malaria, pneumonia and Diarrhea in children under five years in Wakiso District. Only CHWs who had worked under the ICCM strategy for more than 6 months by the time of the study were included. Performance was assessed using mixed methods (interviews and record reviews) and composite scores based on the core activities of CHWs under the ICCM program. Core activities included: treating children under five years, referring severely sick children and newborns, home visits, counseling caregivers on home care, recording keeping and community sensitization. We classified performance as good when a CHW scored above 75% and poor when the score was below 75%. Four focus group discussions with CHWs were conducted and data were analyzed manually for content. Descriptive and inferential statistics using odds ratios were done to determine factors influencing performance of CHWs.

**Results:**

Of the 336 respondents, 72% (242/336) were females and the overall level of good performance was 21.7% (73/336). Factors significantly associated with good performance were; being female (OR= 2.67, 95% CI = 1.29 - 5.51), community support (OR = 2.27, 95% CI= 1.16 - 4.35) and regular reporting (OR = 5.6, 95% CI= 1.5- 20). Others were receiving timely feedback (OR = 3.45, 95% CI = 1.69 - 6.9) and drug availability (OR = 3.3, 95% CI = 1.84 - 6.27). Monetary incentives, bicycles, gumboots and T-shirts or uniforms were perceived to influence CHWs’ performance.

**Conclusion:**

Only one in every five CHWs performed optimally. Strategies to improve supply of drugs, community support, reporting, and feedback from the formal health system, are necessary to improve the performance of CHWs.

# Sub article: Improving healthcare delivery to nomads: Enumeration of settlements and monitoring for Polio vaccination campaigns August-September 2012, Maru LGA, Nigeria

**Published:** 2015-07-22

**Authors:** Aishatu Sadiq

**Affiliations:** 1 Nigeria Field Epidemiology and Laboratory and Training Program

## Abstract

**Background:**

Nomadic communities are completely missed or partially covered during immunization plus days IPDs) due to frequent mobility and remoteness of settlements. Low immunization coverage leading to a large susceptible migrant population contributes greatly to spread of poliomyelitis. Reports from independent monitors indicate present local government and ward micro planning is inadequate to identify and reach nomads. Nigeria maintains wild polio virus transmission. Most cases of wild polio virus (WPV) identified in 2012 were among nomadic children. States in the north of the country are the main source of polio infection for other regions in Nigeria and neighboring countries. We revised ward micro plans, conducted a census of nomadic populations and subsequently monitored IPDs.

**Methods:**

We conducted secondary data abstraction of existing ward micro plans and key informant interviews with traditional leaders. Indigenous nomadic guides and data collectors were trained in the use of GPS devices and data entry tools used for the exercise. Missed children, unlisted settlements and unreported acute flaccid paralysis (AFP) cases were identified. 14 teams were dispatched and supervised to 10 wards to gather baseline data on nomadic settlements assess vaccination coverage, under-five population and obtain GPS co-ordinates. Data was entered and analyzed by descriptive analysis with Microsoft excel.

**Results:**

172 nomadic settlements were listed in the micro plan, 111 unlisted settlements were identified, 63 (22%) of total settlements had never been visited by vaccination teams, 47 (17%) of settlements were missed in previous IPDs and 1 case of unreported acute flaccid paralysis (AFP) was identified. During subsequent monitoring 111 (100%) of newly enumerated unlisted settlements were visited by special vaccination teams and ward level micro plans were adequately updated.

**Conclusion:**

Partnership between traditional rulers, religious leaders and immunization teams established and maintained throughout the exercise ensured success of the exercise. Special nomadic teams providing added personnel are essential to ensuring effective vaccination coverage of nomadic communities.

# Sub article: Willingness to Participate in Community- Based Health-Care Financing Scheme by Rural Households- Osun State- Nigeria, 2012

**Published:** 2015-07-22

**Authors:** Aishat Usman

**Affiliations:** 1 Nigeria Field Epidemiology and Laboratory and Training Program

## Abstract

**Background:**

Community-based healthcare financing (CBHF) has been recognized as a convenient payment option for health care systems with wide population coverage. Experience with the scheme at state level suggests high acceptability and effectiveness. There is therefore the need to extend its coverage particularly among the poor and underserved. We assessed willingness to Participate (WTP) in CBHF scheme, and identified factors influencing its adoption within rural population of Osun State, South Western Nigeria.

**Methods:**

We conducted a cross-sectional survey on 450 rural household heads in Osun State, Southwestern, Nigeria using a three step multistage sampling technique. Semi-structured questionnaires was used to collect data on respondents sociodemographic characteristics, income, participation in health care financing schemes (HCFS) and WTP using Dichotomous Method (DCM). The bid used for DCM consisted of five categories USD 3.1, 4.7, 6.3, 7.8 and 9.4. We performed bivariate and multivariate data analysis using STATA 10 software to identify factors influencing WTP.

**Results:**

The mean age of heads of household was 47 ± 10.2years. Two-third of household heads (78.2%) was male and 53.7% (241) were farmers. The mean family size per household was 5 ±3.2 with average income of 62.5USD per month. Three hundred and seventy- three (82.8%) household heads were willing to participate in CBHF scheme. Only 10%(45) had participated in any form of HCFS. The mean acceptable premium per year was USD 4.51 (± USD 1.57). Factors positively influencing WTP were having secondary education [(AOR 3.1, 95% CI = 1.8-5.4], male gender [AOR 4.1, 95% CI =1.4- 12.3], higher income [AOR 1.6, 95% CI =1.3-1.8] and lower age [AOR 0.4, 95% CI = 0.2-0.9].

**Conclusion:**

The findings from this study showed that most household heads were willing to pay for CBHF, but the average premium they were ready to pay was low. Women, householders on low income, the elderly and those with primary or no education were less WTP. There would be the need to subsidize the premiums especially of vulnerable groups when establishing the scheme.

# Sub article: Determinants of Loss to Follow-up in patients initiated on antiretroviral therapy, in a Pharmacovigilance sentinel site cohort, 2004-2012

**Published:** 2015-07-22

**Authors:** Mazvita Naome Muropa

**Affiliations:** 1 South Africa Field Epidemiology and Laboratory and Training Program

## Abstract

**Background:**

The number of Human Immunodeficiency Virus (HIV) infected people eligible for initiation on Antiretroviral Therapy (ART) is increasing. ART programmatic success requires that patients that are initiated on ART remain on treatment and are followed up regularly. Studies in South Africa have shown that there is an increasing incidence of loss to follow-up with each year that patients are on ART. This study investigated the factors associated with being lost to follow-up in a cohort of patients enrolled in a pharmacovigilance study in South Africa between 2004 and 2012.

**Methods:**

This was a retrospective observational cohort study performed at one of the Medunsa National Pharmacovigilance Centre (MNPC) ART sentinel surveillance sites. Loss to follow-up (LTFU) was defined as a patient who has been followed up at the sentinel site, who has not had contact with the health facility for 180 days or more since their last recorded expected date of return or if there were 180 days or more between the expected date of return and the next clinic visit.

**Results:**

The outbreak investigation showed that the two cases in the state were siblings aged 2 and 8 years old, they both reported a history of multiple doses of OPV, had no history of travel, and no contact with symptomatic persons. The most recent NID administrative data showed that coverage in their county of children <5 years old was 104%. Analysis of the interviews with NID staff showed that 40% of vaccinators had no knowledge of AFP, OPV, polio disease, basic NID protocols. Twenty-two percent of field assistants had no knowledge of those subjects.

**Conclusion:**

Out of 595 patients, 66% (n = 393) were female and 23.4% (n = 139) were LTFU. The median time on ART before LTFU was 655 days (1.8years) (Interquartile range: 392 days (1.1years) - 1059 days (2.9years). Factors associated with becoming lost to follow-up included not having a partner (Hazard Ratio (HR): 2.3; p value = 0.025), being self-employed (HR: 16.9; p value = 0.000), baseline CD4 count > 200 cells/ml (HR: 4.5; p value = 0.000), and detectable last known Viral Load (VL) (HR: 2.5; p value = 0.001). Patients that had a last known CD4 count >200 cells/ml had a lower risk (HR: 0.5; p value = 0.008) of becoming lost to follow-up than those that had a last known CD4 count <200 cells/ml.

# Sub article: Assessment of a Specialist hospital laboratory in Effutu municipality in Ghana, 2013

**Published:** 2015-07-22

**Authors:** Alexander Asamoah

**Affiliations:** 1 Ghana Field Epidemiology and Laboratory and Training Program

## Abstract

**Background:**

The medical laboratory has a central coordination in health care delivery and requires accreditation, constant monitoring and assessment to ensure quality results and appropriate public health action. In Ghana, hospital laboratories did not require assessment-based accreditation to operate and therefore none has been accredited. The process of accreditation has just begun for both hospital and private (community) medical laboratories in line with the introduction of a new guideline. In Effutu municipality, a newly established specialist hospital laboratory which serves as a reference laboratory for six other facilities in the municipality is also preparing for the accreditation. In view of this, this study was conducted to assess the readiness of the specialist hospital laboratory towards accreditation.

**Methods:**

We conducted a cross sectional survey in the Effutu municipality from June 20 to July 5, 2013. We used the World Health Organization laboratory strengthening checklist to collect information on twelve areas of laboratory management. Each area assessed was scored and the laboratory rated using a scale from fifth tier (0-125) which is the lowest to first tier (236-250) which is the highest.

**Results:**

The total assessment score was 33.6% (84/250). There were no evidences of management reviews, internal audit, client management and occurrence management. The measures of documents and records were 7.7% (2/26). Quality control assessments scored 19.5% (8/41). Measures of organization and personnel, purchasing and inventory, equipment management and information management scored 35.3% (6/17), 38.2% (13/34), 41.4% (12/29) and 41.7% (5/12) respectively. Facilities and safety measures scored 65% (30/46) with the highest relative score of 35.7% (30/84). The laboratory was rated as fifth tier.

**Conclusion:**

The laboratory performed poorly in the assessment and at the lowest level of strength. We recommended that the hospital management should provide training for laboratory staff in quality laboratory management and educate them to ensure proper documentation and records keeping for future references. The latter has been done.

# Sub article: Re-Emergence of Polio Disease in South Sudan

**Published:** 2015-07-22

**Authors:** David Deng

**Affiliations:** 1 Sudan Immunization and Field Epidemiology Training Project

## Abstract

**Background:**

Acute flaccid paralysis (AFP) is characterized by limb weakness and is often indicative of polio disease. Due to annual and multiple national immunization days (NIDs), South Sudan had no polio cases since 2009. However, in September 2013, 3 cases were diagnosed. These cases followed diagnosis of polio disease in the neighboring countries of Kenya and Ethiopia. Two cases of the 3 cases were residents of Northern Bahr El Ghazal state, where the State Ministry of Health (SMoH) conducted an outbreak investigation and an assessment of NID vaccinators.

**Methods:**

The SMoH and its partners conducted an outbreak investigation per standard World Health Organization and MoH guidelines. Simultaneously, we conducted face-to-face interviews with staff that were responsible for implementing the NIDs: vaccinators and field assistants. We used a 6-question interview guide to gauge NID staff knowledge of AFP, polio, oral polio vaccine (OPV), and NID protocols.

**Results:**

The outbreak investigation showed that the two cases in the state were siblings aged 2 and 8 years old, they both reported a history of multiple doses of OPV, had no history of travel, and no contact with symptomatic persons. The most recent NID administrative data showed that coverage in their county of children <5 years old was 104%. Analysis of the interviews with NID staff showed that 40% of vaccinators had no knowledge of AFP, OPV, polio disease, basic NID protocols. Twenty-two percent of field assistants had no knowledge of those subjects.

**Conclusion:**

Re-emergence of polio disease in South Sudan is a public health emergency. The outbreak investigation and NID staff interview results show that 1) there is limited knowledge of the importance of NIDs in the community; 2) frontline public health staff are not knowledgeable enough to educate the public they vaccinate during the NIDs; and 3) public health practice and policy in South Sudan should focus on longer-term and more comprehensive training of their frontline staff for future NIDs.
